# Supplementary material for: Long-read sequencing across the C9orf72 ‘GGGGCC’ repeat expansion: implications for clinical use and genetic discovery efforts in human disease
Source: Mol Neurodegener. 2018 Aug 21;13:46. doi: 10.1186/s13024-018-0274-4 (PMC6102925; doi:10.1186/s13024-018-0274-4)
Supplement: Supplementary file 1 — Figure S1. Repeat-containing plasmids have a large repeat size distribution. Figure S2. Sanger sequencing confirms the SCA36 repeat plasmid contains at least 37 repeats. Figure S3. Individual Sequel and MinION read(s) across the C9orf72 repeat region aligned to the reference genome and hand curated. Data S1. RS II consensus sequence in the C9-774 repeat region is 99.77% accurate, when compared to the plasmid reference sequence. Data S2. MinION consensus sequence in the C9-774 repeat region is 26.55% accurate, when compared to the plasmid reference sequence. Data S3. MinION read covering the non-pathogenic allele. Data S4. Sequel read covering the non-pathogenic allele. Data S5. Sequel read that did not extend through repeat, but contains approximately 30 repeats. Data S6. Sequel read covering approximately 69 repeats. Data S7. Sequel read that did not extend through the repeat, but contains approximately 912 repeats. Data S8. Sequel read covering 1324-repeat allele. Data S9. PacBio Sequel consensus sequence by Long Amplicon Analysis (LAA2). Data S10. Plasmid backbones used to separate reads. Data S11. Sequences adjacent to the C9orf72 repeat region used to identify on-target reads from the PacBio No-Amp Targeted Sequencing approach. (DOCX 3982 kb) [file 13024_2018_274_MOESM1_ESM.docx]

**Supplemental Materials**

**
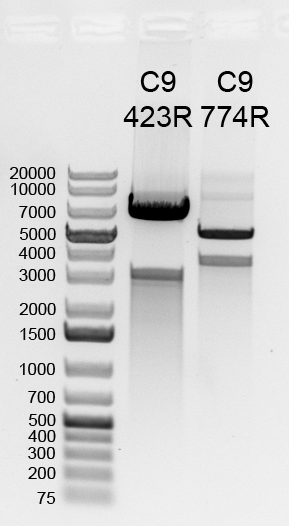

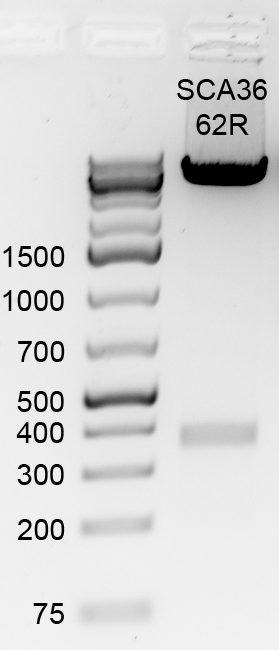

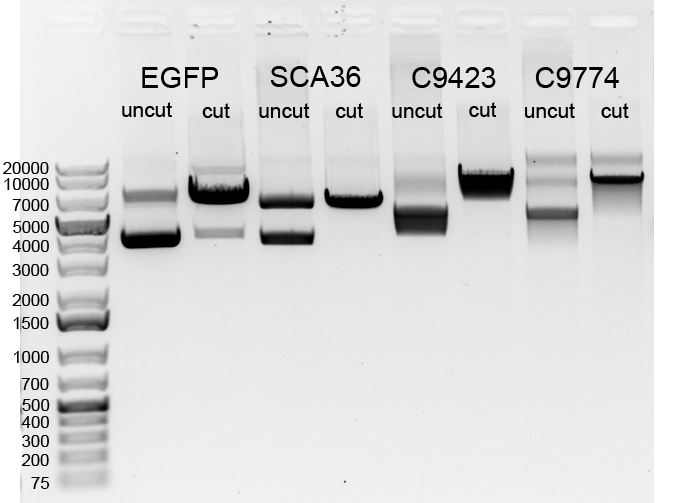
**

a.

b.

c.

**
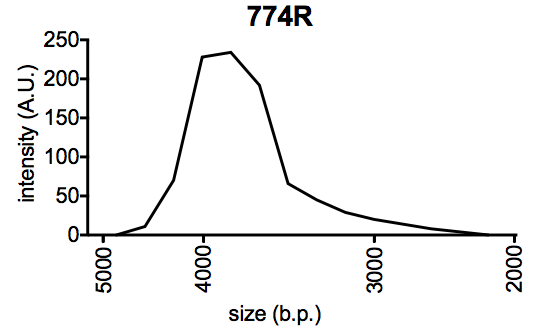

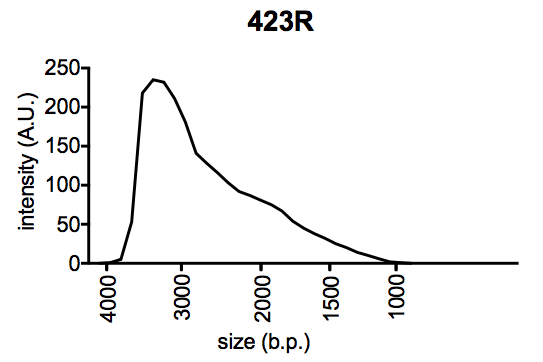
**

d.

**Supplemental figure 1. Repeat-containing plasmids have a large repeat size distribution.** We cloned the SCA36 ‘GGCCTG’ and *C9orf72* ‘GGGGCC’ repeats into plasmids to compare the PacBio and Oxford Nanopore Technologies’ sequencing platforms in repeat regions. We aimed to clone 62 SCA36 ‘GGCCTG’, and 423 and 774 *C9orf72* ‘GGGGCC’ repeats, respectively. We also included a non-repeat-containing plasmid, which included *EGFP*. **(a)** Each of the four plasmids was run on a 2% agarose gel before being linearized (uncut) and after being linearized (cut) by restriction enzymes (MluI for C9-423 and AvrII for the others). Each uncut plasmid had a band for supercoiled and relaxed conformations. The C9-774 plasmid also had a clear third band, identifying nicked plasmids. The cut C9-423 and C9-774 repeat plasmids demonstrated a distribution of plasmid sizes, based on the respective smears. The SCA36 plasmid had a single band and the *EGFP* plasmid had two bands because it was partially digested. **(b)** We cut the SCA36 plasmid using two restriction enzymes (HindIII and BamHI) up and downstream of the repeat region in a separate digest to measure each plasmid’s repeat size. The upstream region is an additional 103 nucleotides while there are an additional 39 nucleotides downstream. The larger band is the primary portion of the plasmid backbone, while the smaller band is the repeat region, plus the additional 142 nucleotides. Based on an estimated size of 400 nucleotides for the repeat band, the mean repeat size is estimated at (400 – 142)/6 = 43 repeats. The band from the repeat region is partially smeared, indicating the repeat was not fully stable. This closely mirrored our observations on the RS II and MinION in Figure 4 of the main text. **(c)** Like the SCA36 plasmids, we cut the C9-423 and C9-774 plasmids up and downstream of the repeat region to assess repeat size and stability. Using the same restriction enzymes (HindIII and BamHI), there are an additional 117 and 101 nucleotides up and downstream of both the C9-423 and C9-774 repeat regions. The band for the C9-423 repeat region is highly smeared, but the primary population ran at approximately 3000 nucleotides, estimating most plasmids have a repeat size of (3000 – 218)/6 = 464 repeats. Estimating the C9-774 primary repeat band size at 4000 nucleotides, the estimated repeat size is (4000 – 218)/6 = 630 repeats. This also differs from the distribution in Figure 4 of the main text, which had a median size of approximately 400 repeats. **(d)** To analytically determine the repeat size distribution for the C9-423 and C9-774 plasmids, we performed a gel intensity analysis. The C9-423 repeat size is highly variable, ranging from approximately 1000 to 3800 nucleotides. The C9-774 repeat size is less variable, ranging from approximately 3500 to 4500, with a long tail on the shorter end. This may be because of the different plasmids the repeats were cloned into. These data demonstrate the variability in the plasmid repeat sizes.

**
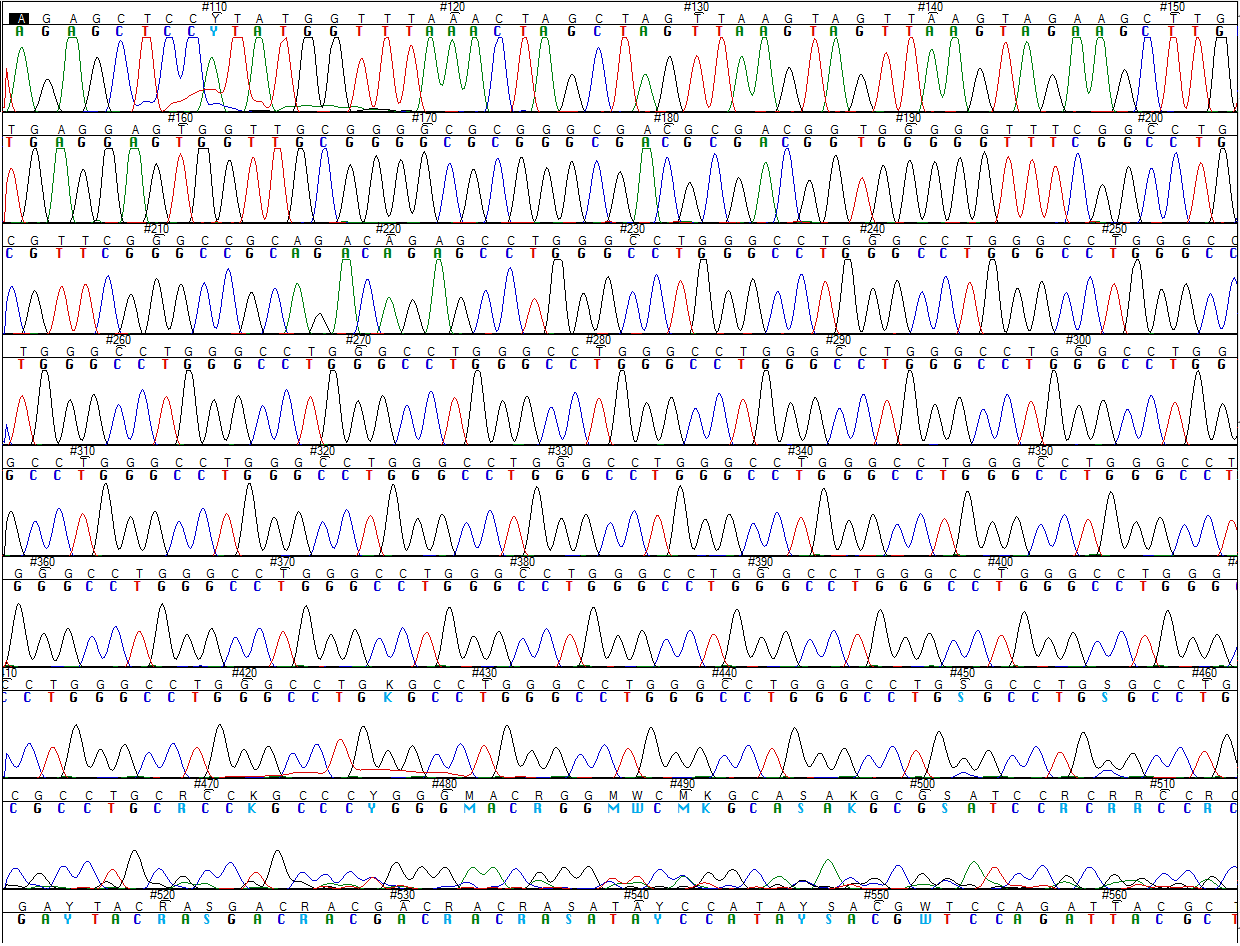
**

**Supplemental figure 2. Sanger sequencing confirms the SCA36 repeat plasmid contains at least 37 repeats.** We Sanger sequenced the SCA36 repeat region to determine the estimated minimum length for the SCA36 ‘GGCCTG’ repeat region. Sequence traces were clear through approximately 37 repeats, when the sequence trace became indeterminate, indicating the expected minimal length for the SCA36 repeat region.

**
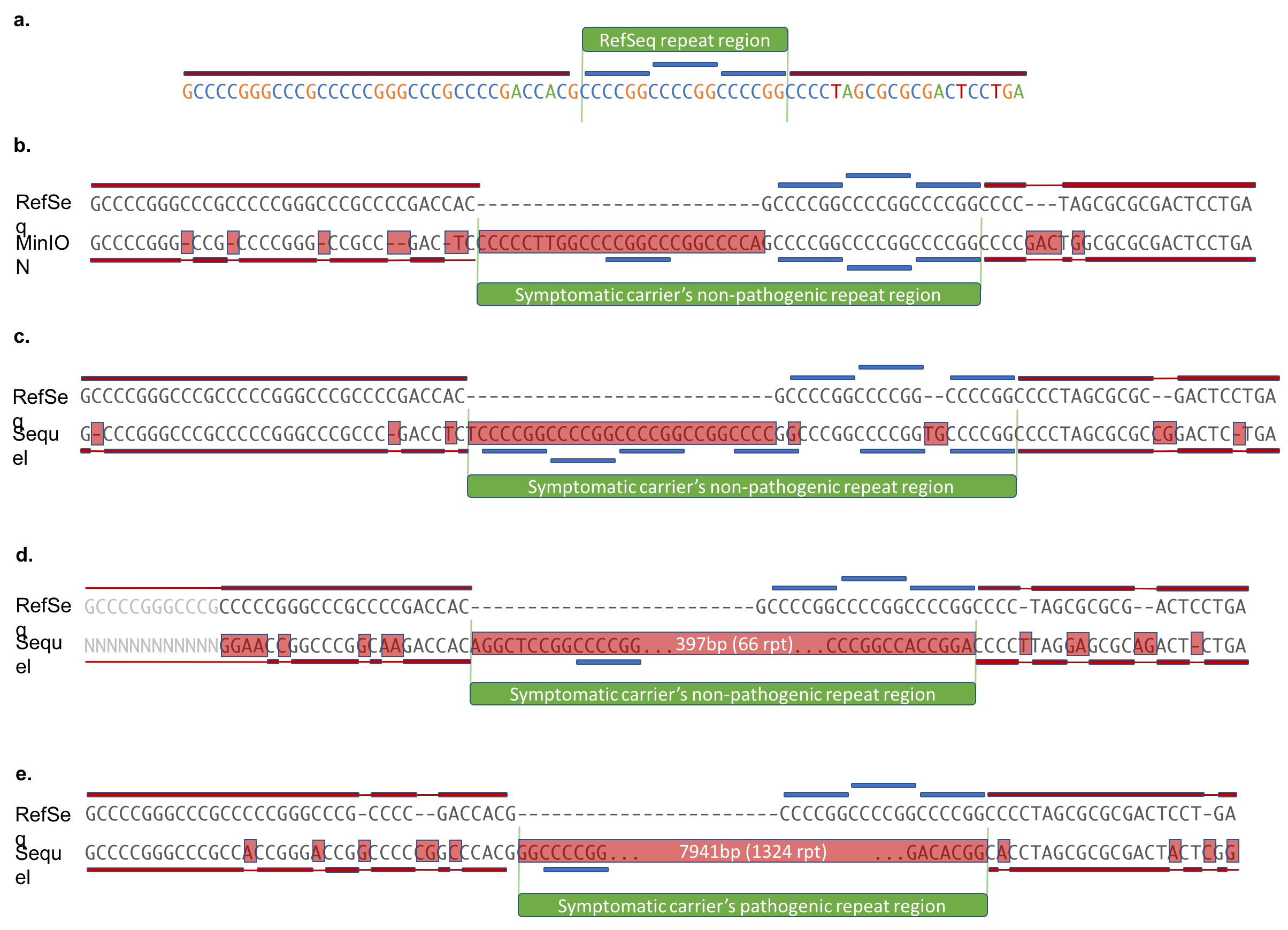
**

**Supplemental figure 3. Individual Sequel and MinION read(s) across the *C9orf72* repeat region aligned to the reference genome and hand curated.** Both the Sequel and MinION covered a non-pathogenic allele that are highly concordant with each other, and with the estimated repeat size from a fragment analysis. We also obtained three Sequel reads reading partially through an expanded allele, and a fourth that bridged the repeat region with 1324 repeats. **(a)** The human genome reference sequence (hg38) contains three G_4_C_2_ repeats. *C9orf72* is on the ‘-‘ strand of the reference genome, representing the repeat as C_4_G_2_. We identified specific “landmarks” before and after the repeat region in the reference sequence to properly locate the repeat region in the reads, and to hand curate the alignments. Landmarks are identified by red bars up and downstream of the repeat region. All segments matching the C_4_G_2_ motif are identified by blue bars, and the defined repeat region is marked by green. **(b)** The MinION sequence is highly similar to the reference sequence both up and downstream of the defined repeat region, closely matching all of the primary landmarks, where mismatches are highlighted in red. There is a net gain of 22 nucleotides, when considering the entire sequence shown, but 27 within the defined repeat region; this equates to approximately 4 or 5 additional repeats. **(c)** The Sequel reads were highly similar to the reference sequence both up and downstream of the defined repeat region, closely matching all of the primary landmarks. Only one of the four reads is shown. There are 48 nucleotides within the defined repeat region, equating to exactly 8 repeats, which concurs with our fragment analysis. **(d)** A Sequel read that captured approximately 417 nucleotides (approximately 69 repeats) within the repeat region. It is ambiguous whether this read actually bridges the repeat region because the left-hand end of the read closely matches sequence adjacent to the repeat region, as demonstrated in the figure. **(e)** A sequel read that spanned the entire repeat region of a pathogenic allele contains approximately 1324 repeats (7941 nucleotides).

**Supplemental data 1. RS II consensus sequence in the C9-774 repeat region is 99.77% accurate, when compared to the plasmid reference sequence.** We aligned the RS II consensus sequence for just the repeat region to the plasmid’s reference sequence. The sequences were 99.77% similar. We include the entire C9-774 RS II consensus sequence. Defined repeat region highlighted in yellow.

>C9-774_RSII_consensus_sequence CTAGGCTTTTGCAAAAAGCTCCCGGGAGCTTGTATATCCATTTTCGGATCTGATCAGCACGTGTTGACAATTAATCATCGGCATAGTATATCGGCATAGTATAATACGACAAGGTGAGGAACTAAACCATGGCCAAGCCTTTGTCTCAAGAAGAATCCACCCTCATTGAAAGAGCAACGGCTACAATCAACAGCATCCCCATCTCTGAAGACTACAGCGTCGCCAGCGCAGCTCTCTCTAGCGACGGCCGCATCTTCACTGGTGTCAATGTATATCATTTTACTGGGGGACCTTGTGCAGAACTCGTGGTGCTGGGCACTGCTGCTGCTGCGGCAGCTGGCAACCTGACTTGTATCGTCGCGATCGGAAATGAGAACAGGGGCATCTTGAGCCCCTGCGGACGGTGCCGACAGGTGCTTCTCGATCTGCATCCTGGGATCAAAGCCATAGTGAAGGACAGTGATGGACAGCCGACGGCAGTTGGGATTCGTGAATTGCTGCCCTCTGGTTATGTGTGGGAGGGCTAAGCACTTCGTGGCCGAGGAGCAGGACTGACACGTGCTACGAGATTTCGATTCCACCGCCGCCTTCTATGAAAGGTTGGGCTTCGGAATCGTTTTCCGGGACGCCGGCTGGATGATCCTCCAGCGCGGGGATCTCATGCTGGAGTTCTTCGCCCACCCCAACTTGTTTATTGCAGCTTATAATGGTTACAAATAAAGCAATAGCATCACAAATTTCACAAATAAAGCATTTTTTTCACTGCATTCTAGTTGTGGTTTGTCCAAACTCATCAATGTATCTTATCATGTCTGTATACCGTCGACCTCTAGCTAGAGCTTGGCGTAATCATGGTCATAGCTGTTTCCTGTGTGAAATTGTTATCCGCTCACAATTCCACACAACATACGAGCCGGAAGCATAAAGTGTAAAGCCTGGGGTGCCTAATGAGTGAGCTAACTCACATTAATTGCGTTGCGCTCACTGCCCGCTTTCCAGTCGGGAAACCTGTCGTGCCAGCTGCATTAATGAATCGGCCAACGCGCGGGGAGAGGCGGTTTGCGTATTGGGCGCTCTTCCGCTTCCTCGCTCACTGACTCGCTGCGCTCGGTCGTTCGGCTGCGGCGAGCGGTATCAGCTCACTCAAAGGCGGTAATACGGTTATCCACAGAATCAGGGGATAACGCAGGAAAGAACATGTGAGCAAAAGGCCAGCAAAAGGCCAGGAACCGTAAAAAGGCCGCGTTGCTGGCGTTTTTCCATAGGCTCCGCCCCCCTGACGAGCATCACAAAAATCGACGCTCAAGTCAGAGGTGGCGAAACCCGACAGGACTATAAAGATACCAGGCGTTTCCCCCTGGAAGCTCCCTCGTGCGCTCTCCTGTTCCGACCCTGCCGCTTACCGGATACCTGTCCGCCTTTCTCCCTTCGGGAAGCGTGGCGCTTTCTCATAGCTCACGCTGTAGGTATCTCAGTTCGGTGTAGGTCGTTCGCTCCAAGCTGGGCTGTGTGCACGAACCCCCCGTTCAGCCCGACCGCTGCGCCTTATCCGGTAACTATCGTCTTGAGTCCAACCCGGTAAGACACGACTTATCGCCACTGGCAGCAGCCACTGGTAACAGGATTAGCAGAGCGAGGTATGTAGGCGGTGCTACAGAGTTCTTGAAGTGGTGGCCTAACTACGGCTACACTAGAAGAACAGTATTTGGTATCTGCGCTCTGCTGAAGCCAGTTACCTTCGGAAAAAGAGTTGGTAGCTCTTGATCCGGCAAACAAACCACCGCTGGTAGCGGTGGTTTTTTTGTTTGCAAGCAGCAGATTACGCGCAGAAAAAAAGGATCTCAAGAAGATCCTTTGATCTTTTCTACGGGGTCTGACGCTCAGTGGAACGAAAACTCACGTTAAGGGATTTTGGTCATGAGATTATCAAAAAGGATCTTCACCTAGATCCTTTTAAATTAAAAATGAAGTTTTAAATCAATCTAAAGTATATATGAGTAAACTTGGTCTGACAGTTACCAATGCTTAATCAGTGAGGCACCTATCTCAGCGATCTGTCTATTTCGTTCATCCATAGTTGCCTGACTCCCCGTCGTGTAGATAACTACGATACGGGAGGGCTTACCATCTGGCCCCAGTGCTGCAATGATACCGCGAGACCCACGCTCACCGGCTCCAGATTTATCAGCAATAAACCAGCCAGCCGGAAGGGCCGAGCGCAGAAGTGGTCCTGCAACTTTATCCGCCTCCATCCAGTCTATTAATTGTTGCCGGGAAGCTAGAGTAAGTAGTTCGCCAGTTAATAGTTTGCGCAACGTTGTTGCCATTGCTACAGGCATCGTGGTGTCACGCTCGTCGTTTGGTATGGCTTCATTCAGCTCCGGTTCCCAACGATCAAGGCGAGTTACATGATCCCCCATGTTGTGCAAAAAAGCGGTTAGCTCCTTCGGTCCTCCGATCGTTGTCAGAAGTAAGTTGGCCGCAGTGTTATCACTCATGGTTATGGCAGCACTGCATAATTCTCTTACTGTCATGCCATCCGTAAGATGCTTTTCTGTGACTGGTGAGTACTCAACCAAGTCATTCTGAGAATAGTGTATGCGGCGACCGAGTTGCTCTTGCCCGGCGTCAATACGGGATAATACCGCGCCACATAGCAGAACTTTAAAAGTGCTCATCATTGGAAAACGTTCTTCGGGGCGAAAACTCTCAAGGATCTTACCGCTGTTGAGATCCAGTTCGATGTAACCCACTCGTGCACCCAACTGATCTTCAGCATCTTTTACTTTCACCAGCGTTTCTGGGTGAGCAAAAACAGGAAGGCAAAATGCCGCAAAAAAGGGAATAAGGGCGACACGGAAATGTTGAATACTCATACTCTTCCTTTTTCAATATTATTGAAGCATTTATCAGGGTTATTGTCTCATGAGCGGATACATATTTGAATGTATTTAGAAAAATAAACAAATAGGGGTTCCGCGCACATTTCCCCGAAAAGTGCCACCTGACGTCGACGGATCGGGAGATCTCCCGATCCCCTATGGTGCACTCTCAGTACAATCTGCTCTGATGCCGCATAGTTAAGCCAGTATCTGCTCCCTGCTTGTGTGTTGGAGGTCGCTGAGTAGTGCGCGAGCAAAATTTAAGCTACAACAAGGCAAGGCTTGACCGACAATTGCATGAAGAATCTGCTTAGGGTTAGGCGTTTTGCGCTGCTTCGCGATGTACGGGCCAGATATACGCGTTGACATTGATTATTGACTAGTYGAGKCCCTTTCGTCTTCACTCGAGTTTACTCCCTATCAGTGATAGAGAACGTATGTCGAGTTTACTCCCTATCAGTGATAGAGAACGATGTCGAGTTTACTCCCTATCAGTGATAGAGAACGTATGTCGAGTTTACTCCCTATCAGTGATAGAGAACGTATGTCGAGTTTACTCCCTATCAGTGATAGAGAACGTATGTCGAGTTTATCCCTATCAGTGATAGAGAACGTATGTCGAGTTTACTCCCTATCAGTGATAGAGAACGTATGTCGAGGTAGGCGTGTACGGTGGGAGGCCTATATAAGCAGAGCTCGTTTAGTGAACCGTCAGATCGCCAAGCTTAGTACTCGCTGAGGGTGAACAAGAAAAGACCTGATAAAGATTAACCAGAAGAAAACAAGGAGGGAAACAACCGCAGCCTGTAGCAAGCTCTGGAACTCAGGAGTCGCGCGCTAGGGGCCGGGGCCGGGGCCGGGGCCGGGGCCGGGGCCGGGGCCGGGGCCGGGGCCGGGGCCGGGGCCGGGGCCGGGGCCGGGGCCGGGRCCGGGGCCGGGGCCGGGGCCGGGGCCGGGGCCGGGGCCGGGGCCGGGGCCGGGGCCGGGGCCGGGGCCGGGGCTGGGGCCGGGGCCGGGGCCGGGGCCGGGGCCGGGGCCGGGGCCGGGGCCGGGGCCGGGGCCGGGGCCGGGGCCGGGGCCGGGGCCGGGGCCGGGGCCGGGGCCGGGGCCGGGGCCGGGGCCGGGGCCGGGGCCGGGGCCGGGGCCGGGGCCGGGGCCGGGGCCGGGGCCGGGGCCGGGGCCGGGGCCGGGGCCGGGGCCGGGGCCGGGGCCGGGGCCGGGGCCGGGGCCGGGGCCGGGGCCGGGGCCGGGGCCGGGGCCGGGGCCGGGGCCGGGGCCGGGGCCGGGGCCGGGGCCGGGGCCGGGGCCGGGGCCGGGGCCGGGGCCGGGGCCGGGGCCGGGGCCGGGGCCGGGGCCGGGGCCGGGGCCGGGGCCGGGGCCGGGGCCGGGGCCGGGGCCGGGGCCGGGGCCGGGGCCGGGGCCGGGGCCGGGGCCGGGGCCGGGGCCGGGGCCGGGGCCGGGGCCGGGGCCGGGGCCGGGGCCGGGGCCGGGGCCGGGGCCGGGGCCGGGGCCGGGGCCGGGGCCGGGGCCGGGGCCGGGGCCGGGGCCGGGGCCGGGGCCGGGGCCGGGGCCGGGGCCGGGGCCGGGGCCGGGGCCGGGGCCGGGGCCGGGGCCGGGGCCGGGGCCGGGGCCGGGGCCGGGGCCGGGGCCGGGGCCGGGGCCGGGGCCGGGGCCGGGGCCGGGGCCGGGGCCGGGGCCGGGGCCGGGGCCGGGGCCGGGGCCGGGGCCGGGGCCGGGGCCGGGGCCGGGGCCGGGGCCGGGGCCGGGGCCGGGGCCGGGGCCGGGGCCGGGGCCGGGGCCGGGGCCGGGGCCGGGGCCGGGRCCGGGGCCGGGGCCGGGGCCGGGGCCGGGGCCGGGGCCGGGGCCGGGGCCGGGGCCGGGGCCGGGGCCGGGGCYGGGGCCGGGGCCGGGGCCGGGGCCGGGGCCGGGGCCGGGGCCGGGGCCGGGGCCGGGGCCGGGGCCGGGGCCGGGGCCGGGGCCGGGGCCGGGGCCGGGGCCGGGGCCGGGGCCGGGGCCGGGGCCGGGGCCGGGGCCGGGGCCGGGGCCGGGGCCGGGGCCGGGGCCGGGGCCGGGGCCGGGGCCGGGGCCGGGGCCGGGGCCGGGGCCGGGGCCGGGGCCGGGGCCGGGGCCGGGGCCGGGGCCGGGGCCGGGGCCGGGGCCGGGGCCGGGGCCGGGGCCGGGGCCGGGGCCGGGGCCGGGGCCGGGGCCGGGGCCGGGGCCGGGGCCGGGGCCGGGGCCGGGGCCGGGGCCGGGGCCGGGGCCGGGGCCGGGGCCGGGGCCGGGGCCGGGGCCGGGGCCGGGGCCGGGGCCGGGGCCGGGGCCGGGGCCGGGGCCGGGGCCGGGGCCGGGGCCGGGGCCGGGGCCGGGGCCGGGGCCGGGGCCGGGGCCGGGGCCGGGGCCGGGGCCGGGGCCGGGGCCGGGGCCGGGGCCGGGGCCGGGGCCGGGGCCGGGGCCGGGGCCGGGGCCGGGGCCGGGGCCGGGGCCGGGGCCGGGGCCGGGGCCGGGGCCGGGGCCGGGGCCGGGGCCGGGGCCGGGGCCGGGGCCGGGGCCGGGGCCGGGGCCGGGGCCGGGGCCGGGGCCGGGGCCGGGGCCGGGGCCGGGGCCGGGGCCGGGGCCGGGGCCGGGGCCGGGGCCGGGGCCGGGGCCGGGGCCGGGGCCGGGGCCGGGGCCGGGGCCGGGGCCGGGGCCGGGGCCGGGGCCGGGGCCGGGGCCGGGRCCGGGGCCGGGGCCGGGGCCGGGGCCGGGGCCGGGGCCGGGGCCGGGGCCGGGGCCGGGGCCGGGGCCGGGGCYGGGGCCGGGGCCGGGGCCGGGGCCGGGGCCGGGGCCGGGGCCGGGGCCGGGGCCGGGGCCGGGGCCGGGGCCGGGGCCGGGGCCGGGGCCGGGGCCGGGGCCGGGGCCGGGGCCGGGGCCGGGGCCGGGGCCGGGGCCGGGGCCGGGGCCGGGGCCGGGGCCGGGGCCGGGGCCGGGGCCGGGGCCGGGGCCGGGGCCGGGGCCGGGGCCGGGGCCGGGGCCGGGGCCGGGGCCGGGGCCGGGGCCGGGGCCGGGGCCGGGGCCGGGGCCGGGGCCGGGGCCGGGGCCGGGGCCGGGGCCGGGGCCGGGGCCGGGGCCGGGGCCGGGGCCGGGGCCGGGGCCGGGGCCGGGGCCGGGGCCGGGGCCGGGGCCGGGGCCGGGGCCGGGGCCGGGGCCGGGGCCGGGGCCGGGGCCGGGGCCGGGGCCGGGGCCGGGGCCGGGGCCGGGGCCGGGGCCGGGGCCGGGGCCGGGGCCGGGGCCGGGGCCGGGGCCGGGGCCGGGGCCGGGGCCGGGGCCGGGGCCGGGGCCGGGGCCGGGGCCGGGGCCGGGGCCGGGGCCGGGGCCGGGGCCGGGGCCGGGGCCGGGGCCGGGGCCGGGGCCGGGGCCGGGGCCGGGGCCGGGGCCGGGGCCGGGGCCGGGGCCGGGGCCGGGGCCGGGGCCGGGGCCGGGGCCGGGGCCGGGGCCGGGGCCGGGGCCGGGGCCGGGGCCGGGGCCGGGGCCGGGGCCGGGGCCGGGGCCGGGGCCGGGGCCGGGGCCGGGGCCGGGGCCGGGGCCGGGGCCGGGGCCGGGGCCGGGGCCGGGGCCGGGGCCGGGGCCGGGGCCGGGGCCGGGGCCGGGGCCGGGGCCGGGGCCGGGGCCGGGGCCGGGGCCGGGGCCGGGGCCGGGGCCGGGGCCGGGGCCGGGGCCGGGGCCGGGGCCGGGGCCGGGGCCGGGGCCGGGGCCGGGGCCGGGGCCGGGGCCGGGGCCGGGGCCGGGGCCGGGGCCGGGGCCGGGGCCGGGGCCGGGGCCGGGGCCGGGGCCGGGGCCGGGGCCGGGGCCGGGGCCGGGGCCGGGGCCGGGGCCGGGGCCGGGGCCGGGGCCGGGGCCGGGGCCGGGGCCGGGGCCGGGGCCGGGGCCGGGGCCGGGGCCGGGGCCGGGRCCGGGGCCGGGGCCGGGGCCGGGGCCGGGGCCGGGGCCGGGGCCGGGGCCGGGGCCGGGGCCGGGGCCGGGGCYGGGGCCGGGGCCGGGGCCGGGGCCGGGGCCGGGGCCGGGGCCGGGGCCGGGGCCGGGGCCGGGGCCGGGGCCGGGGCCGGGGCCGGGGCCGGGGCCGGGGCCGGGGCCGGGGCCGGGGCCGGGGCCGGGGCCGGGGCCGGGGCCGGGGCCGGGGCCGGGGCCGGGGCCGGGGCCGGGGCCGGGGCCGGGGCCGGGGCCGGGGCCGGGGCCGGGGCCGGGGCCGGGGCCGGGGCCGGGGCCGGGGCCGGGGCCGGGGCCGGGGCCGGGGCCGGGGCCGGGGCCGGGGCCGGGGCCGGGGCCGGGGCCGGGGCCGGGGCCGGGGCCGGGGCCGGGGCCGGGGCCGGGGCCGGGGCCGGGGCCGGGGCCGGGGCCGGGGCCGGGGCCGGGGCCGGGGCCGGGGCCGGGGCCGGGGCCGGGGCCGGGGCCGGGGCCGGGGCCGGGGCCGGGGCCGGGGCCGGGGCCGGGGCCGGGGCCGGGGCCGGGGCCGGGGCCGGGGCCGGGGCCGGGGCCGGGGCCGGGGCCGGGGCCGGGGCCGGGGCCGGGGCCGGGGCCGGGGCCGGGGCCGGGGCCGGGGCCGGGGCCGGGGCCGGGGCCGGGGCCGGGGCCGGGGCCGGGGCCGGGGCCGGGGCCGGGGCCGGGGCCGGGGCCGGGGCCGGGGCCGGGGCCGGGGCCGGGGCCGGGGCCGGGGCCGGGGCCGGGGCCGGGGCCGGGGCCGGGGCCGGGGCCGGGGCCGGGGCCGGGGCCGGGGCCGGGGCCGGGGCCGGGGCCGGGGCCGGGGCCGGGGCCGGGGCCGGGGCCGGGGCCGGGGCCGGGGCCGGGRCCGGGGCCGGGGCCGGGGCCGGGGCCGGGGCCGGGGCCGGGGCCGGGGCCGGGGCCGGGGCCGGGGCCGGGGCYGGGGCCGGGGCCGGGGCCGGGGCCGGGGCCGGGGCCGGGGCCGGGGCCGGGGCCGGGGCCGGGGCCGGGGCCGGGGCCGGGGCCGGGGCCGGGGCCGGGGCCGGGGCCGGGGCCGGGGCCGGGGCCGGGGCCGGGGCCGGGGCCGGGGCCGGGGCCGGGGCCGGGGCCGGGGCCGGGGCCGGGGCCGGGGCCGGGGCCGGGGCCGGGGCCGGGGCCGGGGCCGGGGCCGGGGCCGGGGCCGGGGCCGGGGCCGGGGCCGGGGCCGGGGCCGGGGCCGGGGCCGGGGCCGGGGCCGGGGCCGGGGCCGGGGCCGGGGCCGGGGCCGGGGCCGGGGCCGGGGCCGGGGCCGGGGCCGGGGCCGGGGCCGGGGCCGGGGCCGGGGCCGGGGCCGGGGCCGGGGCCGGGGCCGGGGCCGGGGCCGGGGCCGGGGCCGGGGCCGGGGCCGGGGCCGGGGCCGGGGCCGGGGCCGGGGCCGGGGCCGGGGCCGGGGCCGGGGCCGGGGCCGGGGCCGGGGCCGGGGCCGGGGCCGGGGCCGGGGCCGGGGCCGGGGCCGGGGCCGGGGCCGGGGCCGGGGCCGGGGCCGGGGCCGGGGCGTGGTCGGGGCGGGCCCGGGGGCGGGCCCGGGGCGGGGCTGCGGTTGCGGTGCCTGCGCCCGCGGCGGCGGAGGCGCAGGCGGTGGCGAGTGGGGGATCCGCGGCCGCGATTACAAGGACGACGACGACAAGATACCCATACGACGTTCCAGATTACGCTAGAACAAAAACTTATTTCTGAAGAAGATCTGCATCATCATCATCATCATTAGCTCGAGTCTAGAGGGCCCTTCGAAGGTAAGCCTATCCCTAACCCTCTCCTCGGTCTCGATTCTACGCGTACCGGTCATCATCACCATCACCATTGAGTTTAAACCCGCTGATCAGCCTCGACTGTGCCTTCTAGTTGCCAGCCATCTGTTGTTTGCCCCTCCCCCGTGCCTTCCTTGACCCTGGAAGGTGCCACTCCCACTGTCCTTTCCTAATAAAATGAGGAAATTGCATCGCATTGTCTGAGTAGGTGTCATTCTATTCTGGGGGGTGGGGTGGGGCAGGACAGCAAGGGGGAGGATTGGGAAGACAATAGCAGGCATGCTGGGGATGCGGTGGGCTCTATGGCTTCTGAGGCGGAAAGAACCAGCTGGGGCTCTAGGGGGTATCCCCACGCGCCCTGTAGCGGCGCATTAAGCGCGGCGGGTGTGGTGGTTACGCGCAGCGTGACCGCTACACTTGCCAGCGCCCTAGCGCCCGCTCCTTTCGCTTTCTTCCCTTCCTTTCTCGCCACGTTCGCCGGCTTTCCCCGTCAAGCTCTAAATCGGGGGCTCCCTTTAGGGTTCCGATTTAGTGCTTTACGGCACCTCGACCCCAAAAAACTTGATTAGGGTGATGGTTCACGTAGTGGGCCATCGCCCTGATAGACGGTTTTTCGCCCTTTGACGTTGGAGTCCACGTTCTTTAATAGTGGACTCTTGTTCCAAACTGGAACAACACTCAACCCTATCTCGGTCTATTCTTTTGATTTATAAGGGATTTTGCCGATTTCGGCCTATTGGTTAAAAAATGAGCTGATTTAACAAAAATTTAACGCGAATTAATTCTGTGGAATGTGTGTCAGTTAGGGTGTGGAAAGTCCCCAGGCTCCCCAGCAGGCAGAAGTATGCAAAGCATGCATCTCAATTAGTCAGCAACCAGGTGTGGAAAGTCCCCAGGCTCCCCAGCAGGCAGAAGTATGCAAAGCATGCATCTCAATTAGTCAGCAACCATAGTCCCGCCCCTAACTCCGCCCATCCCGCCCCTAACTCCGCCCAGTTCCGCCCATTCTCCGCCCCATGGCTGACTAATTTTTTTTATTTATGCAGAGGCCGAGGCCGCCTCTGCCTCTGAGCTATTCCAGAAGTAGTGAGGAGGCTTTTTTGGAGGC

**Supplemental data 2. MinION consensus sequence in the C9-774 repeat region is 26.55% accurate, when compared to the plasmid reference sequence.** We aligned the MinION consensus sequence for just the repeat region to the plasmid’s reference sequence. The sequences were 26.55% similar. Many guanines and cytosines were erroneously identified as adenine. Thus, in the MinION consensus sequence, guanines and cytocines were represented as mixed nucleotides in the consensus (e.g., R representing G or A, and M representing C or A). Exactly 553 (71.4%) of the 774 MinION repeats were represented as either RRRRCM or RRRRMC. We include the entire C9-774 MinION consensus sequence. Defined repeat region highlighted in yellow.

>C9-774_MinION_consensus_sequence CTAGGCTTTTGCAAAAAGCTCCCGGGAGCTTGTATATCCATTTTCGGATCTGATCAGCACGTGTTGACAATTAATCATCGGCATAGTATATCGGCATAGTATAATACGACAAGGTGAGGAACTAAACCATGGCCAAGCCTTTGTCTCAAGAAGAATCCACCCTCATTGAAAGAGCAACGGCTACAATCAACAGCATCCCCATCTCTGAAGACTACAGCGTCGCCAGCGCAGCTCTCTCTAGCGACGGCCGCATCTTCACTGGTGTCAATGTATATCATTTTACTGGGGGACCTTGTGCAGAACTCGTGGTGCTGGGCACTGCTGCTGCTGCGGCAGCTGGCAACCTGACTTGTATCGTCGCGATCGGAAATGAGAACAGGGGCATCTTGAGCCCCTGCGGACGGTGCCGACAGGTGCTTCTCGATCTGCATCCWGGGATCAAAGCCATAGTGAAGGACAGTGATGGACAGCCGACGGCAGTTGGGATTCGTGAATTGCTGCCCTCTGGTTATGTGTGGGAGGGCTAAGCACTTCGTGGCCGAGGAGCAGGACTGACACGTGCTACGAGATTTCGATTCCACCGCCGCCTTCTATGAAAGGTTGGGCTTCGGAATCGTTTTCCGGGACGCCGGCTGGATGATCCTCCAGCGCGGGGATCTCATGCTGGAGTTCTTCGCCCACCCCAACTTGTTTATTGCAGCTTATAATGGTTACAAATAAAGCAATAGCATCACAAATTTCACAAATAAAGCATTTTTTTCACTGCATTCTAGTTGTGGTTTGTCCAAACTCATCAATGTATCTTATCATGTCTGTATACCGTCGACCTCTAGCTAGAGCTTGGCGTAATCATGGTCATAGCTGTTTCCTGTGTGAAATTGTTATCCGCTCACAATTCCACACAACATACGAGCCGGAAGCATAAAGTGTAAAGCCWGGGGTGCCTAATGAGTGAGCTAACTCACATTAATTGCGTTGCGCTCACTGCCCGCTTTCCAGTCGGGAAACCTGTCGTGCCAGCTGCATTAATGAATCGGCCAACGCGCGGGGAGAGGCGGTTTGCGTATTGGGCGCTCTTCCGCTTCCTCGCTCACTGACTCGCTGCGCTCGGTCGTTCGGCTGCGGCGAGCGGTATCAGCTCACTCAAAGGCGGTAATACGGTTATCCACAGAATCAGGGGATAACGCAGGAAAGAACATGTGAGCAAAAGGCCAGCAAAAGGCSRGGAACCGTAAAAAGGCCGCGTTGCTGGCGTTTTTCCATAGGCTCCGCCCCCCTGACGAGCATCACAAAAATCGACGCTCAAGTCAGAGGTGGCGAAACCCGACAGGACTATAAAGATACCRGGCGTTTCCCCCWGGAAGCTCCCTCGTGCGCTCTCCTGTTCCGACCCTGCCGCTTACCGGATACCTGTCCGCCTTTCTCCCTTCGGGAAGCGTGGCGCTTTCTCATAGCTCACGCTGTAGGTATCTCAGTTCGGTGTAGGTCGTTCGCTCCAAGCTGGGCTGTGTGCACGAACCCCCCGTTCAGCCCGACCGCTGCGCCTTATCCGGTAACTATCGTCTTGAGTCCAACCCGGTAAGACACGACTTATCGCCACTGGCAGCAGCCACTGGTAACAGGATTAGCAGAGCGAGGTATGTAGGCGGTGCTACAGAGTTCTTGAAGTGGTGGCCTAACTACGGCTACACTAGAAGAACAGTATTTGGTATCTGCGCTCTGCTGAAGCCAGTTACCTTCGGAAAAAGAGTTGGTAGCTCTTGATCCGGCAAACAAACCACCGCTGGTAGCGGTGGTTTTTTTGTTTGCAAGCAGCAGATTACGCGCAGAAAAAAAGGATCTCAAGAAGATCCTTTGATCTTTTCTACGGGGTCTGACGCTCAGTGGAACGAAAACTCACGTTAAGGGATTTTGGTCATGAGATTATCAAAAAGGATCTTCACCTAGATCCTTTTAAATTAAAAATGAAGTTTTAAATCAATCTAAAGTATATATGAGTAAACTTGGTCTGACAGTTACCAATGCTTAATCAGTGAGGCACCTATCTCAGCGATCTGTCTATTTCGTTCATCCATAGTTGCCTGACTCCCCGTCGTGTAGATAACTACGATACGGGAGGGCTTACCATCTGGCCCCAGTGCTGCAATGATACCGCGAGACCCACGCTCACCGGCTCCAGATTTATCAGCAATAAACCAGCCAGCCGGAAGGGCCGAGCGCAGAAGTGGTCCTGCAACTTTATCCGCCTCCATCCAGTCTATTAATTGTTGCCGGGAAGCTAGAGTAAGTAGTTCGCCAGTTAATAGTTTGCGCAACGTTGTTGCCATTGCTACAGGCATCGTGGTGTCACGCTCGTCGTTTGGTATGGCTTCATTCAGCTCCGGTTCCCAACGATCAAGGCGAGTTACRYGATCCCCCATGTTGTGCAAAAAAGCGGTTAGCTCCTTCGGTCCTCCGATCGTTGTCAGAAGTAAGTTGGCCGCAGTGTTATCACTCATGGTTATGGCAGCACTGCATAATTCTCTTACTGTCATGCCATCCGTAAGATGCTTTTCTGTGACTGGTGAGTACTCAACCAAGTCATTCTGAGAATAGTGTATGCGGCGACCGAGTTGCTCTTGCCCGGCGTCAATACGGGATAATACCGCGCCACATAGCAGAACTTTAAAAGTGCTCATCATTGGAAAACGTTCTTCGGGGCGAAAACTCTCAAGGATCTTACCGCTGTTGAGATCCAGTTCGATGTAACCCACTCGTGCACCCAACTGATCTTCAGCATCTTTTACTTTCACCAGCGTTTCTGGGTGAGCAAAAACAGGAAGGCAAAATGCCGCAAAAAAGGGAATAAGGGCGACACGGAAATGTTGAATACTCATACTCTTCCTTTTTCAATATTATTGAAGCATTTATCAGGGTTATTGTCTCATGAGCGGATACATATTTGAATGTATTTAGAAAAATAAACAAATAGGGGTTCCGCGCACATTTCCCCGAAAAGTGCCACCTGACGTCGACGGATCGGGAGATCTCCCGATCCCCTATGGTGCACTCTCAGTACAATCTGCTCTGATGCCGCATAGTTAAGCCAGTATCTGCTCCCTGCTTGTGTGTTGGAGGTCGCTGAGTAGTGCGCGAGCAAAATTTAAGCTACAACAAGGCAAGGCTTGACCGACAATTGCATGAAGAATCTGCTTAGGGTTAGGCGTTTTGCGCTGCTTCGCGATGTACGGGCCAGATATACGCGTTGACATTGATTATTGACTAGTYGAGKCCCTTTCGTCTTCACTCGAGTTTACTCCCTATCAGTGATAGAGAACGTATGTCGAGTTTACTCCCTATCAGTGATAGAGAACGATGTCGAGTTTACTCCCTATCAGTGATAGAGAACGTATGTCGAGTTTACTCCCTATCAGTGATAGAGAACGTATGTCGAGTTTACTCCCTATCAGTGATAGAGAACGTATGTCGAGTTTATCCCTATCAGTGATAGAGAACGTATGTCGAGTTTACTCCCTATCAGTGATAGAGAACGTATGTCGAGGTAGGCGTGTACGGTGGGAGGCCTATATAAGCAGAGCTCGTTTAGTGAACCGTCAGATCGCCAAGCTTAGTACTCGCTGAGGGTGAACAAGAAAAGACCTGATAAAGATTAACCAGAAGAAAACAAGGAGGGAAACAACCGCAGCCTGTAGCAAGCTCTGGAACTCAGGAGTCGCGCGCTAGGGGCCGGGGCCGGGGCCGGGGCCGGGGCCGGGGCCGGGGCCGGGGCCGGGGCCGGGGCCGGGGCCGGGGCCGGGGCCGGGGCCRGGACCGGGGCCRGGGCCRGGGCCRGGRCCRGGRCCRGGRCCRGGRCCRGGRCCRGGGCCRGGGCCRGGGCCRGGGCTGRGGCMRGRRCCRGRRCCRGRRCCRGRRCCRGRRCCRGRRCCRGRRCCRGRRCCRGRRCCRGRRCCRGRRCCRGRRCCRGRRCCRGRRCCRGRRCCRGRRCCRGRRCCRRRRCCRGRRCCRRRRCMRRRRCCRRRRCMRRRRCMRRRRCMRRRRCMRRRRCMRRRRCMRRRRCMRRRRCMRRRRCMRRRRCMRRRRCMRRRRCMRRRRCMRRRRCMRRRRCMRRRRCMRRRRCMRRRRCMRRRRCMRRRRCMRRRRCMRRRRCMRRRRCMRRRRCMRRRRCMRRRRCMRRRRCMRRRRCMRRRRCMRRRRCMRRRRCMRRRRCMRRRRCMRRRRCMRRRRCMRRRRCMRRRRCMRRRRCMRRRRCMRRRRCMRRRRCMRRRRCMRRRRCMRRRRCMRRRRCMRRRRCMRRRRCMRRRRCMRRRRCMRRRRCMRRRRCMRRRRCMRRRRCMRRRRCMRRRRCMRRRRCMRRRRCMRRRRCMRRRRCMRRRRCMRRRRCMRRRRCMRRRRCMRRRRCMRRRRCMRRRRCMRRRRCMRRRRCMRRRRCMRRRRCMRRRRCMRRRRCMRRRRCMRRRRCMRRRRCMRRRRCMRRRRCMRRRRCMRRRRCMRRRRCMRRRRCMRRRRCMRRRRCMRRRRCMRRRRCMRRRRCMRRRRCMRRRRCMRRRRCMRRRRCMRRRRCMRRRRCMRRRRCMRRRRCMRRRRCMRRRRCMRRRRCMRRRRCMRRRRCMRRRRCMRRRRCMRRRRCMRRRRCMRRRRCCRRRRCMRRRRCMRRRRCCRRRRCCRRRRCCRRRRCMRRRRCCRRRRCCRRRRCCRRRRCCRRRAMCGGGRCCRRRRCCRRRRCCRRRRCCRRRRCCRRRRCCRRRRCCRRRRCCRRRRCCRRRRCCRRRRNCRGGRMTGRRRCMRRRRCMRRRRCMRRRRCCRRRRCCRRRRCCRRRRCMRRRRCCRRRRCCRRRRCMRRRRCCRRRRCCRRRRCCRRRRCMRRRRCCRRRRCMRRRRCMRRRRCMRRRRCMRRRRCCRRRRCMRRRRCMRRRRCMRRRRCMRRRRCMRRRRCCRRRRCCRRRRCMRRRRCMRRRRCMRRRRCMRRRRCMRRRRCMRRRRCMRRRRCCRRRRCCRRRRCCRRRRCCRRRRCCRRRRCCRRRRCMRRRRCMRRRRCMRRRRCCRRRRCMRRRRCCRRRRCCRRRRCCRRRRCCRRRRCCRRRRCCRRRRCCRRRRCCRRRRCCRRRRCCRRRRMCRRRRCCRRRRCCRRRRCCRRRRMCRRRRCCRRRRMMRRRRMCRRRRMCRRRRMCRRRRMCRRRRMCRRRRMCRRRRMCRRRRMCRRRRMCRRRRMCRRRRMCRRRRMCRRRRMCRRRRMCRRRRMCRRRRMCRRRRMCRRRRMCRRRRMCRRRRMCRRRRMCRRRRMCRRRRMCRRRRMCRRRRMCRRRRMCRRRRMCRRRRMCRRRRMCRRRRMCRRRRMCRRRRMCRRRRMCRRRRMCRRRRMCRRRRMCRRRRMCRRRRMCRRRRMCRRRRMCRRRRMCRRRRMCRRRRMCRRRRMCRRRRMCRRRRMCRRRRMCRRRRMCRRRRMCRRRRMCRRRRMCRRRRMCRRRRMCRRRRMCRRRRMCRRRRMCRRRRMCRRRRMCRRRRMCRRRRMCRRRRNCRRRRNCRRRRMCRRRRMCRRRRNCRRRRMCRRRRMCRRRRNCRRRRNCRRRRNCRRRRMCRRRRMCRRRRMCRRGRNCRRNAMCGGGRCCRRRRMCRRRRNCRRRRNCRRRRMCRRRRMCRRRRMCRRRRMCRRRRMCRRRRNCRRRRNCRGGRMTGRRRCMRRRRCMRRRRCMRRRRCCRRRRMCRRRRMCRRRRMCRRRRMCRRRRMCRRRRMCRRRRMCRRRRMCRRRRMCRRRRMCRRRRMCRRRRMCRRRRMCRRRRMCRRRRMCRRRRNCRRRRNCRRRRNCRRRRMCRRRRNCRRRRNCRRRRNCRRRAMCRRRRNCRRRRNCRRRRNCRRRRNCRRRRNCRRRRNCRRRANCRRRRMCRRRRNCRRRRNCRRRRNCRRRRNCRRRRNCRRRRNCRRRRNCRRRRNCRRRRNCRRRRNCRRRRNCRRRRNCRRRRNCRRRRNCRRRRNCRRRRNCRRRRNCRRRRNCRRRRNCRRRRMCRRRRNCRRRRMCRRRRMCRRRRMCRRRRMCRRRRMCRRRRCMRRRRCMRRRRCMRRRRCMRRRRCMRRRRCMRRRRCMRRRRCMRRRRCMRRRRCMRRRRCMRRRRCMRRRRCMRRRRCMRRRRCMRRRRCMRRRRCMRRRRCMRRRRCMRRRRCMRRRRCMRRRRCMRRRRCMRRRRCMRRRRCMRRRRCMRRRRCMRRRRCMRRRRCMRRRRCMRRRRCMRRRRCMRRRRCMRRRRCMRRRRCMRRRRCMRRRRCMRRRRCMRRRRCMRRRRCMRRRRCMRRRRCMRRRRCMRRRRCMRRRRCMRRRRCMRRRRCMRRRRCMRRRRCMRRRRCMRRRRCMRRRRCMRRRRCMRRRRCMRRRRCMRRRRCMRRRRCMRRRRCMRRRRCMRRRRCMRRRRCMRRRRCMRRRRCMRRRRCMRRRRCMRRRRCMRRRRCMRRRRCMRRRRCMRRRRCMRRRRCMRRRRCMRRRRCMRRRRCMRRRRCMRRRRCMRRRRCMRRRRCMRRRRCMRRRRCMRRRRCMRRRRCMRRRRCMRRRRCMRRRRCMRRRRCMRRRRCMRRRRCMRRRRCMRRRRCMRRRRCMRRRRCMRRRRCMRRRRCMRRRRCMRRRRCMRRRRCMRRRRCMRRRRCMRRRRCMRRRRCMRRRRCMRRRRCMRRRRCMRRRRCMRRRRCMRRRRCMRRRRCMRRRRCMRRRRCMRRRRCMRRRRCMRRRRCMRRRRCMRRRRCMRRRRCMRRRRCMRRRRCMRRRRCMRRRRCMRRRRCMRRRRCMRRRRCMRRRRCMRRRRCMRRRRCMRRRRCMRRRRCMRRGACMRRRRCMRRRRCMRRRRCMRRRRCMRRRRCMRRRRCMRRRRCMRRRRCMRRRRCMRRRRCMRRRRCMRGGRCTGRRRCARRRRCMRRRRCMRRRRCMRRRRCMRRRRCMRRRRCMRRRRCMRRRRCMRRRRCMRRRRCMRRRRCMRRRRCMRRRRCMRRRRCMRRRRCMRRRRCMRRRRCMRRRRCMRRRRCMRRRRCMRRRRCMRRRRCMRRRRCMRRRRCMRRRRCMRRRRCMRRRRCMRRRRCMRRRRCMRRRRCMRRRRCMRRRRCMRRRRCMRRRRCMRRRRCMRRRRCMRRRRCMRRRRCMRRRRCMRRRRCMRRRRCMRRRRCMRRRRCMRRRRCMRRRRCMRRRRCMRRRRCMRRRRCMRRRRCMRRRRCMRRRRCMRRRRCMRRRRCMRRRRCMRRRRCMRRRRCMRRRRCMRRRRCMRRRRCMRRRRCMRRRRCMRRRRCMRRRRCMRRRRCMRRRRCMRRRRCMRRRRCMRRRRCMRRRRCMRRRRCMRRRRCMRRRRCMRRRRCMRRRRCMRRRRCMRRRRCMRRRRCMRRRRCMRRRRCMRRRRCMRRRRCMRRRRCMRRRRCMRRRRCMRRRRCMRRRRCMRRRRCMRRRRCMRRRRCMRRRRCMRRRRCMRRRRCMRRRRCMRRRRCMRRRRCMRRRRCMRRRRCMRRRRCMRRRRCMRRRRCMRRRRCMRRRRCMRRRRCMRRRRCMRRRRCMRRRRCMRRRRCMRRRRCMRRRRCMRRRRCMRRRRCMRRRRCMRRRRCMRRRRCMRRRRCMRRRRCMRRRRCMRRRRCMRRRRCMRRRRCMRRRRCMRRRRCMRRRRCMRRRRCMRRRRCMRRRRCMRRRRCMRRRRCMRRRRCMRRRRCMRRRRCMRRRRCMRRRRCMRGRRCMRRRRCMRGRACMRRRRCMRRRRCMRRRRCMRRRRCMRRRRCMRRRRCMRRRRCMRRRRCMRRRRCMRRRRCMRRRRCMRGGRCTGRRRCMRRRRCMRRRRCMRRRRCMRRRRCMRRRRCMRRRRCMRRRRCMRRRRCMRRRRCMRRRRCMRRRRCMRRRRCMRRRRCMRRRRCMRRRRCMRRRRCMRRRRCMRRRRCMRRRRCMRRRRCMRRRRCMRRRRCMRRRRCMRRRRCMRRRRCMRRRRCMRRRRCMRRRRCMRRRRCMRRRRCMRRRRCMRRRRCMRRRRCMRRRRCMRRRRCMRGRRCMRRRRCMRRRRCMRGRRCMRGRRCMRGRRCCRGRRCCRGRRCMRGRRCCRGRRCCRGRRCCRGRRCCRGRRCCRGRRCCRGRRCCRGRRCCRGRRCCRGRRCCRGRRCCRGRRCCRGGRCCRGGRCCRGGRCCRGGRCCRGGRCCRGGRCCRGGRCCRGGRCCRGGRCCRGGRCCRGGRCCGGGGCCGGGGCCGGGGCCGGGGCCGGGGCCGGGGCCGGGGCCGGGGCCGGGGCCGGGGCCGGGGCCGGGGCCGGGGCCGGGGCCGGGGCCGGGGCCGGGGCCGGGGCCGGGGCCGGGGCCGGGGCCGGGGCCGGGGCCGGGGCCGGGGCCGGGGCCGGGGCCGGGGCCGGGGCCGGGGCCGGGGCCGGGGCGTGGTCGGGGCRGGCCCGGGGGCRGGCCCGGGGCRGGGCTGCGGTTGCRGTGCCTGCGCCCGCGGCGGCGGAGGCGCAGGCGGTGGCGAGTGGGGGATCCGCGGCCGCGATTACAAGGACGACGACGACAAGATACCCATACGACGTTCCAGATTACGCTAGAACAAAAACTTATTTCTGAAGAAGATCTGCATCATCATCATCATCATTAGCTCGAGTCTAGAGGGCCCTTCGAAGGTAAGCCTATCCCTAACCCTCTCCTCGGTCTCGATTCTACGCGTACCGGTCATCATCACCATCACCATTGAGTTTAAACCCGCTGATCAGCCTCGACTGTGCCTTCTAGTTGCCAGCCATCTGTTGTTTGCCCCTCCCCCGTGCCTTCCTTGACCCTGGAAGGTGCCACTCCCACTGTCCTTTCCTAATAAAATGAGGAAATTGCATCGCATTGTCTGAGTAGGTGTCATTCTATTCTGGGGGGTGGGGTGGGGCAGGACAGCAAGGGGGAGGATTGGGAAGACAATAGCAGGCATGCTGGGGATGCGGTGGGCTCTATGGCTTCTGAGGCGGAAAGAACCAGCTGGGGCTCTAGGGGGTATCCCCACGCGCCCTGTAGCGGCGCATTAAGCGCGGCRGGTGTGGTGGTTACGCGCAGCGTGACCGCTACACTTGCCAGCGCCCTAGCGCCCGCTCCTTTCGCTTTCTTCCCTTCCTTTCTCGCCACGTTCGCCGGCTTTCCCCGTCAAGCTCTAAATCRGGGGCTCCCTTTAGGGTTCCGATTTAGTGCTTTACRGCACCTCGACCCCAAAAAACTTGATTAGGGTGATGGTTCACGTAGTGGGCCATCGCCCTGATAGACRGTTTTTCGCCCTTTGACGTTGGAGTCCACGTTCTTTAATAGTGGACTCTTGTTCCAAACTGGAACAACACTCAACCCTATCTCGGTCTATTCTTTTGATTTATAAGGGATTTTGCCGATTTCGGCCTATTGGTTAAAAAATGAGCTGATTTAACAAAAATTTAACGCGAATTAATTCTGTGGAATGTGTGTCAGTTAGGGTGTGGAAAGTCCCSAGGCTCCCCAGCAGGCAGAAGTATGCAAAGCATGCATCTCAATTAGTCAGCAACCAGGTGTGGAAAGTCCCSAGGCTCCCCAGCAGGCAGAAGTATGCAAAGCATGCATCTCAATTAGTCAGCAACCATAGTCCCGCCCCTAACTCCGCCCATCCCGCCCCTAACTCCGCCCAGTTCCGCCCATTCTCCGCCCCATGGCTGACTAATTTTTTTTATTTATGCAGAGGCCGAGGCCGCCTCTGCCTCTGAGCTATTCCAGAAGTAGTGAGGAGGCTTTTTTGGAGGC

**Supplemental data 3. MinION read covering the non-pathogenic allele.** Defined repeat region highlighted in yellow.

Read name = a1677441-358d-4dfa-9de9-c1fa5be4d9e5

Read length = 4,985bp

----------------------

Mapping = Primary @ MAPQ 40

Reference span = chr9:27,569,573-27,574,622 (+) = 5,050bp

Cigar = 2M2I13M1D32M1D27M1D5M1I1M1I10M...1D2M1I7M1I17M1D1M1D24M1D2M2D9M

Clipping = None

----------------------

Location = chr9:27,573,587

Base = C @ QV 22

----------------------

H0 = 1

ZE = 0.0

ZF = 0.384752

NM = 517

ZQ = 4985

ZR = 138394717

AS = 20040

-------------------

Alignment start position = chr9:27569573

ACATTGCTTCGTTTTATAAGGCTATACTATCTAGGTTTGTGTAAGTACATCTATGATGTTTGCATAATGACAAATTGCATACAATGATGCATTCTAATGTGTGATTGTGCTATAATTGAAGACTTGTTTATCTAAGACTGAAAGTAAAAAGAATTACAATTTCACCTAAGCAAGTCTAAAAAACTGTGAAATCTATTTATAATATGTAATACAAAAGCAGCTAATAGGCAAACTATGATATACCTATATCTTTTGCCATATGATTGCTTTGAGACTAACATTGATCTGTAAATGTATGACAAAGTAAACAATTTTACTTAAGAATTTCATCCACATCTTGTCAGAGAGGTTCAGTCTGTTGGAAAGCACTTGACTTCTATTTACAGAGCATTAGATGAGTGCTTTTATCATATTATGAGTAGGCATACAGGCCTGGCGAGCAGTTAACTCTAAGGTAATGTACAGAAATGGTTGAACACAGCGACAAGTTTTTCGCATTATTTGTAATTCAAAAAATTCATTTAGAGTATGTTACTTTTAAATATGTTGTATCAATTTAATAGTCTTAGAGACAGCACTGGATATAAGCGTACAGCTTCTTTAAAATGTCCACTGTTTTACTAATACAATGTAAGCCAGTCAGTTTACAATGATCAAATATAGAATGTAATCTGAATTGAAATAGTATGACACTACTGCTGTCATAATAACAACAACAAACTGGAGGCCAACATAATGAATTAAGTTAACATACAACAACAAAAATTATATTGTAAACATATTTTTCTTTCATTCTTTTGGGTTAAAAGATTGGATAATCATAAAGGCAATATTACAACTCTAATATTTCATCATTAAACACAAAAATAAAAGTATTTCCTAAAACAGAACTGAACCCTGGAGCAAAATCTGTGTGAAGTTGAATTATGGGGAAACTTTTACCACGTTGTGAAAATTAGACTATTATACTGCTAATTTTACTCTCTAACGGAATAAGAAAAAAAAAAAATGGGCAGAACATGGTGGGTCACACCTGTTATCCAACTCTTTGGAGGCGGGCAGTGGATCACCTGGTCAGGAGCTCAAGACCAGCCTGGCCAGCATGGTGAAACCCACCTCTACTAAAAGAAAATACAAAATTGGCGGGGTGTGGTGGTGGACACCTGTAATCCCAGCTACTTAGGGAGGCTAAGGGCAGGAGAGATCTCTCCTTGAGACACGGAGGTGGCAGAGGTTGCAATGAGCTGAAGTGTTTTCACTGCACTCCCAGCCTGGTGACAGAGAGAGAGACTCTGCCTCAAGAAAAAAATAAACAAACAAACAAACAAACAAAAGAATAAGAAAATGAAGGACAAAGATCATACTGAATTGTGGTTTTAAATCCTACCAAAGAAATAGCCTGGGAAATGAAATGTCACAGAGAAGTATAATCAGGAGAGCTGTACAATTATTTTACTAATACTTGAAGTCGTACGTCTTTGGTGAGAAAAATCCATACATGCAAATGCAGCTGAAAAAATCAGCTCAAAACCAATAGTTGTTTATGTACCTATCTTACGTACATGTAGTGCTGTCTACTCAGAGTTACCAAACATTAGCCAGTCTTTTGAGAAACCAAGATTCAAGTGTAGGTGAGACAGTGGCTTGCTCACAGGGTTCATAGAGAGGTTTCCCAATACACTCTGGAAATAATCCATACATGCAGACATAAGTTACATTAATTAACATCTGCTAAAACTGTTATTAGAGTGCTAAGTTTGAGGTTTTTGCTTTTTTTCTTTAAACGTCTGTTAAAAAACAACCATCTGCTTCCCTGATTGGTATTTAGAAAGGTGGTTGGTCCCTGCTATTGTAGTGAAAATTCTACAATCATAAAGCCCTCACTTCTTGTTTTTTTAAGAGACAGAGACCTCGTTTTGTCATCAGGCTGGAATGCACTGGCAGGATCATAGCTCTTACGGTAACTTCAAACTCTTGGGCTCCAAACCCTCCTGCTCAGCTCCCAAGTAGCTAGGACTACAGGTGCACATCACCGCCCGTGGGATTTTTAATTTTTTGTAGAGACAGGGTCTACGTTGCCCAGGTTGAGCTTGAACTCCCTGGCTTCAAGTAGATCCTCTTGCCTCCGCCTCCCAAAGCTCTGGCATTACAGGTGTAAGCCACCTTCTCCAACCTGGCTCTCAATACTTGTAACCATGCTGTTATTTTCTCCCAGCCCAAAGAGAAGCAGGATCCTAAACCGTCACTTTCCACAACAGGAGCTGCCCAGGACCACTTCAAGGACAGTGGACATTTTACAGTACCAGAAAGTTCACAACACTTTCTCAATCTTCAACATCAGGGAAGACTGGAAGGTGAAGTTCATATCACTATCTGGCCATTTCTCACAGTTCCAAGTTTCTCAGACAATGAGTAGGCTAACCTAGTCCTCCTGGGAACTATCTAATTAACGTAGAATAGAACCCGAGGGCAGACTTGAAAACAAGTCCTCCTTGGTTACTTTGTTTCTCTGAAACAAATTGTGGAGTGCCAACATAGCCAAACAAAATATTTTATCAACTTCATAAGGTGCTTGTAATTTTTCCTGGAGCAGGTAAATGCTGGCTTAGTGAACAATCTGGAATGTGGTAATTACTCGTTCTTGTTTTCAGATGTACTATCAGCATGTAGTTTCCAACTGATTCAGGGTTTTCCTAAAGTGGCAGGCCTTGGCAGAGGTGGTGACAACAATACTTGTGTCAAATGACACCGTATTTCAAGTATTCTGACTCAGGTTATTAATATCCCTATATGATAGTCTTGTTTCTGTGATATTCTGATTATGTTAAAGTTTCCCAAAGTCTGAGAAAATCATATCTGTAGTATCTTTTTTTTTTTTTGATCCTTTGTACAAAAGTAGAAGTAATACCAGACAGATACATTACCCTTGTTGTGAACAACTGGTGCATGGCAACTGTTTGAATGAAATTTACCAACTACCACAACCAGGCAACTACTCTCCCAGAGCCTAACAATCTCGATTGCATCTGAAAGACCACCCCTCCTGGGGAAAGTGCAGGACCTCCCTCCTGTTTTCTGAATACAAAATACCTGTTGGTGTTTTCAGCGCGTAGATGGACCCAATGAGCACACGGACATGTGATATTGCCTAACAGACAACTGATTACCATCCCAGAGTCAAGTGATGCCCAAGTCACAATGGTCCTTCCTTTAAGCAGAGTCTGTGTCATCTCTCCGGGCTGTGGAAGCAGCAGGTCATGTCCCTGGGAATGGGAGACACCGACTTGCATTGCTGCCCTCCTTAGCAAAGTCATCACCACTCTCTAGAAGCTTGGGCTGAAATTGTGCAGGCGTCTCACACCCCCCATCTCATCACATGATCTCCTCGCCGGCGGGGACCGTCTCAGGTTCCTGAACTGACTTGGTCACAGAAGCCGCGCGCCGCCACCCTCGGCCTTCCCCAGGCAGGGCCTCTCAGTGCGGGCTCCTTTCTCGAGCCCGCAGCGGCAGCGCTCCCAGCGGGTCCCGGGGGAAGGAGACGGCTCAGTGCTGAGGGCAGGCCTAAGGAAGAGGCCAGATCCATCCTTGTCCTGCGCCGCCGCCGCCGCCGCCGCCAGGAAGCCGGGGCGGATGCGAGCAATTCCACCGGTCGCTGGGGGCGAAGCCGACACCCAGCTTCGGTCGGAATGAGGAGGGAAAGTAAAAAATCGTCGGCTCTGGGAGGCCCCGCTTCTACCCGCGCCTCTTCCCAGCAGCCAGACCCCAAACAGCCACCCGCCAGGATGCCGCCTCCTCACTCACTTACTCGCCACCGCCGCCTCCGCCGCCGCGAACAGAACTCCTGGCACAGCCGCCCCGGGCCGCCCCGGGCCGCCGACTCCCCCCTTGGCCCCGGCCCGGCCCCAGCCCCGGCCCCGGCCCCGGCCCCGACTGGCGCGCGACTCCTGAGTTCAGAGCTTGCTACAGGCTGCGGTTGTTTCCTCCTTGTTTTCTTCTGGTTAAATCTTTATCGGGTCTTTCTTGTTCACCCTCAGCGAGTGCTGTGAGCAAGTAGTGGGGAGAGAGGTGGGAAAAACAAAACACACCTCCTAAACCCACCTGCTCACTGGGCCCGCCCCAAAGAAGTAACCAGACGCTTGGGGACGGCTGACACACCAAGCGTCATCTTTTACGTGGGCGGAACTTGTCCTTGTTTGACGCACCTCTCTTTCCCTAGCGGGACACCGTGGGACTACGTCTGTCTGTTTTCTATGTGCGATGACGTTTTCTCACGAGGCTAGCGAAATGGGGCGGGAGCAGCAATCCTGTTCTTTTATCTTAGACCCGCTCTGGAGGAGCGTTGGCGCAATAGCGTGTGAACCTTAATAGGGGAGGCTGCTTTGGATCGGAGAAAGTGAGACGTTTATTGGTTTTGAATGGTTTTGTTTGTGCTTGGTAGGCAGTGGGGCGCTCAACAAAATAATTGGTGGATGAAATTTTGTTTTACCGTAAGACACTGTTAGGAGTGCATTCAAAACTCCACTGCAAACCCTGGTGAGGACAGCTCCGGCACTGGGCGGGAATCCACGGTCCCCTGCAAAGTCATCTGTTTGCCTTTACATGTAAGAATTCTCTCAAGCATGATTTTCACGCAGGGAATGTCATTTTTTTGCTAGTTACAATATGTGGATAGAGTTGTTTTTTTTTTTAGCTTTTTTTTGAAAAACGTACCATTCTGTTTGATGTGTAAAAAACACAAAAGATTTTTGAAACCTTGCGTCTTTTTTGGTCTGCAGGTGTATAGGTCCACTTACTACAGATGGTAGCATTTACACCACTCAGATGTGTAAAAAAAAACAAGGTTTAAACTGGTAGCACAGATCTGCAAGTGTGAGATGGCACTTATTACAGTGAGTAGCATTTAATCTTTTCATCACTAAAATCCACACAGAGACGTTTTAATCATTCACGGGAAGAAAGGGAGGAATAAATACAAATGGCTCTCA

**Supplemental data 4. Sequel read covering the non-pathogenic allele.** Defined repeat region highlighted in yellow.

Read name = m54120_170621_235746/16188262/181_35079

Read length = 34,898bp

----------------------

Mapping = Primary @ MAPQ 40

Reference span = chr9:27,571,184-27,605,313 (-) = 34,130bp

Cigar = 1M1I14M1D2M1I20M1D4M1D13M1I14M...1D3M1I25M1D2M1I3M1D2M1D4M1I7M

Clipping = None

----------------------

Location = chr9:27,573,566

Base = T @ QV 0

----------------------

H0 = 1

ZE = 0.0

ZF = 0.471024

NM = 6243

ZQ = 34898

ZR = 138394717

AS = 110766

-------------------

Alignment start position = chr9:27571184

TGGAGGGAAGCCAAGATCAAAAATGAGTGAGACGGTGGCTGCTACAGGGTTCATGATGATGTTTCCCAATAACTTTATGGAATAATCCCAACATGCAGGAAAGATTACATTAATTAAACACATCTGCTAAAACTGTGTAGAGTGCGAAGTTTGAGGTTGCTTTTCTTTAAATCGTCTGTTAAAAATCAACCCATCTCTTCCCTGATTGGATTTAGAAAGGTGGTGGTCCATGCTATTGTCGTGAAAAATTTTACAATCAAAGCCCTCACTTCTTGTTTTAGAGCCCGGGTCTCGTTTTTGTCCTCAGGCGGAATGCACTGGCAGTATCATAGCTTCGGTAACTTCAAACTCCTGGGCTCAAATGAACCCCTGCATCAGCCTCCCAATGAGTCGGTGATACATGTGCTCCATCATCTCACGCTCCCTCCCCGGCTAAGTTTTTATATTTTTTGTAGAGAAGGGTTCTACGTGCACAGGGTGAGATTGATACTCCTGGCTTCAAGTGATCCTCTTGCCTCCGCGCCTCCCACAGCTCTGGCAACAGGTGTAAGCCACCTTCTCCACACCTGGCTCTCAATAATTGTAACCATGTGTTATTTCCTCCCCGCCCCACAAGAGAAGGGTCCTAAAAAACCGTCCACCTTTCCCATAAACAGGAGGACCCAGGGATCCACTTCAAGGACAGTGAAAACTGTTTTTACAGCAGTACCAGAAATTCACAACATTCTCAATCTTCAACATCAGGGAATAAGGACTGGAAGGTGACGTCATATCACTATTCTGGCCATTTCTTCCCGTTCCAAGTTTCTAGACAATAGGTAGGCTAACCTTCCCCCAGTCCCCTGGGAACTATTAATTAACGTTAGATAGATACCCGAGGGCAGACTTTGTGAAAACTACAGAAGTCCTCCTTTTGTTTACTTTGTTTCTCTGAAATGCAAATTGTGGAGTGCCAAATAATGACAAAACAAAATATTTATCCACTTCAAAATGGCTGCTTGTAAATTTTTCCTGGAGCAGGTTAAATGCTGGCTCTTAGTGAACAATCTGGAATGTGGTAATAACTCGCTTCTTGTTTCAGAGTTACTATTCAGCAGTCGCAGTTTCCACCTGATTTCCGGTTTTCCTAAGTTGGAGGCCTTGAGTAGGTGGTGACAAACCCCTGCCCGTGTCAAATGACCCCGATTTCAAGTATTGACTCCAGGTCTACTATCCCCTATATGCTCGTCTTGTTCTGTGAATCACAGATTAGTTTAAAAGTTTCCCCAAAGTCTGAGAACAATCATATCTACACCGTATCTTTTTTTTTTTTGATCTTTGTACAAAAAGTAGAAGTTATAGCCAGACAGATTAACCTTGTTGTGAACAACTGGTGCATGTGCAACTGTTGAATAGAAATTTTTACCAACGCCACAAACCGGCAAACTCTCCCTGAGGCCTAACCAACTCCGCTTTTGCATGAAGGGCCACCTCCTCCCGGGAACACAAGTGGCAGGACCCTCCTCCTGTTTCTGAATACCAATCCTGGTGGTGTTCGAACGGGTCCCGGGCCCGATAGGACCCAATCAGAGCACACGGACATTGAATCTGTGCCACTCTTTAGACAACTGCTACCACTAGCAAGGAGGCCACGTCACAATAGTCACTTTCCTTTAAGCAAGTCTGTGTCCATCTCGGAGCTGTGAAGCAAACCAGGCATGTCACACAGAATGGGGAGCACACGACTGCATTGCGCCCCTCACTCATTATGCAAGTCATCACCACTCTATAGAAAGTTGGGCTGAAATGTGCAGCGTCTTTCCACACCCCCAATTCATCCCGCATGATCTCCTCCGGCCGGGCAGGGAACCGTCTCGGGTTCCTATGGCACCCCGACTTGGTTCCGCAGAAGCCGCGGCCCGCCCCCCCTCGGCCTTCCCCAGCGATGGCCTCTCAGTACCGGGTCCCTTTCTCGAGCCGCAGCGGCCACCCCGCGCTCCAGGGTGTCCCCGGGAAGGAGCCAGTCGGGGGTTACTGAGGGGGAAAGCAAGGAAAGGCCAGTCCCCATCCTTTCCCTGCGCCGCCGCCGCCGCCGCCCGCCGCCGGGAAGCAGGCCCGGAAGAAGGCAATCAACCAGTCGCTAGTAGGCGAAAGACCGACACCCAGCTTCTGGCAGAAGAAATGAGAGGGGGAAAGTAAAAAATGCGTCGGAGCTTCTGATGGAGAGCCCCCGCTTCTACCGCGCCTCTTCCCGGCAGCCGAAACCGAACCCCAACCCAGCCACCCGCCCGGAGCCGCTTTTCCCTCATCACCCACTCGCCAACCGCCTGCGCCTCCGCGCCGCGGGGCGCAGGAGGCCCGCAACCGCAACCCGCCCGGGCCCGCCCCCGGGCCCGCCCGACCTCTCCCCGGCCCCGGCCCCGGCCGGCCCCGGCCCGGCCCCGGTGCCCCGGCCCCTAGCGCGCCGGACTCTGAGTTCTCCGAGTTGCCTAAGGCTGCGGTGTCCCCTTGTTTTCTTTCTGGTTCATCTTTATCAGGTCTTTTGTCACCCTCAGCGAGAGTACGTGAGAGCAAGTATGTGGGGAGAGACGGGTGGGAATAAAACAAAACACACCACCCCTCAACCCCACCTGCTCTTGCTAGACCCGCCCCCAAAAGGAGCAACGGGCAGCAGGACGGCTCTGCACACCAAGCGTATCTTTTACGTGGGCGGAACCTGTCGCGTTGAGCACCCTCTTTCCTAGGGACACCGTAGGACCGTCTGTCTGTTTCTCTGTGCGCGATTGACGTTTTCTCACGCGGCTAGCGGAAAATGGGGCGGGGGCAAACTTGGTCTGTTCTTTCTCTTTAAGACCCGCTCTGGAGGAAGCGTGGCGCATAGCGTGGCGAACGAACCTTAAATAGGGGGAGGATGCCTGGATCCGGAGAAAGTGAAAACGCCGATTCGTGGTTTTGAATGGTTTTGTTTGTGGCTTGGTAGGCTCCAGTGGGCGCTCAACAAATTTAATTGGTGGCTGAAATTTTGTTTTTACCGTAAGACACTGTAAGTGCATTCAAAAAAAACTCTCCCACGCAAACCTGGTAGGACATCTCCTGCACGCCGGGCGGCACCCGGTCCCTGCAAATCCTCCCCAATTTGCCTTTACATGTTATAGAATTCCTCCTCAAGCATATTTCACCATGGGGGAGGGAAGTCATTTTGCTCGTTGCCAATATGTGGATGGTTGTTTTTTTTAACTTTTTGAAAACGTACAATTCTGTCTGATGTGTAAAAAACACAAAGTTTTTGAAACCTGCGTTCTTTGGTCTGCAGGTGTATATATTCCACTATAAGGATGAGTATCATTTACCACCACTCAGCTGTGTAAAAAAACAAAGGTGTTTTAAACTGTGTGCTTTTGATCTTGCAAGTTGGGATGGCACTTATACAGTGAGAGCATTTCCTCTTTCATTCACTAAACATCACACAGACCTTTTACTATTCACCGCGGCAAGAAAGGGAGGAACAATACACAAAATGGGCTTCACGTCTACCCTTCTGCAGAAACAGGGACCCTTTCTACTTTCTATGCTTTGTGAAGTTTGATCATACAAATGGGTCATTCTTTTATTACCCATACTAAAAAGTGGGGGTAGGGGGTGGTAGCAAGCATTAGGAACAAATGACACTGTCCAACAGTTTGTAATCTCCCAAGTCCGCTTGCGCAACTGCCCGTTGTGTACTGAGATCCAGGTTTATCTACTAGTTGCTAAAATGACCTGTGCCAGCTCTTAATTTATCTACCACCTCACTCACCAGTTTGAAACTCACCAGCTTCCTCATATCCTATAGTGCCAAGAAGTTTCTCAAAAAGGATGCACTATGAATTCTATGTAACATTTCTCTTTTTTAGCCAAACTTACCCTTTCTTGTTTGTGTGGATCACCGATAGACCATCGATTCACATGTATGCCTAATGCAATTCTTATTCACCAAAAAAACACTTAATTTAGAGATTCACTCTGATTTTATTTGATGGACAGCTTATAACCAAGTAGCTAGCATTTACCAAGTTTCCACTGAGTTGTACTTAATTATTACGTGGAATTAAAAAAAACTGAATTTAGAAACAGGAGTAGACCCTTTGTTGTGGGGGTGGTTGGGGGAGGAAGAAAATTGTAGGGTAGGTACAAAGTGCCGTTTACGATCTAATACATCTTAGAGATTAAGTACAAATGATGGACTCGCGTAATAATTGTGGCTTAGTACCATTCTTTACACTGCTAAAATGAAATAACTGAACTTGTGTAATTTATAAAGACAAAGTTAATTCTCACAGTTTGCATGGCTGTACAAGCAGCATTGCTGCAGCTTCTTGGCGGCACAGGGAACTACATCATGATCGGAAGGATAGGGAGGACCCCAGACTCACATGGCAGGAAAGGAACTGGGTGGAAGAGAGAAGAGAGTAAGATGGGAGTGTCTACTCTACGTGTTTAAACAACTAGACTTGCAGAAACTCACTATAGATGTACCAAGCGGGGGGGATGTACGAAAACATTAGAAGCCACCCATAATCCACTCACCTCCTCCCAGGCCAACCTCCAACAGGGGATTCAGTTGCACAGGAAGATTGGGTGGGTGACAGAGATCCACACTCATGTTATCACTCTGGCCCCTCCCAAATCTAGTCCTCTTCTCATATGGCAAACTACTGTGTGCCTACCAAAAACAGTTCCCCAAAAACGTCTTAACTCGATCAGCATTCATTCAAAACGGTCCAAAAGTCCCAAGGTCTCACCTTGATGACGAAGTAGTCTGTCCTTCTACCTATGAACCTTGTTATAAATCAAATAACTAGGGGTAATGTTCAAAGATACTAATGGGGGTATAGGAGTTGGGCCCGATACTGCATCCGAAAGGGAGAAATTGCCCAAAACACTTAAGGCTCTATAGGGCCCCATTGCAAGTCTGAAAGCAGCCGGGCGGCAGTCATTAACTGTAACAGCTCTGAAATAATCTCTTTGGCCTACACACCAGGGAACATGATGCAAATGAGGGGGCTTCCCAAACCTTTGGGCGAACCACCCTGTGGTTCCCGGGTTCATTTCTCCCACAGCTGTTCATGGGCTAGCTTGAGTGCTGCAGCTTTTCCAGCGCTGCAGCGTGTTTGCAAGTCTGTTGGTTGGATCCAACCATTCTGGGGTTGGAGGACTGTGGCTCTCTGTCCCTAGCTCTGCTAGGCATTTCCCATGGGACTCTCTGTGGGGGTGACCCCACATTCTTCTCCTTTGCTTCCCTGTAGATGTTCTCCAGAGGATCCACCTCCAGAACAGGTCTTCTGTCTGACATTTCCAGGCTTCTTTCATACATCCTCTAAAATCTATGCAGAGCTTCTTAAGCCTCCAAACTCTTTCCATGTGCGCCCGCCGGTTAACAGGGCTTAGGAAGCCACCAAGGTAGCTTGCACCCTGTGAAGCAGCAGACCTGAACTTGTATATCTACTGGGTGAACTTGAAAAGTATCTGAGTACCATGCTGGCAAATTCCATGGTGTCTTGACAAGCAACTCTCATTCTGGGCCCTCGAAGACAGATAAGATATTGATCCAAGCGCATAAGGCAGAGTAAAAAGCGACTGCGACAAGTTTCAGAGCGGAGTAACAGTATTAAAAAAGCTTTAGGAACAGGAATGAAAGGAAAAGTACATTTGGAAGAGGCCCCAAGGGCACTTGGAGGTCAAGTGCTCCTGTTTGACCTTGAACATAGGTATCTTTATAGCATGGCTAATTCTGACCATCTGTTCCCCTTTCCATTGGTCCTTCCTCTAAGGGTTGAGCTCTGGCCGCAGCATGGTTGCCCTTGCTTGGCAGGAAGGTTGAGCGGTGCAGTGGTTATGGAGTTGTAAATATGCTACGAGTCTTTCTTCCCTTTTCGGGTGGACTGCCCCCAAAGGTCATACCTCACCATTTGCTCTTAATGTGCATGTTAAGCCAATCTTAACGTACTGCGATCTTATGAAGCGCCGTTACCCAATTCAGGGTGTTTCTATCTTTGAGACGTTGCCTCTCCTGTGCTGCCTGCAACCAATTAACATTTAGAGAGGGCAGTATGATAACTGCCGACCATTCATTGATGGTGCCTGACATCGCTGTGGGTGGTGTTGGACTCTATCGTCCCCCATCCATGCCTGATTAGCTACCTACTGTAAACAGTACCTGCGGCCCCTTTGAGAAGCTGGGATTTCAGGGAGCAGAGTCCAAGGCTGTTAAGCGGGCGCAGGGGCCCTAGGCCTGGCCCAGGACATGATTTCAGTCCTCCAGGCCGGGGCCGGCCTGGATGAGAGGGACCACCATGCAAAGCCTAAATAGCCTATTCGGAACACTTTACACTGTGGTGGGACTGAAACTAGTTCAACCATGGGAAGTCAGTGGGCGCTTCCTCAGGGATCAGAACTAGAATACATTTGACCCAGCCATCCATCACTGGGTATATACCCATAAGGACTATAACTCGCGTATATAAGCCACCTGCACACTATGTTTATTTTGGCACTGTTCATCAATAGCAAAAGACTTGGCACCAATCCCCAACGTCGCCACAGTGATCGATGGATAAGAAAATGTTGGGCACCCATATACATCCCTGGAAATTACTATGCAGCCATAAAAACTATGGAGTTCATGTCCCTTGTATGGGGACATGGATTAGTGAACTTTGGAATCGTCATTCAGTAAACTCTGCGGTTCAAAAAACCAAACACCATTGTTCTCACCAAGGTGGTCATTGAACAAGATGAACCCAATGTCACAGGAAGGGCATCATCTACCTGTGGGAGTTGTGGGGTGGGTGGATGGGGGAGGGATTAGCATTAGGAGATAACCTAATGACTAAATATTGACGACGGTTAACTGGGTGCAGTAACATCCAGCATGGCACAGATTATCCTATGCCAACTAAACCGGCACCGTCTACCCGTACCCTATCCATTAAAACTTAAAGTATCATAATAATCATAAAAAAAATAACATTATAAAGTAAAAGCCTATAGCCTATGAGTCTTTTTCTTCCGCCTATTTTGGTGTTTTTTGTCTCAGCCCTTGGCCTTCCTCTAGTTGCAAATTTTATTGCGCTGCTTACTTTCCTCTCCTGGAGAACGAGCTTTCTTTATACCACCCTGGGGCTCAGGCTGCCCTAAAATTTACCAATTTTATGCTCGCTCCCTTTTAGTTGGTAAGTTCAATTTTCAGGTCATTCTGTGTCCGGCTATGAGCTATAGGCGTTTAGAAGACGCGAGGGTACGTTGCACATCTCGGCTGCTAGATAATGTGGTCTTCTGCAGTAACCCTAACGCAGTCAGGTCTGTATATGCTAAGAGTTACATGAAGCCCTAGAACAGAGGAACAAGTGCAGGCTAAGCTCTGTGCTAACTGCCTATAGCAAACTGACCTGTACGATCCCACTCAATTTGCTCAGTCCATCTGAGACCTCCTCGCCGGGGACGTCACGGCTAAGTCATAGCAGTTTTGGTTACAAACGCACGACAACAAGTTTCCTAGCGAGTTTCCAACTTCCTCATCTTTTGTCTTCTTCTGAGCCTCTTAAAATGTTCAACTTCGCCGTTAGGCAGGGTTCCAAAGCACGCTACTTTTTCAGTGTATCTTTATAGCAAATGCCCTTTCTCGAGTACCAATTGTATTAGTCCATTCTTGCATTGGCTTAATAAAGACATATCTTAGACTGGTTAATTACAAAGAAAAAGGTTTAATTGACTATGGTTCTTGAGGCTGTCTAGAAGCAGGCTAGCATATGCTTCGGGGATGCTCAGAGAACTTACCAATCATGGCGGAAGGCCACCATAATGAGGGGAGCGGCACTTACAGAGCGGGAGCCAGGAGGAACAGAGAGAGGGAAAGGGGGGGGGTGGGAGGGTGTGGAGGGATGTGTACACACTTTTAACCACCAGATCTCGTGAGAACTAGCCACTATACAGTACCAAGAATAGGAGGTGCGGAAACCATGAGAAGCCACCACACCCTGTGAGACAATCACCCCAACCAGGGCCATCTCCATACGTGGGGATGCAACGGAACAGAGATGTGGGATGGGGATATGGGAGCAAACCATATGCCAATTGAGACGGGAAATTGGGTAGAGAATTAGAGGTGAGAGACATCGGTAAGCACATAAACTCAAAGGGTTGTAGTTGAAAGAGGAATTGTAATTGGCGGGACTGGTAGTAGGAAATCATAGCACGGGGGATAGCAAACGCAGTAGTGCCCATTGTCGAAGACATACAATTAAAAAAAAAGAACACTACATCCTCCTTTGGGGGGGGGGGACATCTCTAGGATTTGTAGATATGTCATCCTAGGATGGAAGATATGTTTACAGAGGCGATAACTGGACCTCTGCGAGCTTGCCTTATTAACCCAAGGCTACCATCCTACTTTGATAATAGGCCATAGTTCATGCCATCTAGGGTCACTTGAGTCCAGAGGACAAAACTATTGGGTACCAATGGTGATGATAACATCATCTCAGATTACACTCCTATGGTGGGTAACTAGCATCCCCAGTTTAGGAGAAGACTGGCCCTGGATAAAGTGTGGGAGGGGGACTACAAACCAACAAAGCAGATTTTCCTACTTCACAATTTGGGGTCCTACAGACAACAATTTCCTTTCCGACTTAGGTTTTTCTGTTTTGTGATATCAGACCCGATGATAACAAACTAGTTTCCTAAGAGAGAAATAATCAACACCACAGCCTAAATTCTACATGATCATGAGAAATATAAAATATTCGGGAAAATGGCTCTGTATTGCATGATGCTTCTAGCCAGTGACTCGTGTCTCTGCCTCCCCCACCTTAACCAACAGGTTATTTTAGTCATTGTTGTTATCAGTATAGTAGCGTAGTCTTTATTCGATTGGCGCAAAAAAATCAGGGAGAGAGAAACAATAATTATAACTATCGTACAGTGGTCCAAAACTACTTTATGCCGAACGGTATTAAACGAGGCGGACAAATATTCAGCAATATGCAGTGCTTCTGGGAACCAAGAGAAAAGGCTTATTTACGTATTATAGTTAATGTGTTTCATTATAGCAGCAGCAAGATGGCTGGGAGGCTTAACCCTAGCCTCATTTACTACTGTTAAAATTGTTATTAACCACATCAAGTAAAGTAGCATCTAAATGAAAATAGTTTTTTCTATCAGAAAAGATGTCTTCAAAGTGAGAAGCCTAGTCTCATTTTCACCCAAGAATACAACTCCCTTAACAGGACCTTACCTCCTTTATATAGAAATTTTTGATCACTCTTCAATATACATTATATTTTGCAGTTCATACAAAAAGACTCTAAAACTGTGGACAGCAGAAAAGCTAGGTTAAGAGCGTTGTGGGCTCTGGATCAGAGTGGGTGTGAACGTTATTGTCTGCATTGATTAGTTATGACATTTTTGGGCAGTTAGCTATTTCTGTGTCGCCTCGTTTCTCCCAGCTACACAAATGGCATGCAGACGAATTTCTCCTAGGATCAGGTGAGGGCATGTGTGCCAAGCAAGCTAATGATTTTAAGAACACAGTCACATCGCGAAGTGGTTCTAATCCTCAATTTTGTACAACTAGCCGGAGCCTAATAAAAGCAATAACCAATGACTTATAGCCTTTTTTTAAACTATGTACTATGTATGCTTCCATAACTTTCATCCGAACTCCAGATTTATACCTTTCTTGCTGTGTCTAAACATGCCGTGTGTCAGGAAGGCCTTCTCGTCTTAGTTGTACTTTTCAAAAATGCAATCTTCGCTGCTTGAATTTCCGCGTCTCGCGCGGATGATGCTTGTGATTCGCTCCATTTATCAGCTTACAGTCAAAAATTATCGTCGATTTACTGTGACTGGCATGTGATGGAAGCTCCTTAGGATAGGACATGGAGGAGGACTTACCGCGCCGCTATCAACCCTAGTGCATTTAATTTCTGTTTCTTTTTTGTTTGATGACAGCGTTTCACTCTTGTCTTGTCCAGCGCTGGAGTTGCAATGGCACCGATCTCAACCTCTCATCCTCCCTGGGTCTAGTGATCTCCTGCCTCGCCTGATAGGGGGATTTATAGGCATGCACTCACCCATGCCCCGTTTAATTTGTATTTTTAGTTAGAGATGGATTCTCCATGTTTGAGCCGGTGGGTCTCAAAACTCCAACCTCAGGTGATCTGCCCCCTTGGCCTCCCGAAGTGATGAGATTACAGGGTGGACCCCCGTGCCTGGACTTTAAGTTCTTTTTAAATAATGAGATTTTATGTTGGCTGCCTCAATCTCTTTACAGACGGTCGGTATGCACTTCTGGTACTATCTTTGGTGGAAATGGAAGACCAACACCTTACGTAGCCTTCTAGGTGACATCTTGATTTCTGCGTATATTAAAGTACTTTCTGGTTGGCTCCGGTGATATAAATGGGTTTACAAACCTTGGGGTTCTGTGCCTGGGATCCACCACTCTACATGTTCTGGATGATAGCCAATGTAAACAATGCCCTTGCAAGAGAAATCTAGGCGGGTTCGTTTGGGGCTCACGCCTGCAATCCCTCAGCACACTGGGTAGTGTAGGCCGCTAGGTGGTGGACCATTTGAGCAGGGTCAACACCAGCCGGACGAAACAGGGTTGAGATGAAAACCCCATCTTACTACAACTACAAAAAAAAATTAGCCAGATGTTGGGACTATTGCTGTAATCCCATCTACCTTGGAAGGCTGAGGCAGGAGAGTCTCCTTGAACCCGGTAGGGTGGCTGGTTTGCAGTGAGTGAGATAGCCACGCACTCCAGCTGGGCAATGGAGTGGGGGAGGAAAAGGAAAAGAAAAAAGAAAAAAAGAGAAATATCATCGGCTCCACTATATATTTCCTTGGTTTTTCCTGGGGACTTAGATTCTATAAAATAATACAGCAATGAAACTTTTCAACAGAGAATCACATCTTTGACCGACATATTAACCTTCTTTCCTTCTCTGTTGTATTCTTGTCCACTTTATAAAGCAACAGTTTTGCGGTTGATCTAAGGCCCCTGGACTACCGCGTTCTGGGAGAGTGAAGTTTCCTTAGTTCATGCCGCCTCAGAAAATCACCAGAAAGAAAAGAAAGAGAACAGAAGTAGGAAATGAGCACGCTTCTTCTTAAATTTAGCAGTCAAGACCACTAGTTATAGAGGAAGAGACGAATTTGGAACTAGCTACTTGAGAGGTTGGTCAAAATTGATGGAATTTCGATGGAAAGAACAGGCACAATGTGTGGTTTTGTTTCACTGGACCTACCAATATGCCGAGTAGTTGGGGGACTATAGGCAGATGCGTGCCTCATGCACTTCACGTATAGCAATCTAATGGCCCCCTCTAGACCTACTTCCCAAATCACTTCCGAATGTGTTTCCCTGAGCAAACCGGCCTTAGCTGTACCCCTTCTATTGCAGCTTTCCCTATTTTGCTATGATAGTTGTATTTCTGAGTACTGGATAGTGCTTTTGTAAGTAGTTCCTTTAGTTTATTTCCATGATATTGGAACTACTGCTTCCAACATGTCACATGCTATTTGCTGACCTGGCCTTAGGGACAGTATTTGTTTAACTCTTTATGTTCGTTCATACAGATGATTAGCACATTGCCCGTGCGTAAGTGAAATTCACCCAATACCAGACTTATTCTCTCTTGAGTCCGGCTATGTTTTAATATCATGATGTATTTGGAACCTTACTTAAATATCTTATTGGTTGTGTTGTAAATTTAAAAAACCCCAACAAAAAACAAAACAGAAAGCAACAAAGAAAACAAAAAGAAACAAGTTCCCTAAACCAACAAAGTTACTAATCATTTCACTCTATGTTTCACCTTTCAGTGGGCTTCTCCATTTTCTATTATGTCCAGGTCCTTGGCCATGACTCTCTCAGTTTTCCAAATCGGATCCACTTGACTTCAAGCTTCTCTCTTACCGGCCCTTCCTGGGATTGCAGACGAATGTACTGTTACTGCTCTGTCACCTCTCATTAATTTCTCTGCCTGAAATTTCTCCTCTCCATTTTGGTGAGCATAACCTTATTAACGCAGCCCTATGCGCCTCCTCCATTGAAGTCTCCTGGATCTTCCTCCGTTTGAATAATTCCATTTGTGCCCTTAACCTCCACTAGTTAATTACAAAAACCAAACTCTTGTATTTACATTTGCCTATTTGGGGGCAAATAATGCTAATAATATTAATAGATACTAATAATTTGGAAACATTTCGAGTGTGCAGATTGACCAACACTCGGCGTTAAGGCATGAGTTTGGAGTCAAAACCTAAACCTAGTCTTGCATTCTGGCTCAATCACTTGCATTCCTTGGTGGGAGTTACTTAACCTTTGAGCCTCAAATCTCCTTTGAATTAATTGGATTTGCTTTGAGGATTAAATGCGGTTTACCCCATAAAAGGGGTTATTTGATACCTGCCCCCATGGTAATTGCTCAGAAGTTTAGTCTACTTATTTATTAACAGGTAAGACGTCAATTTGGTTGTTAAAGAAAGATTCTCCAACCTCCTAGTTTATAGATGAAGAGATTGATAAGGGACAAGGGGTAAAGGAGATTTACTCGAGGTACACCTTTAGTTTGGTGCAGATCTGAAACAACACCTTTCATATGCCAGCAACCAGGAAGAGCTTTTTTTTTTTTTAAATCAACATAAGGTGTGTATTATGCTTTCCATCTATCTAGATGAAGCTTCCGGAGGGCAGGGAAACTTGTCTGATCAATTTGTCTGCGCTTTCCTCTAAGCACAGTTTACTTGCCTATGTTTAGTCCTTTTATAAATGTTGGATGTAATTTAATTAAGAATTCCCCTAGAAACACAAACGAAATTTTCTCCCTTAGCAACAACACTGATAACTAAACTGTATTTAACTCCCATGTGTAGATAATTAAAATTTTCACTAAGCTCAAATAGGCACACAATCAATTGATGTTTTAATAAAGAAAAAGTAATTTTGAAACATATTGAATTTTTAGGGACAAAAACACCAGGCCCACTTATTCATCAACTTTCTAGAAGAGCAAGTTATTATACATTGCCAAGCATAAACGACTAAGATAGATTTCAAAACACATATTTTTAACTGCCGTTGCACCCACGGCATTTAACATCAGTTTTTCAAATTTGAATACAAACAAATATTAAAAGTATTGCCTTACAGAAGTAATGGACTTTGACAAGTTTTATCGGAAGGAATTAGCCCTGTGAGAACAAAAACTGGAGCAAGGTATAAGTGCTACACACAACCTAAGAAAGGGATGGGATTGTATGTTATTGTTTTTCATCTCAACTCTGTAGCGAGCTAGCACCTTAAAGCTTGCTGGGAAATTTACAGGACTGTCTATTTTGTCCTATTTAAGATTGTGAAAGTTTCAATAAATTATGACACAGAGCATGCACAATACTTAGACGAACAACAAACAGCTGGTAAAAGGCGTGTTCTTACTTAAATTATAGTATTAAACTCCCTCAAAATCTTTCGTCCTTGGAACTGATTTTGTTGCCTGCCATCTCCCTGGCCGGATTATAATAGGAATAGTGCTCACACATGTGATACCCTCTGACGATTATCAGGTTTTTACCAACTCGTGCTTTAGACCAGATGCAGATGCTGATGGGGGAAATGGACATTCGGGCTCTATTCAAGCACTCTCCATTATGGAAACTCTACATATAAATCAGGGATGACATGGAACATTTTGTTTTCTGAACACAGGGTCAACAAAAAAACGCAAAGTACTAGTTAAGTGGTCATTGATATAAGATGCCGATACCCAACACCTTCTAGTTTTGTACACAAAGACCAATAATCTTCACCAGTTCTCGAGTTTTTACAAACTGCCTCTTATTCAGGAATCATTTTTTTTTAACCAGTCCAACAAATTCACCAAACAAAGTAATCAACAACATTGCAGTCAGTACCTTTAAAAATGCTAAATACTGTCATTGTGTGAAAAAAAAAAATCCACCATAAAAAGTGCGTTCAAATTCGAGTGCTTGTCTAGGGTAGCTTATTACATATCAGCTCAGGGTGGCCAGTGTTGTTTTAAATAATTATCACACAACAGCAGCTTTCACTTCTAAGTAGAACTCTCAGAATTAAATGCTGGAGTGGAGATAATGCGAGATTCTGGTTCCGGGTATGAAGTTAAATTTTGTATTACGGACAGGATTTTTTTTAATTCACATTATTCATGTGGAGGAAGATTATATATTTCTAACTGGCAGAACAAGCCCCAACGCCAACCTTTCCAGTCTCTCACTGCATCTTTCGCCATACCCCCAAACACCACTAAATTTACAAATTGAGAGTAGGCTAATTCCTTTCTACAGAGCCAACACACCTGCTTGGAGTTTGTAACTAGGGACAGCACTTTGTTCAAAATTATTTTTTTTTTTTTTTCCGTTCGAAAGAGGTTCTCACTCTCACTTGTGCCCAGGCTAGAGTTGGAGTGGTTGTGATCTCGAATTGCAGCCCTGGCCCCCGGGTTCAAGTGATTCTCCCACCTCAGTCTCCCCGAGTAGCTGGGACATAGGCGTTGTACCACCCCACAACGGGCTAATTTTCTGTCTTTTAGTAGTAGAGGGGTTTCACCACGTTGGCCACGCCCTGGTCTGGAACTCCTGGGCTAATGTGATGCCTACCCCTGGGCATCCACAATTGCTGAGATACAGGCATGCCAACTTGTGGGCCAAAATATAATTCTTCTTAAGATTCAAACACCTATTCAATGTGGTCCAGAAACTCTGGGGGAAAAGAAGTTCGGTTGTCTCTTGTTTAAGCATTACTAAATGTGTTTCTTTGGTGCAACCCCTTGGTCCTTTAATAAGCGGCCACTGAAATAATTTTATAGTGTCAGGCTTAGAAGACAATTATTCGCTATTTAACCATTTACAGTTTACGTTGCATTTGGTCAACCAAGACCAGATGATTAAATGTTCTTATATTATAATATAACGAGGATATAATATCTATTTGAATTAATCCAAACCTTTGTAAAAAACATACTTATTTAAATGCAAAATGTGATAGAAGTTTCTTTAGAATCAAAAAACAATTTTAAAAAATCATCATCTCACTTCCCCTCCTCTTTATCCCACCACCCCACATCCTTATTCCAAACTAGTAGGTAAGGCAGGTGATGATGCTTTACCAATTGAGATGCATTGAATCTTGTTTACAGGTGGTTTGAGACTTCGGTAGAGTCAGTGTTGTTGAGAGATGAGATTTCCATCACCAGGAATAGAACTGCACATGAGTTGCATCGAGTTGTGTAACAGGGTAGGAGTTCCTAGGCTCTGGAACCACCTTTCAAGGTGGCCTTATTCGGTTGCCCACAGAGGCCATTAAGGTATCCCCTGTTCATCCTGAGGGTCATGATGGGAATGATACTTTTAAAGGTGTGTTTTAATACTCCTTTCTCATGGTGCTTGGTTACTGCCATCCAAAGAGCATTTTACAAGTGAAACCGCTTGTACTGTAGGACAAGGGAACAGAAAGTTACCAATTACTCTTCTGATTTGGCCAATCCCATGTCAGGTAAAAAAGTTTTAAGCGGTTCAACGGGAAAAACCTTATCAGATGATGTTTGTAACATTTTAAATTGGGAAAGGAATATAAAATAAAGAATGAGGACCTTTTTTGTTTTATCGTGAGTACTTACTTTTCCCTTTTTAAGGAATCCCAAGATGACCAGTGAAAAATCCACTTTTGAAAGCTATTCATGTTTGATAATGGGTGTGTTAAATTAGTTAACTTAAAGCTTCCTCTTTTTTTTGCCATTATTTTACGTTCTTGCTAAAGGTTCTTCTGCGGTATAGTGAACTGTAAAACTAAACTGGATGGGTAATAGACTGTCAATCTGCTCTGTTACCAAATACCTACTTTTGGTTCAATCAAAGACAGCCAAACTGTTAGATTATGTTCAAATAACGAAAATGGAAAGCGGTAACAAATCTCAGCGTTTGGTGCATCTCTCACGTTCCATTTGTAGTCTTCTCTTTCCCTGTTTCTGCAAATAAATCCTATTCAAACAATGGACAGCACTTGAGTTGCTTGACCCTCTTGTTTAGGGGGGTTTGCCTGATCCTCAAATTCTATTTCTTTGCCCAATTAAACTCTGTTAAATTTTGATTGTTTAAAGTTCTTAAAAAGTTTAATGGGTTATCATAATAGGTGGTTTACTATACGTATAAGATTACATGAGCTATTTGGATGCAGCATAAAATATGTAAGTAATCACATTAAACGGTTAAATAGATATCAATGTCACACCTCACATCACGCAGTCATTTATTGCTTCTTTGTGTCAAAGGACATTCCAATTTATGTACCTCTTAGTTATTTTTGTGAAAGCCACAAATAAATTTTTGTGCTTGGTTAAGTAATACCCTGTTGTGCTTATCAGAATAGATCTTATTCCTCCTTATCAATTTGGTACTCGATAACCATTTTCATTCTCCCCCACCACCATTACCCCTCTGGGCTCTGTGTAATCTCAGAATGGGAGAGCAAAGAAAATTTGAAATTCTTCATCTGACAAGGGCTCATAACCGCATATATAAAGGAACTCAAAACCAAACTCTAAATTAGGAAAAAAATGGAGACCGCCCTTTAAACAAATGGGCACAGATGAATTAGAATTTTCAAGAGAAAGATCATACCCAAATGGGCAACTACAAGGATTTAAAAAAGTTGCAACATCACGTATCATGAGAGGAATAGCAAATCAAAACTACAATGGAAATATCATTTCACTCTCGTGCTAATTGGCAGTTGTGCAAAAGGACAGGCCAATAAGGAATATTGGCAAGGAATGTCTCAGTTTTTCTAACAGGTGGAAGTGAGTGAGGCGACTCCTTCTACCTCTTCTGAATTTTTCCTCAGTTATTATTAGCATATTATGTCTCTGCTAGATCTTCCTTTCTTCATATGGGTCTGCCTCCCCTAAGGTCTAATTATGCTAGGTCTGACGGAGAGAGAGGTGCAAAGTGAATCGACAAGACTCCAACTCCTATGTAAGCCAATGGTTTGTTTCCTCCTTACCCCCACCCACCATAAGCAGAACACAAGCTGGCAATAGAGCTCATAAAATACTAATTGTCGCTGGTTTGAAATTTTATGTGGTGGATGAACATTAAGCTTTTTACATATCTAGTAATCAAAAATATGTATTCAGTTTAATTTATTGAAGGAAACATTTTTTTTAAATATACTGTTTAAGAAAATCAAAATATACAAATACATCATTAAACATTGTACCAATATAACATAAACATATATTACCTGAACATGAGAAAATCCCTAACCCTTATGTCTTCTCCACTCACTTACTTTTTTACCCTTTATAGGAAACCCACTTATTCAGCAATTTGGGTTGTACTTATTCTTTCAAATAATTAGGCAGAATAATAAAATATGCAATTATATTACCCTCTTTGAGACCAAAAGGTCGTCCACTTTATGGCTACTTATGTACGTTTTTCATTTCAACATCTGCTTCCTTCCATGTCAGAGCAATAGAGAATCTCTTTTTTTTTTTTTGACGGAAGTCTCGCTCTGCGCCAATGGAGGATGGAGTGCAGTGGCGTAACCTCGGTCACTTCAAGTCTTAGCTCCGGTGCATGCCACTCTCGGCTCAGCCCTCCCGATCGCTTGGGACTATAGGCGCCGCTGGCTAATTTTTTTGTATTGTGTGTAGTAGAGACGGGGCTTCACCGTGTTAGCCGGATGGTCGCTCGAACTCGTGATCCTACCCCCGCCTCGGCCTCCCAAAGTGCTTGGTACAGGATGTAGCCAACCGTGCCCGGCCACTTCCTCCTTCTTCGAAGGGCGCGAGCTTCATGAACAGAGTCATGAAATGAAACCAGTCCCTGTGTGAGGTATGGTATATGAGTTGCTTTCAGGTTGTTGCTATTAACCAAGTCTGTTAATGAATGCCTGCCTATTTACATTTCTTTCTACGGTGAGGGTGTACCCTACGATAAATCCCCATCAGAATGGCGATTAAATGCATTTATAGTTTTATATATATTGTTCCCCTCCCCCCAAAAGATCCCCAAATTTTCTCCATAGGGTGCATCATTTTCATTTTCTCCATTAAACTCACAAGTAATTCATGTGTTCCCACAGATTACCAACAACGTTTAGTAATCATTTTGGATGTTTTTGCATTATTCTATAGGTGAGAAGTTGTTAACTTAGAGTGATATTGTTTTGTATTCTCTTAACTGTGATTGCAGTTGAACTTTTCAGCTGGGAGAATCAAGCTGCAGCGAGTAGCAGCTTCGTGTGTGCTCCTCTCACAGAGAGGAAAACAAAGTGGGAGTAAACCACTGCCTCTGCAAGTAGATCACCGTAAGGAACCTACCGGACAATTAAGGTGAGTGAGGGGGGATCCACAGAGAACAGGGAAAGAGTGAAGCTGGGGCTGGCCCCCTCAATGCGGGATGGAGCCAGGGGAGGAGCCCCCAACATAGTAGAAAGGTGATGTGAGTGAGAGCCCCTGGGGGATCCGTCACTTCACATGGACCTGTGAATCTGGGAACTGGAGGAGCACCCTCATCCCAATACCGATCCTGTGCACTGGGCCTCTAGACTGCATAGAGAGTTGCTGGGTGTTTGCAGATGGCATAAACTCTAAGTTCCACAATGAACGTCCCAATGCCTTGGACTCGGAGCATTTAGCCAGTCGCCATAAGCCCGAGAGAAGCTCACAGGTAGGGAGCGTTACATGCTCACTCCCCTTTCATCAGACAACGGTTAGCATGGCTTCCCGCACAATGGCCTGGCCTCTGCCTGAACTCGTGGGCAGTGCACAGCTCGTTCTGTTACACCACTGGAAAATCAGTTTGGACGGGTTTGAGAGACTATCACCTACCCCCACTGCGTAGACCATGAGGGGGGCTCATGGTGCTCAAGCGGGTGGCTCTCCCTACTTCAGAACACGAGATGGGTGAGACAGCGGGTTCAGGACGTCAGGAACAGGCGTGTGCCTCCTGGGCAGGCCAGCCCAGGGAAGGTATGGTCTATTTTCCAGAGCCGTCAGCTCTGCCGAGGGCACCCTGAAGCCCAGGAACCTAATAACTGAAATGAGGAGGGCAGTGCCAGTTAATTAAGAGGGTCCACCAAGGTCCCAGGAATGTGGTGGGGGGTTCATCTCTCCTATCCACACAGCGCACTACGAGGACGCAACCAATAATTACAAAAGAGCTGTTGGACGAGTAAGAGCCTATCTTGCCAGCTACCACTCTTAAGCATCACTCCGGATTGCACCCCTAAATGGACAACACAATAAACATTTCCAATATACAGCACTGTTGAAACCTAAAGCAACACGTAGCCACAACTCTTAACTGATGTGACAGAGCTTGGCCCTTTGAAGCACTCAGAATGGAAGCAACTGACACTAACTTCAATGTACCATATTGTAATGGAACACCAGCCCCAATGTTACAGTCCTCTCAAGAAACTCTCGCAATCAAAATAGCTGGTGTCTCCCTACCTCCAGACAAACACACAAGTCCTTCCAGCACCCGGATAACCAGATAAAAGGTAATCAAATGACACCATAGGAATTCAGAATCTGGATGGCAAGGACCTGTACACACGAGCCAGGAGATCATGAAACCCAAACAATGGAACTCAATGGAATCCAGAAAAATGATTTCAAATGAGCTTAAGAGATAAAGCTGATTTTATAGCACAGAACAAAACTGAACTTCAGAAGGAAGAATTGAGGGATCAAACTTCAAAATTACTAATATAGTCAGAGGCATTTACACAGCAGGCAGAGAACAGAACAAGCTGAGGAAAGATATATCTCAGAGTTTGACGTACCAGTTATTGAATCAAATGGTGGACAAAAATAAGAAAAAAAGAAGTTAAAACAATGAACAAAACCCTTCAAGATTTATGAGCGTATGTTAAAGAGACCACAGTCTCTGACACAGTCCTTCCTGAGGAGAAAGCAGAGAGTGTGAGTAAGAACTTAGTAACATTACAGCTGAGGATGCAGTCCATCTGAAATCTATTTCCCCCTATCTCATCCGAGTAGGTGTGGGCATCAACACAATTTCATCGAATACAGAAGAAAGAGGACATCCTGGCGACCAATTTCCAATACAACCATCCAAGTCCAAAAGTCACAGACTCACCAAGGTCACTGCAAAGAAAAAAATGGTTATAGCTCAGCTCGTGAGTAAATTCAGGCATCACAAAAAGCACCTCCTCAGGCCAGCCAACTAGTTTGTCAGCAGAAAGCACACCCTTACAATCCAGAAGGCTATGGGGGGGGGGTGGGCCTCCTATATTTTCAGCCTATTAAAGAAAATAAATTCAACCAAGAACACATATACACCAACTGAGTTCACTAAGTGAGGAGAATACAATCCTTCTCCGACCAAGCCCAAGCCCAAGGAGATCATACCACCCGATGCTTCTACAGGAACCGTAAGTGCTGGCTAAACATGGCAAATGAAAGACGGTGGGATGGTCAGAAAGTGGTGGGATGGTTTGGGGGGTGGGGGAACGGGATGGGGGTTGGTGGGAGAATGGCGGTGGTGGGGTGAGAGGCTGATGGATGGTCTTGGAAACCGGTACCATATACAAATACACTATGACACAACAGGCTACCCAAAAACAACCAGCTAATAACCAAAACACAATGACAGGAGCAAAACTCTCACCTGTCTATGCACCCCTAACTGTAAACCGTTTCCCACTTCCTTTAAAAGCATATAAGTTGTTTAAGCGGATACATATAGACAAGACACAAATGTCTTGTCGGTTTTCAGAAGATGCATCCACATGAATGACACTCCACCGGTGCTCAAGTGAAAGAATATGAAGAAGATCTTCCCCGCAAACGACAATACACAAAAGCTTGCAGTAGTGGCGATCCCTTAAACAGATTAAAAACATTACTTTTAACCCAACATACAATCAGGAAGGACCAAATGTAAGGGGCATTATAATAATGATAACAGGTTCAATTGAAACCCAAATAACGGATTAATATTATAAACTATATGCACCCAAACACGGAGCACTCCGTATTATAAAAAATTATTGGCCCTAAAATAAAACGTAGATGGCAAACCACCCACTAGTGGGGGCTTATCACTCCACTGATGCAATCTAGACAGATCACTGAGGCAGAAAGAAAACTGACTGAAAGGGCATTCTGGAATTAAACTTGACACTTGACCAGTTGGCCCTAATAGACTCTCTCTCGGCTTACTCAAGCCAACAATGACAAGAACACCAATCTTCTCATATGACATTGGAAAGATTTACAGCTTGCCCACCCTGCTAGTTCTAAAGCAATCTCTATAAATTATAAAAAAACTAAAAATATACAAAGGCCCACTCTCCAAATCATAGGAAAACTAACATAGACTATCAATTCCAAGAACGATCTCGCAAAAACTACCAACAGATGGAATTAGCCAATGCTTTCTCGAAAATACGTTGGTGTGAAAGTGAACAATTGAAATTAGGCAGAAATTCAAAAAATTCTATGTGAAATTTAATGAAATAGCGATCACACCTTTCCAAATTCTTTTCGGTACTGTATCCAAAAGCAAGTGTTTAAGATGGAAAGTTATAGTGCTAAAATGCTTCATCAAGAGGTTAGAATCGCTCTCTAACAAACACTAACATAAACACTGATGTGTAATAGAAAAAAAGAGCACCAATTCAATCTCACAGCTAGCAGAGGAAACAGACCTAACTAAGACTTCAGAAATGGACTAAAGTAAAATTGAGATGCATAAAAGTCAAAACTGTTAGTGATGAAACAACTAGTGGTCTTCATAAGATAAACAGACTAATTAGCCTGCTAGCTAGAAAACGCAGGAACAACAGACGAAGTTCCAAAAACGCACAAGAATATGACAAAAATGACATACGACCAATCCCACAGAAAGCCCAAAGATTTCAGAGACCGTTAGAAACCCACCTCGCTGCACACAAAGTAGGACACTCTAGAGTGAATATGGCTAAATTTCCTGGAAAAACATTACAACCTCCCAAGATTGGAACCCGGACGGAAAGTTGAATAATACTGTGAGACCGACACTATACCAAGTTCCCCAAAAATTGAACAGTAATACAACAACCTATCTCACCAAATTTAGCCCTGGACCACGGGAGTCACCAACTGAATTTACCCATAGCATACACAGAAAAGTCGTATGTAATCTACGAAACTTATGCCCATAATATTAAGGAGAGGGGCTCCTCCCTAACGCTCATTCCTACAATGCCAGCATCATCCTGATAACAAAATCTGCCAGAGTACACCAGTAATGAACAAAGCAAACTTCAGGATCCATATCTTCTGATGCACAGTAAACACGATAAACATCCTCAACAAAAATATGCAAAACTAAATCAAGATAAGGCACAATCAAAAAAGTTTAATTCAACAACAATTCCCAGTAGGCTTTATTCCTGGGATGCAAGGCTGGTTCAAACATAACCAAATCAATATAAGGTGATTCTACCACATAAACAGAATTGAAAACAACAAAAAACCATATGCTCATTCTCTATAGTTTCAGAAAAAAACTCAATAAAATCAATATCCATCATGTATTTAAAAACTCCTCGCCCGGCTCGGCATAAGGAATATTACCTTCGCATACAGATTTCCCCTTAGACACACACAGCCAAACATCAGACTAAAAACAGAAAATGTCTGGAAGATTTCTCTTGAGTACTGGCAACAACGACAAGGATGCCCACTCTCACCACTCCTTTCAGCATTGCCCGGGAAAGTCCTAGCTCAGAGCACACTAGGCTAAGAGAAAAGAAAATATAATGGATCCCTAAAAAGAAGAAGAAGTCAAACTGCTCTCTTGCTATTATGATATGATTTCATACATAGAAAACACTAACGACTTCGTCCATAAGTCTCCTCGAACGGCTAAAATACTCCGGAAAAGTGTATAAGTATACAAATCAATGTACAAAAATCAGAGTAGCAATTATCCACCAATAAATTAACATTCTAGGTTAGGGCCAAATAAATAAAATACAATGCCATTTCTAATAGCACAAAATAGGCATACATCTAACCATGGAAGTGAATCTGTTCTACGGTTGAACTACAAAACACTGCTTGAAAGAAATCAATGCCGAAAAATATAAAATCTACTCCATGCCATGGAGAATAATTCAACTCATTAAATGCCACACTGTCAAAGCCAAATTACCATTCCACTGCTATCCTAGCAAAATATCAAGGTCATTTTTTCACAGAATCAGAAAACTATTCTTAAAAATTCATTATGGAACCAAAACGGAGCCCTGAATAGCCCATAGCAATCCTAAGCAAAAGAACCGAAGCCAGGAGACCACATACCCCAATTTTTCAAACTATCTAAAATGCTTACAGTAACCAAGCTCTCAGGGTTGGTACCAAAACAGCACAGAGGAACATAGAAAACAGCAAGAAAACCCAGAAATGGGGCCCCATACACTCTACGACGATTCTGATATTCAACAAACACTTGCCGAAATTAAGCAATAAGGACAGGATTCTTCTTCAAAAATGGTGCAAGATAATGGCTTAGCCCTATGCCGCAGATGAAATTGACTCCGCTTCTCACCAATACATACAAATACCTACACGAGCGATTAAAGATTTAAATGTAACACTTAATAACTTATAAAATCCTATGAAGAAAATCCTTAAGAGAAAAATCCTCTCCACGTTGGCCTTTGGCAAAAACAAATTTGATGGTCTTAAGTACAGAGAATAATGCCAACAAAACTAAAAATCGGCAAGTGGGACTAATTAAAGAGTTTCTGTACAGCAAAAGAAATTACAACAGCTTACACAGTGCACAGAATGGGAGAAAATATTTGCAAACTATGCATCTGAGCTAAAGATTAATTTCCAGGAATCTACAAAGAAACTAATCAGCAAGCAAAAAAACCACACTATTTAATTGTTGAAAAATGGGTAAGTGTAAAGGACGACTGAAAGACGGTCGATCTAAAGAAGACATACAAACTGCCACTAAACATATGAAAACCAAATGCTAACATTCACTAATCATTAGAGAAATGGAAATTCCAAAATACCCACAGTTGAGATACCATTTCATCAGGTCAGCAAAAGGCTATAAAAAGACTAAAACATCAACAGTATGCTGGTGCAAGACTTGGGGAGAAAAGAGAACGGTATACAAGCTAGTGGAATGGATGGGAAAGGGAAATAGTTCAGCTACTGTGGGAAAGCAGTTTGGTAGTATTGTTCCGAGAAACTTGAAAACCAAAAAACATTATTCAACCCCAGCAAGTCTTACTGGGTAATCCAAATAAAAATTTTTGGTACCAAAAAGCTCCATTTACTTGTATGTTCTTGCAGCCATATTCAACCAGACTCGAATCTCAAACCTAGGGGTGCCCATCAACAGTGGGATTGTATTTAAAAGAAACGTGGACATATACACCAGAAATACTACAATGTCCATCAAAATACTGAAATCATGTTCTTTGTAGGCCAATTATCCTAAAATAATCTATTCACACGATGAACCAAAAAATCCGGAACAACTGCATATTTCAATTAACGGGGAGTAAATACGAAACCATTGACATTTAAAAGTTTAGTAGCACGATAAACCCTCAAGGAGACTCGATGGGAGAGGGAGGAAGCACGGGCTAAAAAAATTCACCTATACGTACTATGTTTACCCCTGGTGCTGGGCCATCATTTCAGCATATGCAAATACCCTATGTAACAACCTCCGCACATTACCCTTGAATCCAAAACAAAAAGAAAACAACAAAAATGCCCTCTTCTACTATACTGTTAAAATCTATGATGCGAAAGCAATTTTACAAACAACCTACCCATATTCTACACATTCTACAGTCATAGTGTTATATATATTAAATAGTCATATTAACCTTCGTATGTTATCATTCTGTCCTTGCTTCATCCCTATTGAATGTCGTATTGACGGCATTTATTTTTTGTCTGGTAGTTCTCAATGTATTCCTCAGTGCCCATCTGCGGATGTGTGGGAACAATCAGAGTAATCAAGGACTAACCCCGCATTTCACTCGCCTGAAAACGGAGGTGAAATCTCAGCGTCATAGGCGACAGGAGGAGTGAATGAGAGAAAGAGGGAGGAGTGAAGAGAGAAAGAGGGCGGAGGAAGACGAGGAAATGGTAGAGAGGGAAGTAGGAGCGAGAGGTATAGAGATGAGGCAGAGGGGAGAAGAGCGCAGCCCTTTGGGGCCCAGACCTGGGAGCTCCCGAAGCTATGGGCGTGATTGCCCTTTGGAGGCCCTGAGGGTTCCTGGAGTCTCCAAGTCAGGCACCAATGCTGTTCCGTTGCTGCGCTGCTGTGCAGCCCCGCTTGCAGTGAGGCCAGCACCTCGCCAGTCACTGAAGTGCCCATCCCGCTGAAGCCAGCCCCTTGAGTCCTGGACGTGTGCCCAGACCCCACTCCCCTTCCTACACATCCCTCACCGCTTCGCTCCAGTCTCTTTTGGAGGCACTGGGATCCACGCTCAGTCGCTGTCAGTATGAGCGCAGCCTTGCCATGGATGAGGGGCCCCGTGGACCCGAGCAAAATCACGCAATCGTTTGCCCCGCCACAGGGATTCTGGACAGAAAAGCAAAACCCCCAAGGTCACATAACCGTATCAGTTATTTTCAGTCAGGAGGGAAAATTGCTCTATTCTGTTGAGCAGTGTTGCTAAGGGTTTTGTCATTTAGGTAGGTTGGATGTATCAGTTTCTAGGGTTTCGGCTGTAATAAAATTTTACTACGAAACTGCGGGCTCAAAAAAAGCCATGTATCCTTTCACAGTTTTGCGGGCAGAGCCAAAAACAAGGTGGGGTAGGGCTGTGCTCTTAAAAAGGCCTTTCGGGAAGATTCTTCTTGCCTCTTCCATGCTATGGGTGGTTGCTGATAAGTCCCTTGGTTCCTTGATTTGGTGGCCACCCACTCCAAATTCTCTGCGCTGCTCCAACATCTTCAAATTCACCTTCTCCTCTTCGTTATGAGGATCTTGTCATAAATTGTAGCGGGCCCACCATATGTAACCAGGATGATCTAATTTCCCACTATCTTTGCTTATATTACATTGTGCAAAAAAACACCTCTTTTTCACAGATTCCAGGGATGGACATGAACATTTTTCAAGTGGGGGGGGCCACATTTATTTGAACCCTACTACAAGAGTTTCAAAAGCTCCTCTCGGGGAACAATAACCATCCCTTTACCTCACTGAATGAGTCTGTTCATGTGGGAATGGTTAGAAGATCCCAGGACCTGAAGCCTTTTTCCCATCTTTAAACAGACACCACCCACATTCATGTAATGGAGCAAGTGAATCTTGAGCACGAAGTAATTTAATCTTCATCATCTCTGTTGCTGGGTGCTAGAATTAAACATGTTTTCGCCCCCCATTCGACGGTTCCAGGGAGTTGTAAGTTCCTCAACACCCTGGCAAAGCCTTTACCTCTTCTTTGAACTCCCGCTATGTGTTGTGCTATGGGTGGGAGGGGGCATTACTTATGACCTCCCTGTCTCTCTAATTAACCTGGGGTAAACGGAGACGCGGACCGCGGCTCCACCTCTATCCCCTAAACCATTCACCCCCCTTGGTTGGGACATTCCTTCCTGACGCAAGTCATTCTACAGTTGTGTCTTCCCAACAGTGGCAATTTCATTTGTTTGTTAGGACAGCAACCTACTAGGCAACTACTTCACATCCCAATAGATAACCCATTACAAAACACGATCAATATTTCTGTTTTTCAGCTTCGTGTTATTTTTGAGAAGCCAGAAATTTTCATTTATATCGAGATATACTCTATTCTGATAATCCAGAAAGAACCACCGATAATGACTGGAGAACACTCAAGATTAAAGTGTCCTAAGGTAAAACCATTACTTCCCCTGGTCAATTAATAATGATTATAAATCCACTAGTACTTTTACCGAAAGAAAAATGAATCCATTTTTTTTGTCAAGAGCCCATAGGAAGTTCCGATTTATCGTTGCATTTATTGTTTTAAACTTCTTTTTAAGTATTAAGAATTAACAGTACAGTACATCTATGGAAAAGTATGTGCAGTTCATAGGTGCACAGCCTAGTCAACTTATCACAAAGTAACCAAATCGTTTCTTCACCTCCAGGCAAGGGACCATACCATCCCACCTCTCACGTCTCAGGAACGACCTGAGCCCCTTCTCCTCACTACTGTCTTCTCACCTAAAAGTAGCCGCTATTTTGAGTGCAAACGGATTTAATTTTGCGCTGCTTTAGAATTTATTACACAATCTTTCTTAAGCTTTTAAAAGTCAAAAATCTCTGGACAACCACTCCTTTCATGATATTCATCCAGAGTTGTAGCATAATCATTTTCTTTGTAGGAATCTCTGTCGGAACGGGAAAGCATGAATAGCTACTCCATAAATCATTAGCTAACAACTACTTATAATACATTGAGAAGTCAGTATGAAGAGTAACATTTAGCCTTTGTGGCAACACAATCCCCAAATGGAATTATAAAAATAAAGATAAATGAACATGGAATTTCAATTGATTAATTTCCATGGTTCTCTCTGTTTCCTGTCCTTCTCTCTCCTGATCGTAATGGGACTTTAGCCCTTGGGACCACAGCTTAACATCGCTTGATATTAAAAGGTTTTTCTCTCTACCTGTGGTTCTCTTGGTCTATGCATTTTTTCTCACCAAAAGTTGTATTGTTATAGTAAAAAAAGATAATGACTGTTCAAAGTCAACATATATGTAGAAAGGCTTATATGTGTGCAATTTCAAAATATGAGTGACCTCCATCTTACTTTAACCACAGTAGAGCATTATCTTTACTCAATTCCAAGGAGAAATCAGGCGTTTAAGAGGTCAATGACAAAGAAAAGGAAAAGGCTGCATTGTTATATATAACTTCTAGTGTAGGTACAATCAGTTTCTACGAAATGTTTGAAATGTGATGAAGGGAAAAGTCGCATCTTAGGATTCGGAATAGTGCATTTGCTGATGAACTCAGCCGGCGATTATGGTCCAGAGGTGGCTCTGATGTATTATAACTTTCCTCTAAAATACAAAAAATTAAAAAATAAAAAAGTTCAGGAATTCATTCTAGAAAAATTCATTGCGTCCGTCAGCTGTCCTACGAAGTAGGAGACGAGTTACCCTTAGACATAATGGGGCATTTTGTGCGTGCTGTCATTTTAGGAGTCTACAATAGCCCATCTTCCCTTCAAATGGTTATTGTCTACCATATTCCGTTCTACTATCGTAAAATCCCATCAACACCAAAACAACTCAAAGATATACATTGTGGAGATGATGCAATGTTAATTTGCTTCACTAAACAAAAGGGTTACTTTTCACTACCTAACCATCTTTATAACCTCACGTTGATACCTTAATAAATTACGCAGTAAAGTTAAAAATTTATTTTTAAAAACGAATCCCATCGTTTTTTTTTTTTTTTTTTTGTTGGATGGTGGTCTTCACTTTGTCACCAGGCTGGAGTGCAGTGGAGCGATCTCCGCTCACTGAAAAACTCCACCTCCTGGGTCAAGCGAAGAATCTCCATCTTTTGAATTAGTCTTGAACATCTATTTGTACAGATGTGGAAAAGTCAACTGGCTCCGGAATTCTCCTTCTAGCTTCTTGACTTCATTTGCATGTGGTACTATTGATATTGTTAGCAATTGTCTTCTTAAAAGCACATGATCCTTGACCTTTAGGGAATGAAGTGCTGGACAGATACCTCTAATTTCTGCAACGCCAATCGTCAGTTTATGAGAATGGAAACTCCCCCAAGGCTGGAGATTCTTGGTTAACCCGGATGGGCATTCACAAGAGTGTAGAAGAGGAAGCCCAACCGGCTGCGTAAGCTATTTCTTCACCTTGTATCTTAAGGTAGAGTACACCCTTTTGTGTGAATCTTTGGGCGGGTCAGTAAAATGCCTACTTATTTCAAATTGTTTCACTTGGCTTGCGTGAAAGTAGATGTATTTTTAGAGCAAGGTCATGTGGTAATTTTTTTAAAAATATACCAGCTTCTTTGATTATTTTCTTTTAAATTTTAAGCTTACTTTGAAAAATTCTTATTTTAAAGTCATAAGATTTTATAAAGTCTCTTACATAGAACTACCACTAGTCACTCCTATAAATTAGAAGCTAAACCATTCCCGTTGGGACATCGCTAGAAGAATGAATCTGGGAATTTGGAATTCAGAAAGAATCCCGGAGAAGTATTCTCTTCAGCTCGCTATTCTGTGTTGCTGGGTCTGGGAAACATCATTGACTTCTTCTGAGTCCAGTCTCTTCATAAAAAAAAAAATAAAAAAAAAAAAAATGGACGGGGGATCACCAGGTCTTCTTCTTTCTCAAAATGACATTAACTCTCAATCCGCTGCAAAGGGAAAACTATGTGGTTTCTTTTTATTTGTAAGCTAAAGTCCAAGCATGTTATGACAAAGGGTTCACCTGAAGTGCAAATGCCCTATTTATCCTAGGGTTCAGGACCCCTTCTTGAGAAGGGTTGTGATGAACCTGCCCCAAGCAATGGGTGGCCGAAGGCTTGCTGCTACTGCATCGTTCACGTCTACCTTAACTCGCCCTCTCTTTCGAGCCCCTTAGCTCACAAATCTGATCTAGAAGTCCACCTCCCATTCAGCATGAACTTCCTTATAAGAGCCCCTCAAAAAGTCACTCAGGTAAATCAACTCATGACATTCTTTTCAGCTAATATAACGTTCGCCTTAAAATGAAGCCCTCCAGGCCCTTCTAATTCTTGTCACACTTTCTCACTCCTCCAAAATAAACTGCTTTTCAGCTCAAGGAATTCTTAGAGAGAGAGGAAGAAGCACAAGTATCTGCCCGTTTGTAATGTTGTTCTACATTTTCTTTGCTCATTATTGTGTCACAGAGGTTAGCAGTATCTCCAGAGGCGGGGGTTGTGAGGAAAAAGTGTGAGGCTGTGGCCATTTAACTGTGAAAGTTCTTCCAGACGCTTGTTAATCGCTAATTTCCTAGTACTTGCAAATAGGACCTTAGTGGAACCTGCTACCATAGTGACGATTGACAAACCATGGCATACACCCACATTAGACACTCCCCTTTCCAGCTCCCCCTCTGGAATGAACCAGTAAAACAAATAATAAACACAAGCAAACTTATTCTTCTAGTGCTAAGTCTCTGTCTAGTATGGCAAAGCATACTGTATAGAAGGAAACTTGACCAGAGGAATACTCGGAGGAGCACCTTAAGTGAGTTTTGATTGCCCCACAGATTCTGAAAGCTGACATCAATCATTCTTCTATTTGTCCCACTACTGTAATATGAAGGTTAAGTGTTGCTTCAGGGAGCTTGGCATTGTTTCACTTCCAAGAATGAGGGGATGCTTATGCTCTATGACTTTCTCTTTCGCGGAATTGCCATTGCTTATCCATCCACAAACTGCTCCTGGTAATTTTTATACTAACAAACCATAACCTGATGGTTTCTCTGAAATAGAGGGTACTGTTAAGTCATCAGGATCCAGATATAGTGTGTGCTGAGGGATCAATTTTTCAAACATAATGAATATTGTAGTCCAGGAAAAGGTTGCTTGTGAAGCACTACGATGTGACAAAAAAAGATTGAGTCTAGGAACAATCACTTACTTTATGTACACTTCAATGTCTGCTCACCCAGTCTATTTGTGACAATTCATATTTCTGGACCTAATAAGGGTGAGCTAAGGAAGACAAACTGCAACATACCGAGTGAAAATCAATGATGTTTCCAACAAGCAACTCTCATTTCAAAGCACTTTCAAGATAAAAGCCATCCTCTTCCTAGAAAACTAAAGATGGGGGGCTGTTAATTCAGATGGAATCATCTGAAAGCTTTTTTCTTAGCACCAACTTACTTATTATTTTTTTAATCCCCCCCCCAATAAACCTAATAGTCACCTCTGGGTGAATAGTATTTTGAAAATGACAAAAAGAACCAAGATGTTAGAAGGCCCAAAGGTTAATTTGGAATGTGTTGAGATTTATGTGCTGTATTGTAACCTATTGGAATTGGGTGGCCGAAGCTGTTATAAGTTCTTGTGGGCATTATTTCAGGATTTTGAATTAGTGATTTAAATAAGTAAGAAAACTTCTATTGCGCTCAAGCCGTGATATAAAACCATGTCTAAATTCTTCAATAATTGAGTTTCGTCCAGAAAATCTCCGGTACTCACGATTTTATCAACGGTCCTCTTTTCGGGCCGCTGAGGACACTCCGACAAGTTGACTGGCTGTTTGGAAGGTTACTTCCCTTGCATTTTTCTCTCCTTCTCTTATCCTTTCTTTCTCATGTGCTCGCGTACTATCCCTCAAACTGGATCTTCCTCCCCTGTTCCTCTCCCCTCACCTTCATTGTTATAACCTAATTCCCTATGTTTGCATGTTCTTTCCTCTTCTGCCTCTCAAGTCATCAAGACCAATTCTTCATAGGTCCAATTTCTTCAGGGATTTCCTGGATTAGCCCTTCGCTACCTACGCGGTCTTCCTCAAAAACCCTTTGAAAGCCATGTTTATCCCGTAGAATAAAAGCAAAACACAACACGTAAGTTTGTTCTCTGTAGCAACAGATCCACAATGCAATTGACAATAGCGTTTATAATCTCTCATTTCTACCGTATATAAACTAAGATTATTAATGAGATATTCCATTTTTTCGAAAGTCTTGAAATCTGGAGTTATATTTATACTTTTCAAGCACATGTCAGTTGGACATAGCCATGCAAGTGTCATTGGGCGAGCTACTTACATCTGAACGTCGGGTCACGGCCTGCTGCCACATTTAGCTCTCAATCAGTACTTCCTCTCTTTCATATTTTCTACCAGACCAAATTGCAAAATACTCCGAGCGTTGTCGGCTACATTTTACAACCCCCCACTTCCTTCTGTCTCTCACATTTATCTCCGGTGGGTGTTAGAAGCATCATAATGCCTATTGATGGCTTGGAATATTAATTTTATTGGACCTCTTAAAACACAGCCTACTTACTCCTACTACCGTTCCTCAGGCTTTGTAAATTTCCCCTCCTTTCCCCATATTTATCAGATTCCTAACCAAATACCTCTGTTTGTTTAGATCTGAATCTCTGTGGGTATGTTGGATGTAGCTTATAAGAAATAGCTGTGCATTATTTGCAGTTCTAGATACGGGGGAGAGCAGCAGTTCAACAGAACACTCATCGTTCAGACATTCAAGAAAATAGGCTTAGAGCTGAAATCAAGATAATTGCCCCAGTGTTCTTCTTGCTTTCCTACACAGGAGAACCTGGCAACTTCGTAATGAGTCTCCGGTCACAGAATGTCCAACATGGAACGAGGCCTTTTCAATTGATTACAATGCAAAGACACTAGTTTTATCAGCTGAAACAAACACTGGGTAGGAATCGCTTTAAAATGCGGGAGGAAGGTCGTGAAGAAGAAAAAGGAAAACATGGAGAGTACAAGGACAGGATAGGGGACCGACTAAGTACTAGTGGACAAGAGAGGAGATACTTTACACAATCGTCGGGGTGCTAAAATGGGGGGAGCGTGCGAAAACAACTTATAACTGGGAAACCTCAGTTTAAAAAATATAGTTTACCACCCACTTCCATTTAACTCTAATTAATTCCTGTCCAGACACTCACCCCCCCCCCTCTGGCAATCCTGTATTTAATTGTTCTGCTGAGAAATTCTATGAGGGCTCAATCTTGAAACAAACACATGAATGGAGCCTGAGTGCAGGAATTCCCTGCTTCGCGAGGAGTCACTTCGACAGTCCACCACCAATGGCGTGATTCAAAATGAATACCAACTAGACATCCAGATGGCGGACTATGAGAGAACATGGAAAAAACTGAACCACACTAGAACCCGCCCATGGCACACCCCTCATGCATGCCTCCATACACAAGTTTTCTTGAACACTCTATGGTATAAATTAAATTAAGATGGTTTGTGACTTATGGTTTGCAACATCTTTTCTGGTTTGCTCGCTTCTCGAGTAAAATCGCTTTTTCTCTCGCACCAACCTTCTTTTCTCTTTTATGTCTAGCTTTAGAGCCAGCAAGCAGCTGAACCTGGTTTCTAGTTCCATCTACTCAACGCGTTACCATGCCACAGTTTTAGTAAACAAAATGCTGAGGGGTAATATTTCAAGCCTGTTATCATTTATTCCAAGGGAAACTCCCCAAAAACAGAATGATGGAAATTTGAAGTCTATGAACTTGCAATTAAACAAAACATCCTGATTCTATTTCATGCAATGAAATTGTCAAAAGTGTACAACTGGTATCTGCCAATACACAAATGTTAAGGACACTCTCTCAGTCTTTACGAGGAATCTGTCCAATTGGTTCCTTTATTAACTCAATAATTCATTATTAATTTTAAATGTGCTGGTTCGTTATTCAGTAAACACAATCTTAGCCCCATGTTAATTTCTTCTCATATAAATACCCACTCATTGTATTGTTACTAGGAATGGTTAACTGGTTTAATTTAATTTAAACGTTTGGTGCCCACTGCTAGTGGACTTTGACAGAACGCAATCTTACGATCACATCCAATTAATAAATAACAAAGGCATACATGAGCCCCACCGTGGCTCTGAAAAGGTCTTGTCGAATTTATCTGGGCCCTAGCTGTTGGAAATTCTTTACATTCTGCTGCCTGCTTTTTTTTCCTTCAGGACCATGCATTGTTGACCAGCACGGCCCCTTTTTTAGTCAAACAACATGACTGGCTCAACATTCTTCTGACTACTTAGCGGATGGGGACGCTCTATTCTACATAGTATTGTATGTTAGCTGACGCTTGGCATTCTAAAACGAACGCCCTCCCTCCCTCCACACACATCGTCACTGGAACTGGAAATTTACAGATGGAAGATGCTTCTTAGTAATTCTGCCCAGTTTGGAATAAGGTATCGTTTGCAGGGACGGGCCACTCTCAGCTTCTTTTTTCTATCTATGTTCTTCATTTTATTTATTTGAGCCTTTATTTAATTCTTACAGTTACATTAATAAAAACCATGGAATTGTATTTTGAATTCTGTTGCTTTTAACATTTCATTCTCTTAGAGTTTACTTTTTCTGCATTTACAAAAGTACTGTCTACAATGGGTCTGCGCACCGATAGTTCGAGGAAATACTAATTTTATCCTTGTGTATGGAGTCTATGCAAGAGATACCCCCAACTCCAACCTCTAGAATGGATTTGTAGGGAGAAAGGAAACACAGTAGCCCCAGAATGAAAAACCTCGGGGGAATGGGGGGAATTAATGGTTTCATGTAGATAAAAAATTGCTTGCCATTATTTAATTCTCGAGGTTGTTAGTCCCGTGCTGTTCACAACCTATCTTTCTACAAGCTTTATGGAATACATATATGTTAAGGCATGCTTGTGTCCGATAATATTATAGCCTTTATTTCTTGCTTGCCAGTGGTTCCAGTTTGCCTGCTGGTGTCATCAGGTAATATAACTTGGACAAGCCTTGTTAAAGGACTGTAGGTATGCCAGGCGTTCCTATTGGACAAACCCCTCTGAATAAATATCTCTGTGATGGCATCTTCAAAGTTGTATTCACAAGGTTTGTGTGATTGACGCTCTGGCTTGGTTATGGATAGCACTGCTTAGCCCCGTGGTCCATGCATTTACCTTGAAATTGAGGTACGTACACTGCACTGATTGGAATATAAAAAGTATTCAACTCAAAATCATTATTAATATACTCTACTTCAACCTTATGTATAAAAGAAGTTGTAGGTAGTGATGGATAATAATGCGTATTACTATAAGCACTCTACTCTCACCTAGCTGGACATGTTAATAGAT

**Supplemental data 5. Sequel read that did not extend through repeat, but contains approximately 30 repeats.** Defined repeat region highlighted in yellow.

Read name = m54120_170622_200608/18678009/5609_12485

Read length = 6,876bp

----------------------

Mapping = Primary @ MAPQ 40

Reference span = chr9:27,573,397-27,580,165 (-) = 6,769bp

Cigar = 5M1D4M1I5M1D4M1I3M1D2M1I2M1D1M...2M1D4M1D3M1I21M1I22M1D3M1D10M

Clipping = None

----------------------

H0 = 1

ZE = 0.0

ZF = 0.392487

NM = 1089

ZQ = 6876

ZR = 138394717

AS = 23351

Hidden tags: MD<hr>Location = chr9:27,573,611

Base = T @ QV 0

Alignment start position = chr9:27573397

CAGCCCCGGCCCCGGCCCCGGCCCCGGCCCCCCCGGCCCGGCCCCGGCCGCCCGTCCCCGGCCCCGCGCCCCACGGCCCCGGCCCCGCCCGGCCCGGCCCGGCCCCGGCCCGGCCCCGAGCCCCCCGGCCCCAGCCCCGGCCCCGGCCCCCCGCCCCGGCCCCGGCCCCGCGCCCCGGCCCCTAGGCGCGCGGACCTCCTGAGTCCAGAGCTTGCTACAGGGTGCGGTTTGTTGTTTCCCTCCTTTTTTCTTCTGGTTACTCTTTACTCAGGTCTTTCTTGTTTCCACCTCAGCGAGTACTGGAGATGCAAGTAGTGGGGAGAGAGCGGAGCGTGTGTAAAAACAAAAAACACAACCTCCTAAAACCCACCACCTGCTCTTGCTCGACCCCGCCCCCAAAAGAGTAGCAACCGGGCAGCAAGGGGGACGGCTGAACAACCAAGCTCATCTTTACGTGGGCGGAACTGTCGCTGTTTGTACGCACCTCTCTTCCTAGGCGGGACACCTGTAGGTTACGTCTGTCGTTTTATATGGGCGATTGGACGTTTACACGGGCATAGCGAAAGGGGCGGGGGCAACTGTCCTGTTCTTTTATCTTAAGACCCTGCTCTGGCTGGAGGCGTTGCGCAATAGCGTGTTCGAACCCCCCCCGTTTATAGGGGAGGGCTGCTGGATCCGGAGACAGTGAAGACGATTTCGTGTGGTTTTGAATGGTTTTGTTTTGTGGTAGGCAGTGGTCGCTCAACCAAATAATTGGTGGATGAAAATTTTGAATTTTGTTTTTACCGTAACAGACCACGTAAGTGCATTTCAAACTCCCCTGCAAACCCCCTGGTAGGGGAATGCTCGGCTGCGGCGGGAATTCCCACGGTCCCCTGCATCAGTCATCCGCAATTTGCCTTTACATGTAAGGAATTCTCTCAAGCATGAATTTGCACACTGGGAACTGTCCTTTGCTAGTTGCAATATGTGGTATGAGTTGTTTTTTTTTTAACTTTTGAAAAACGTATCCATTCTTGTTGATTGTGTAAAAAACATCAGATTTTGAAATAACCTTGCGGCGTTGGTGTCTGCAGTGTGTATAGTTCCACTTATACAGGATTGCGTAGCATACACCACTCTAGGATGTGTAAAAAAACAAAAGTTTTTAAAAACTGTGTGCCTTTGATCTGTCAAGTGTGAGCTGGCACTTCTTACAGTGATTAGCATTTAATCTTGTTCATCACTAAAAACACTACAGAACGTTTTTATCCTCAGCCGAGGAAGAAGAAAGGGAGGAATTAGGAATAAATACAAACCTGGCTCTCAACGTTCTATCACTTCTGCAGAAATCCCGAAGAAAAACAGCCTTCCTATCGTTCTATGCTTTGTTGAAAGTTGGACTCCTACAAATTGGGTCATTCTTTTATACCCAACTAACATCGTGGGGGTAGGGGGTAGAAAATGACTAGGCACAAATGACCACTGCTTCCCACATGGTATTCTTCCAACGTCCCAGCTGCAGTGCTAACTGCCCGTTGTTTGACAGCGACCGTTACTATAGTGCTAAAATGATCCGGCTGCAGCTTCATTGAGCGACACCATCAATCACCACGTGAAAAATCACCAGCTCCTCCGATCCTTCAAGATGCCAATGAATTTTCTCAAAGAGCCACTATGTGATACATTTCCTTTTTAGCAAAACCCTGCACCCCTTTCTTTGTTGTTGTGGATTATACCGAAAAGCCCATCTGATCTACAATGTAGCCCTGAATTGCAATCTTTTCTTTCCCAAATAAATCACTTTCTTACTTTAGAGATCTTATCTCTGGATTTTATTTTGACTGATGACAGCTTAGCACAGTAGCAGATTTACAAGTTTCTACCACTTGAGTGGTACTCTATATAAGTGGAATTAAAAAACAATGAATATAGAAACAGAGTCGACCCTTGCGTTGGGGGGCCCTTGGGGGAGGGAAAGAAAATTGTGGTTAGGGTACAAAGTTGTAGTAGTCTAATACATCTAGAGATTTAAGACTAAACATGAGGACTTACGCAGTTAATCATTGTGTGTTAGTCCTTCTTACACTGCTTAAAGAACCAACTGAAACTGGGTAATTTATAAAGAAGAAAAGTTTACTGGCTCAACAGTTCTGCAGGCTGTTGTACAAGAGCGGCTGGCTCAGCTTTCGGGCCAGCCACAGGGAACTTAAAATCCATGATTGCGAAAGGCCTGGGGACCCTCAAGACTTCAATTGGCAGGAACTGGGGAAGAGAGAATGCGCGAGGTGTACATTACGTTAAACAACTAGATCTTGTCAGCATCACTATCTAGTCCCAAGGGGGACTGTACAAAAACCCTAAATGCCTACCCCATAATCCACTCACCTCCCACATGGCCAACCTCAAACAACTGGGGATTACAGTTGACATAGATTGGGTGGGGACAGAATACTACCATGTTATTCCAACTCTGCCCCTCCCACTTCTATGTCCTCTCATATTGCAAAATACTGTCGTGCCTTACCAACCGTTCCCCAAAGTCTTAAACTCGACAGCATCATTCCAACAAAGCCAAAGTCCCAAGTCCACCTGAGATGAAGCTAGTCCCTTTACCTCTGAACCTGTAAATCAAAACACAAAGGTAATTGCTTCAAAGAATGATACAATGGGGTCTGGGTTGGTTATAGGCATGGCAGTACGCCATTCGAAATGGAGCAATCTGCCAAAAGAAAGGAGGGCTTATAGGGCCCCATGCACGTCTGAAAGCCAGCCGCCGGGCATTCTTAAATGTTTAAAGCTCTGAAATACTTCTCCTTTGACTCACACCCAGGCGAAACACTGATGCAATGAGTGGCTCCCAAAACCTTGGGCAGAACACCCCTGTGGTTTTCCAGTGTTCATCTTACCCACAGCCTGTCTCAGGGGCCTAGCATGGTGCTGCAGCTTTTTCCGGCTGCAGGGGCAAGTGTGGTGGATCTACCTTCTGGTGTTCTGGAGGAGCGGTGGCTCTCTGTAGAGCTTCTGAGACTGGCCGTGCCCCATGGGATCTCTGGGGGCTGCACCCCAATTTCCTTCTCCTTGCCTCCCTAGTAGATGTTCTCCATGCAGGATTCCACCACAGTAAACAGGTTCTGTCTAGGACAATCCCGGCTTTTTCTAGACATCCTCAAAATTAGGCAGCGCTTCTAAGCCTCACTCTGCCTTATTGTCGCCCGCCGGCTTCACAGCTTATGGGAAGCCACCCAGGCTTTTTATGCCGGTCACCCTGTGATAGCCGCTAGCCCTGAACGTCTATTCTTACTGGTGCAAGTTATCGAGTACCAGCTGCACATCCATTGGGGTCCTGCAGCAACTCAATTCTTGCCTCCTCAGAAGATAAAGGAATTTTACTCCAAGAGGCCATAATGGCAAGAAAAAGAGACCTGCGACAATGTTCAGCGCAGGAGTTGGTAAAGTTATTAAAAATCTTTCGAACAGGAATGAAAGGAACGTTAATTGGAAGAGGCCCACGTGGGCACCTTGGAGTCAATGTGCCTGTTGACCTTGAACCTAGGATCGATACACTGGCCTACTTTGACATCTTGTGCCCCTTTCCCTGGGCCCTCCCTAATGGTGAGCTTGCCGCATGCATGGTGCCTGCTTGCACTGGAAGGTGAGCGTTGCAGTGGGTTACTGGATGTATACATGTTACCTGAGGCTTTCTTTCCAATTCCGGGGAATGCCCCCAAAGGTCATCTCACCATTTTGCCGCTTAAGTGCAGGTTAAGCCACTTCTCAGTTGAAGATCTTATTGGAAGTCGGTCCCAGTTACCAATTTCAGGTGTTCATCTATTGAGAATTTGCCTCTCCTGGTTGCTGGCTGCAACAATTACTATTTTAGAGAGGTCAGTGACAATGTATGACCATCATCTGATGGTTGTGACATTGGTGGGTTGTTGGGGGACGTCTCTCTTACCCCACTCATGCCTGATTAGCTACCTGTTAACATACTGGGCCCCTTTGCGCCGCTGGGGATCAGGGAGCAGAGTCCCAAGCTGTAAGAGGGAGTCAGGGGCCCCTAGCTGTGCCAGGAAAGATTCAGCCTCCCGGCCTCTGGGCTGTGATGAGAGGGGACCACCCCGAAAGCCTAAAATGCCTATTAGGAACACTTCTTAACTGTTGGTGGGACTGTAAACTAGTTTCAAACCATTGTTGAAGTCAGTTGTTGTGGCGATTCCCAGGGATCTATGAAACTCGAAAATACCATTTGACCCAGCATCCCTATCCCGTGGGTATAACCCGCAAAAGGAACTATTAAAATCCTGCTGTGGCTATAAAGGACACCTGCACACATGTTATTTCGCACTGTTCACAATAGCAAAAAGACTTGAACCACACCACCTGCCAACAGTGATATACTGGCTTAAGAAAATGGGCACATATACACCATGGAATTACTTATGCAGCCATAAAAATGATGCGTTCATGTCCTTGTAGGACATGGCTGAAATGGAAAAGTCATTATCAGTAAATATTGCAAGGCTCAAAAAACCAAACACCACATGTTCTCACTCATAGGTGTGAATGAAAACATGACGCAACACATGGACACAGATAGGGGCACATCACACTCTGGGAACTGTGTGGGGTGGGGTAGGGGGGGGGGGGGGCGGGGGATATGCATAGGAGATATACCTAATGCTAAATGACGAGAATGGGTGCAGCACACCAGCATGGCACATGTATCAATGCAACACCGCACATGTGCACATGTACCCTAAAAACTTTTAAAGTATAAATAATAATAAAATAAAACATAAAATAAAATAAAATGCCTATTAGTCCTATGAGTCTTTCTCCCCATGGTTGGGTTATAGCCCTTGCCTTCTTTCCGGTTATGCACATTTATGCAGGCTGCTGACTCCTTCCTGAGAACGAGCCTTTTCTATACCCACAGGGCAGGCTGCCAAAATTACAAACTTTTCTGCTTGCTTCCCTTTTAAGGTAGTTCCAATTTCAGTCATTCTGTGGCTCATTGCCTATTGAGCATAGGCTATAGAAGCAGGCTAGGTCCTTCTTTAACACTCTGCTGCTTAGCATTTCTTCTGCCCAGAACCTAAATCATCATTCTAAGTCTTAATATTTCACAGATCCCTCGAACAGAGGGAACAATGCAGCTAAGCTCTTTGCTAAAGTCTAGCAAACTGACCTTACTCATTCCCAATAATTTCCTTCTTTCCATCTGAGACCTCTCCGCCTTGGACTTCACTGTCTCTCTCACTATCGTATTTGGTTGTCACCACTCAACACCGTTCCTCGCGAGTTCCAAACCTTTCTCTCATCTTTCTTGTCTTCTCTGAGCCCCTCTCAAGTTTTTTCAACTCGCCTGTTAGGCAGGTTCCAAGTTCACTCTACATTTTCAGGTATCTTTATAGCAATGCCCCTTCCTCTCAGTACCAAATTTCTGTTTAGCCATTCTTGCATTGCTATAAAGAAATACTGAGCTGGGTACTTTACAAAGAAAAGAGTTTTCATGACTCATGGTTCTGCAGGCTGCATCGGACGCATTTGCGCCTCACTTCTGGGTCTGCTCTCAGAGAAATTTCAATCATGGCGGAAGGCAAAGAGGGAGTGGCACTTCACATAGCGGGAGCCGGAGGAACCTAGCGAGGTGAAAGGGGGGAGGTGTTACACCCTTTTAACACCACCCTCTCTCGTTCGCACACTTACTCCCCCTACCTTCCCCCTCCCTGATGTGCGCACCCTAGACGCCCCCCGTGATCCAATCACCTCAACCAGGCTCCATCTCCCAACGTGGGGATTGAACTTAACATGAGATTGGATGGGGATTGATCAAACCATATTATCTTTTGTATACTGGAATATTGGTAAGAGATGATTTTAGATAAATTTTATCCCATACATCAAAGGTGTATGAAATTGATGGATATGTTAATTTGTGGGACGGTATTCTACTATTTAAACGTGATTCCAACATTATGTTGCCCATTTTAAATACCTACAATTAAAAAACATTACACTATCCTCTGACCTCTCTAGGAGTGATTTCTTGTCCTCCCTAGGATGTAGATATGTTTTACAGAGGAGATAACTGGATCTCCTGAGCGCTTGGAGATATTACCCCAGACATACTTTGAAAATTCGGCCAGTTTTAGTTCATGCATTCTAGAAGCTCACTGCGTTCCAGAGGACAAACAACCTGGACCATGTTGATGATAAACTCATCTTCGCTACACTCCTATGGTGTTAATACTAGCCTCCCCAGTTTAGAGTAAGACTGGAACTTTGATAAGTGCCTGGGAGGGGGAATACAACGCCAAAAGCAGTTTCCTACTTCAACATTTGGCCTACAGACACATAATTTCCTTTTCTCGCATAGGTTTTTCTGTTTTTTTTTGTGATAATCATACCCTGAAACACTAACTATGTTTCCTAAGAGAGAAATAATCAACACCAATAGCTAAATCTACCTGCTTCATGAGAAATAAAATATATGGGAAAATGGCTCTGTATGCATGCTGCTTTTAGCCATGACTGTGTATTCTCCCCCCCACCTAATCCCAAAGGTTATTTAGTCATGTATTTGTGTAGCCGTTATAGTAGCTTGTAGTTTTCTTAGATTGCGAGCAAAAAAAAAATCAGGAGAGAAACTTCTCATTAGACACATAGTACAGTGGTCCAAAAACTTTATTGCCTGAAAAGGGTTTTATAAAGAGGAGGACTAATAAAATTCTAGCCTATATGCAGTCCTCTGGGAACCAAAGAGAAAAATGATCTTTAAGTATCTAGTAATGTGATTCCTATTAGCAGCATGAAAGAAGAGGCTTGGGGGCTTACCCCCTTATCCTTTTCCTACTCTTGTTAACTGGTTTATAAAACACTCAGTAACTGTAG

**Supplemental data 6. Sequel read covering approximately 69 repeats.** Defined repeat region highlighted in yellow. The ‘GACCAC’ highlighted in red partially matches the defined anchor, and may signify the end of the repeat region at approximately 66 repeats, though the adjacent sequence does not unambiguously match the rest of the anchor.

Read name = m54120_170621_235746/63046380/15882_26004

Read length = 10,122bp

----------------------

Mapping = Primary @ MAPQ 40

Reference span = chr9:27,573,155-27,581,952 (-) = 8,798bp

Cigar = 7M1D9M1I4M1I9M1I8M2I3M2I4M2I...1M2I2M1D3M1I6M1D4M1D5M1I4M1D7M

Clipping = None

----------------------

Location = chr9:27,573,568

Base = C @ QV 0

----------------------

H0 = 1

ZE = 0.0

ZF = 0.359151

NM = 2304

ZQ = 10122

ZR = 138394717

AS = 24992

-------------------

Alignment start position = chr9:27573155

GGAACCGGCCCGGCAAGACCACAGGCTCCGGCCCCGGCCCGGCCACGGCCCGGCCGGCCCCGCGCCCCGCCCCGGCCCCCCGGCCCCGGCCACCGGCCCCGGCCCCGGGGCACCGGCCCCGGAACAGGACAAAGGCCAGGACCCGGCCCCCCCCACCCACCAGGAAAAGAGCCCGCGGCCCTTGGCACAACGGCACCCAGGCCCCCACCTGGACCCCGGGCCCCGGCCCCGCCACTGGGGACACAGGACCCGGCCAAGGCCCCAGGACACGGACCGGGCCACGGCACCGGGGCCACCGGGCCCAGGCCCGGCCCCGGCCAACGGCCACCCGGCACACGGCCCGGCACCGGCAAGGCCCACGGCCCCGGCCCCAGGCCCCGGCCAACGGACCCGGCCCCCGGCCGGGCCCGGCCACCGGACCCCTTAGGAGCGCAGACTCTGAGTTCCAGCCGCTTGCTAACAGGGCTGCGGTTGTTTTTTCCATCATTGTTTTCTTCTGGTTAATCTTTTATCCCGTCTTTTTTGTTCACCCTCCGGCGACGTTTACTGTGGGAGCAAGTAGTGGGGAGGGGAGAGGGGGGAAAAGAAAAAAAAAAAAACACCAAAAAAACACACACCTCCTAACAACCCCATGCTTCTTGCTAGAAACACCGCCCCCAAAAAGAGCGCACGCAACCGGGCAGGGGCAGTGACGGCTGACACACCAAAGCGTCATCTTTTACGTGGGCGGAAACTTGTCGCTGGTTTTGACGCACTCTCTTTCCTAGCGGGACACCGTAGGGGTTACCGTCTGTTCTTTTTTCTATGTGCGATTGGGGACGTTTTCTCACGAGGGGCTTAGCGAAATGGGGGCGGGGCAAATTGTCCTGTCTTTTATCTTAAAGGACCCGCTCTGGAGGAAGGGCGTTGGCGCATTAGCGTGTGCGAACTTAATAGGGGAGCTGCTGGGGAGTCGGTGCATCCGGGGGGAGAAAAATGAAAGACGATTTCGGGTTTTGAAGGTTTTGTTTGTTGCTGGTAGGCAGTGGGCGCTCAAACAAAAATTTGGTGGATTTTGAAGAAATTTGTCTTTTACCGTAAATTAAGGATTAAGCCACTGTAAGTGCACTTTCAAGAACTCACTTGCAAACCCTGGTAGGGGACAAGCCCGCGCACTGCGGCGCGGGAATTCCACGGGTCCCCTGCAAAAGTCCATCCCAATTTTGCCTTACATGTAAAGCATAGAATAAGGAATTCTCTCAAGGCATGATTTTTTCACACTGGGGGAATGTCATTTTTGCTAGTGTGCACTATGTGGGTATGATTGTTGTTTTTTTTACTTTGAAAAAAAACCGTACCATTCTGTTTTGATGTGTAAAAAAACACCAAAGATTTTTGAAAACTTGGCGGTCTTTTGGTTCTGCAAGGTGTTAATAGATTCTTCTTACTACAGATGAGTAGCATTTACACCACTCAGCTGTGAAAAAAAAAAAACAAGGTTTTTTAAACTGCTGTGCCTTTTTTTTTACTTGCAAGTGTGGAGCTGGCACTTATTACAGTGGAGTAGCATTTACTCTTTTTCATCAATAACAATCACACAGAACGTTTTAATCATTCACGAGGGCAACGGGAAGAAAGGGGAGGAAAATAAATAAATACACAAAATGGCTCTCAACGTCCACCCTTCTCGCAGAAAACGAAACCGACCCTTTCTACTGTTCTTATGCTTTGTGAAAAGTTGATCATACAACAAATTGGGTCATTCTTTTTATACCCAACTAAAATAGTTGGGGTCGGGGTTAAGAGGAAAGGAAAAAGCACTTAGGGACAACTGCCCACTGCTTTCCACAGTGTGTGTGTAATTCTCTCCAAGTCGGCTCCAGCTGGCTGACAACTGCCCGTTGCTGACCTGGAGGAACCAGTTTTATCTACTTATCTAATAGTGCTTTTAAACATAATGGACACATGCTGCCAGCTCTAATTTTTTTTTATCTACCAACCATCCCCATTCACCAGTTTGAAACTCACCCATCCTCAGATCCTTAATAGTGCCACAATGAATTTCTCAAAGGAGCACTATTTAACCTTTCTCTTTTTCAGCAAAAACTCCCCTTCTCATTTTCTTTGTTTGTGTTGGATTTTCTTACCGACAGGAACATCTGATCTCATGTTATGCCCTAAATTGCACTTCTTTCTTCCCCAAAATAAATCACTTAATTAGGAGGATTCATCTCTGTTTTTATTTTTGGATGACCGCTTATAAAACCAAGTAAAGGCTAGGCATTTTTACCAAGTTCTACACTGGAGTTGTAATTCCACCTTATAACGTGGGAATTAAAAAAACAACGATTTATAGAAAACGAGTAGACCCCTTGGGTGGGGGGCTTGGGGGGAAAGGGAAAAATTGTAGGGTAGTGTACCAAAGTTTGCAGTTAGTCTATCTAATACTCTAGAGATTTAATGTACACCACAGAGGATAGCTGTACATAATTGTGTTAGTCCCATTATTCTTACACTGTTATAAAGGCAAAGAAAAATACTGGAAAGGACAAAACTGGGTATAAATTTATAAAAGAAAAGTTTAATCGGCTCACAGTTCTGGGGGCAGGCTGTAACAAAGAATGCAGGCTGATCAGCTTCTGGGCAGCACACAAGGAACTTAAAAACTCAATGATGGACAGGCATAGGGAGGGACCCCCATGAACTTTCACCATGGGCAGGAAACTTGGGGGAAAGAGAAGAAGAAACTGGGAGGCTGACTAACATACGTTTTTTTTTTTAAAACAAATAGATTGTCAGACTCCCTATACTAGTACCTAAGAGGGGAATGTACAAAACCATTCGAAGCCACCCCCATAATCCACTCAACCTCCCACCAGGGCCCAACCTCCAAACCATTGGGGATTACAGTTGAAACCATGGAGATTTGGGTGGGGACAGAGATCCCAAAAACCATGTTTTCCAACTCTGGTCCCCTTCCCAAATCTAACTGTTCCTTCTCATTATTTGGCAAAATACTGTCGTTGCGGCCTTACTACAACAGTTCCCCAAAAGTCTTACTCGATCTCCAGCATTTCATTCAAAAAGTTCCACAAAAGTTCCCACGAGTTCACCTGAGACGAAGTAGTCCCTTCTACCTATTGAACCTGTTAAAATCAAAAAACAAAGGTAAATTGGCTTCAACAGATACAAATGGGGGGTTCTAGGCATTGGGAGATCTGCAATCCGAAAATTCCGAAAGGGGGCGAACGGCAAATCTCCAAAGGGAAGAGAAGGCTTTAATTAGTGGCCCCATTGCCAAGTTTCTGAAAGCCAGCCGTGGGCAGTCTCCTTAAATGTTTTTTCCAAGCTCTGAAAATAATCTCCTTTGACTTCACACCCCAGGCAACACTGGATTGTCCAATGATGTGGGGCTCCCAAAACTGGCAGGAACCACCCCTGTGGGTTTTTTCCAGGGTTCATCTCCCACGCTGCTCTCATGGGGCTAGCATTGATGCTTGCAGGCTTTTCCAGGGCTGCAGGGGTGCAAAGTTTGTGGTGATACCATTCTTGGGGTCTGGAGTGAGGTGGTCTCTTTTTCTTTGTCATAGCTCTGCTAGGCAGTGCCCCCCCAGGGGGAACCTCCGTGTGGGGGCTGGCCTGCAACCCACATGTTCTTCTCCTTTGCTTTCCCTATTAGAGTCTCCATGAGAATTGCCACCCCCCAGGGAGAGGAAACAGGGGGCGGCGGGACCATCCAGGGGCGGGGCAGTAATCCCTCCTAAAAATTTCTAGGCAGACGCTTCTTACGCCTAACTCTTCGCATTATGTGCGCACGCCGGCTTCACCGCTTATGGAAAAGCCACCACGGCTTATTGCCTGGCACCTTCTGTGAAGCAGCCAGGCCTGAATGTATTCTACTGTGTTGAAAGTTTATCGAGTTAACCGCTGCCAAATCCATGATGGGTTCTTGCCAGGCAACCTCAAATTCTTGCCTCCTCAGAAGAAATAACATTGACCAAGAGGGCATAAGGCCAGAACAGGAGACAAAAAGCGGGGATGCGACAAGTTTCAGGAGCAGGAGGTAAAAGTTTTTAAAAAGCTTTAGACAGGAAATGAAGGAAAAAAGACATTTGGGAAAGAGGGCCCAAGTGGGACCTTGGAGGTCAACAGTGCCCTTGTTTTGACTTGAAACTAGGATCTTATTACACTGGACCTACTTTGACATATTGTGCCACTTTACATTGGTCCTTCCATAAAGGGAGGCTTTTTGCCGCACTGCATCGGTGTCGCCCTGGGATTGCCACTTGAAGGTTTGAGCTTGTTGTGGCAAAGTGGTTTAACTGGAGTTGTATACTGCGGGGGCTTGACCTAGGCTTTTTCCCTTTTCGGTGGAATGCCCCCAAAGGTTTCATATTCACACATTTTGCCTTTCTTCATGTGCATGTTAAAGGCACTCTCTCAGTTCCTGAGATCTTATTGGAAGCGCCCACAGTTACCAATTTTCAGGTGTTTCTTATCTATTCGAGACAGTTTGCCTCTCCCCTGGTGCTGGCTGCAACCAATATAATTTTAGAGAGGGCAGTATGACCAAACTGCCTTGACAATAATCTCGATGGTCTGGCCTGGACATTCCTGTGTGGGTTGGTGGGGAACTTCTCTCTTACCCCACTCATCGCTGATTAGCTACCCTACTGTAAACCAGTACCTGGGCCCCTTTGAGCAGCTGGGGGGAAAATTCGGGAGCAGGCTGTCCCAAGGCTTGTGTAAAAATGTTAATGAGGAGAGCAAGGGGCCCTAGGCTGGCCCAGGCAAATTGATTCAGTCCTCCTAGCTCGGGGCCTGTGTATGAGAGGGGGACCCCCATTGCAGCCTTTTTAAAAATTGCCTATTAGGAACACTTTTTACCACTGTGGGGACCTGCTAAAACTCGTTCAAAACCTTTTTGGGAAGTCAGTGTTGGCGATCCTCTCAGGGATCTAGACACTAGAATACCATTTGACCCCAGCCATCCCATCACTGGTATATACCAAAGGACTATAAAAATCCTGATGCTATAAAGACACATTGCAACACTATGGTTTATTGTGGGCATGTTCACAAATAGCAAAAAGACTTGGAACCAAACCAATGTCCAACCGTGAATTGGATAGACTGGATTTATAAGAAAATGGGACATATACACCATGGAAATACTATGCAGCACATTAACAAAAAAATGATTGGGGGGGTGGGGTTTTTTGAGTTCATGTCCTTTGTATGTGACAATGGATGAAATGAAATTCGTTTTTCTTCTCTCAGGGATATAAAAAACTATTTGCAAAGGTCAAAAAAAAAACACAAACACCACATGGTTTCGAACTAATTACGGTGGGAATTGAACAATGAGAAACACATGGACACAGGAAAGGGGAACATCACACTTCTGGGAATGTTTTGGGGTGTGGGTGGGGGGGAGGTGGGGAGAGCATTAGGAGGATTATACACTGGCTTAAATGAAGAGTTGTTAATGGTGCAGCACACCAGGCATGACACCTGGTATACATTATGCAAAAAAAACTTAAAAGCTGCAACGTGCGAACCACATGTAACCTAAAAACTTTTAAATGTATAATAATAAATAAAATAAAAATAAAAATAAAACTAAACTTAAATGGACTATAGTCCTATGCGTTTTTCTCCCCATTGTTTTGGTTTATCAGCATGCCTTCACTTTTAGTTATGCAAAATTTATGCACGCCTGCTTGAACTTCTCTCTTGAGAAACGAGCTTTTTTACTACCACATGGCCAGGCTGCAAAAATTTACAAACCTTTTATGGGCTCCTTTTGTCTTCCCTTTTACGTGTTAAGTTCAATTTCAGGTCATTCTGTGCTCCAGCCTATGAGCGCATAAGGTAATTCGAAGCAGCTAAGGTTATTTTGAACATCTGCTGCTCTTAGAAATTTCTTCTTGCCAGATACCCTAAATTCATTCATTCTTAGTCTAAGAATTTCATACAGATCCCTTAACACAGAGGACAATGCAAGCTAAGGCTCTTTGCTAAAAAAGCCTTAGCAAAAACTGACCGTACTCATTGCCAGAAATTAAAGTTCTCATTTCCATCTGAGTGACCTCCTCAGCCTGGACTTCACTGGTCATCCCACTCTCAGTATTTTGGTTGACAAAACCATCAAACCAGTTTCCTAAAGTTCCTAGCGAGGGTTCCAAAACTTTCTTCACTTTCTGTTCTTCTTTGAAGCCCTCTCAAAATGTTTCAATTCTGCCTGTTAGCCAGGGTTCCACAGCACTTCTACATTTTCAGGTATCTTTATAGCACTGGCCCTTCTTCTCCGTAACAATTTTCTGTATTAGTCCATTCTTGCATTGCTATAAAGAAAAGAAATAACCTTGAGTGACTGGGTATTTTTCAAGAAAAAAAGAGGTTTTAATTGCCTCCTGGTTTTCTGCAGGGCTGTATAGGAAGCATGGCAGCAATTCCGCTTTTTTTCTGGGGATTGCATCAGAGGACACTTACAATCATTGGCGGAAGGCCAAGCGGGAGCTGGCACTTCCACATTTAGCGGGGAGCAGGGGAGGAAACAGCGAGAGGGAACAGGGGGGGGAGGTGTTACACACTTTTAAACAAACAGCTTCTGTGAGAACTTACCTCCCTATACCGTACCCCGACACGGATTTTGGTTCCCTGCGCGTTCCTTTCCCTGCCTCTTCCCTTTTTCTTCTCCCCTCCCTCTCCGACCACGTGATGGTGCTTTGAACCCTTAGCAGCCACCCGCTTCTGTGCTCCGCTCACCTCCCCTCCACCCGGCCTCCATCTCCACGTTGGGCTTGCAAAGGTCTGAACATGAGCTTTTCTTTGGGATGGGGTATGTGGGCTCAACCATATCGATATTGTCTACTGTGACCATTGGTAATTTTAAGCAGGACTATCTTTTAGGAGATATCTTATCACATACATAAAAAGGTTAGCTATGACAATGATGGGTATATGTTAATTGTTGTGCTTGGACTGTAATATAATCACTATACCACGTGTTAGATCAAAAATCATTATGTTGGCCATTTAAATAATACAATTCAACAAAAAAGTTACGACTTAATCCTGCCTGACATCTGTCTGGGATGTATATATTGTCGTCCCTAGGAATCGTTGTAGATATGTTTACAGAGGAGGGACAACTGGGACCTCTTGTCGAGGTTGAATTATTTTACCCAACGGCTACATAACTCGTAAATTAGGCCAGTTTTCATGCAATTCTAAGCGGCTCATGGAGTCCAGAGTGGACAACCAAACTGGTACCAAGTTGATGATCACTCCTCTCAGATAAAATCCTCTGTTGGTTATTTAATACTAGCATCCCAGTTTAGAGTACGATGGAACACTTGGATATTACGTGCCGGCGAGGGGGAATACAACTTCCAAAAAAAGGCAGGTTTTCCCTTACTTCAAAATTTCGGGGCCTAACAGACAAAAATAATTTCCTTTTCTCACAAAATCGGTTTTTCTGTTTTTTTGTGATATCACCCTGAAAACAACTATGTTTCCTAACGAGAGATAGCAAATACTCCAACCACCAAAGCTAAAATTTTACATGATCATGAGAACATAAACTATTTTATGGGTGGTGTTGGGAATAATAGAACTGGCTCTGTGTATGCCTTGATGCTTCTTAGCCAGTGGACTGTGTACTCTTTCCCCCCACCTAATTCCACACAAGTAATTTAGTCCATTGTTGTAAGCAGTTTATAGTAGCTTGATTTGTATGCTCTTTTCATAGATTGGAGCAAAAAAAAAATTCAGGGGAGAGAAAAAGGAGAACAATAATTATTAACATAGTACAGTGGTCCATAACTATCTTTATTGCCTTGAAAGGGTTTAAAAAGAGGATGGCCAACATATTCTAGCATATATGCGTCTTCTGGCGAAAACAAACAAAAGATGAGAAAATGATATTTAAGTATCTAGTAATTGTGGATTCTATTAGCAAAGCCAATTGAAACGATGGTTGGGAGGGCTTTAAAAAACGTATCCTTTTCATTCCTCTCGTTAATCGGCTTTAATTAAACACCCAATCAGTACTGTAGCATTTGCTCTAAATTGAATAGGTTTTTTCTACAAGAAATATGTGTCTTTCCAAGTGAGAAAGCCTAGTCCTCCATTTTCAGACCCTGTTAATGACGTATCCCTTTGCCTTTACTTCTTAATTTTTTAAAAAAAGACCTTTATATAGAAAATTTTTGAACTCTTCAACATATAATTAATGCAGTCATCACAAAAAGCTCTAACAAAATAGGGAAGCAGAAAGAAAAAAATAGAATATGGTGTTTTTAAGAGCAGTGGGGCTCTGGAAATCGAGGGAGTATGGTGTGAGTATTGTTTCTTTGCCTTTAGATTAGTGTGTTATGACATTTCTTTGGGCCATGTTTAAGTACTGTATTTCTCTGCTGCCTAGTTTTCGCCCAGCTACCAGAAATCGGCATGCAGAATAAATTTTCCTAGGTTCATTTGTGAGGGTCATGTGTGATAAAGCAAAGCTAAAGGATAAGGATTTTCGAACAAGTCAAATTCGAGAAGTGGGTTCTAAATCTCCATTTTACTAACTATGTGCAGATGAGATATAAAAGGCAATAACAAATGGGGACTTCTTAGCGCTTTTAAACTATGTACCTATGTATGATTCCAATAACTGATTCTATCAGAATCCATATAAACCTTCTGCTGTGTTCAAAAAATTGCTCAGTTGTTGTCAGGAAGGCATTCTCGGCTATTATTGTATTTCAAAAAAATGCAATATCTTGCTGCTGCATCTCAGCGCTAGACGCTTCAGAGAGTTGTTGATTGGATTAGCTCCCATTTACTCCAGATGCTACAGCAAGAATTACGATTTAATGTGACTGGAATGTGAAAGATCTTAGGTAGGGGAAACATGGAGGAGGAATACCGCTCCTCACCATAGACACCATAGTGCCATTAATTTTCTTTTTTCTTTTTTTTCTTTTTGAGGATCAAGGAGTTTCACTTCTTGTTGTTCCAAGCTGGAAGTGCCATGGCACGATCTTCAATCCTCCCACCTTTCCCTGGGGGTTTCAAGGTGACTTCTCTGCCTCGACTCCTGACGTAGCCCGGGGATTATTAAGGCATTGCACCACCATGGCCCCCTGGGTATGGTGTTAGTTTTGAGTAGACGGATGGGCTTTCTCCCATGGTTGATTCATGCGGGTCTCAAAACTCCCAACTCAGGTGATCTGTCCCTCCTTGGCCTCCCGCGTTTGTATTCACTCGAGGTGGCTGAGAGGATACAGGTTGTGAGCCTCCCCGTGGCCTGACTTATAAAATTCTTTTAAATACGAAGATTTCTTATTGTGGTTGCCGTCAATCATAAGATAGGCGGTATGCACTTTATGGTCCATTATCTTTTTTGTTGGGAATGTGAATGGAACCAACACCTACCTGAGGGTACTTCTAGGTGGAAATCTGATTCTTGTATTATTAAGTTTCACTTTGGTTGCCCGTGTGATATAGATGGTTTACCACACCACTCCTAGGGGGTTTCGTGTTGCCTGGAGCCCAAACATCTACAGGGTTCGTGATGGATTAGCCGATGTAAAACAATGCCCTTGCAAGAGGGGTACATCATCAGGGGCCGTTGCTGTGGTGTGGCTGGGCTCCACTGCGTCTGCCTGCAATCCAGCACACCTGGGAGGCCGAGGCTGGTGGATTCATTTGAGGTCAGGAGGCTGCAGCCGAAACACCAGCGGACCGCAGTGTGATATGGGCAGAGTTCGCCCTTCCCCATCAGGCCTTGCACGGTGGCAAGGAGCCAAGGCTGGGAGCCTTGTTTCCTGCTTACATGAACCACCTACTGGGGCTAACTTTTGCTCTTTGACAGGGATCATTTCTGACAGCATGCCGATGTAGTAGAGCCTGGCTGTTGGTCCAGGCCTCGATCGTCAGCACAAGTGGTGGAATCGGTCAGTGAGAAGCGTTGTTTCTGTGTTGACCACTGTATGCCAGTATTCGTTTTCTACTGCTGATCTACTAAATCCCCACCATATTTATGGCTAAAGCCACCGCTTCCTATTTATTCGTATCCCGTTTTTGCTAGGTGTCAGAACGCTTTCCAGCCCCTTAGTGATATGGCTGACTTATCTGTCAGGGTATTCA

**Supplemental data 7. Sequel read that did not extend through the repeat, but contains approximately 912 repeats.** Defined repeat region highlighted in yellow.

Read name = m54120_170621_235746/39387429/0_26180

Read length = 26,180bp

----------------------

Mapping = Primary @ MAPQ 40

Reference span = chr9:27,573,581-27,593,881 (-) = 20,301bp

Cigar = 5506S7M1D15M1D5M1I6M1I6M1D8M1D...10M1I7M1D1M1I4M1I13M1D13M3I1M

Clipping = Left 5,506 soft

----------------------

Location = chr9:27,573,582

Base = G @ QV 0

----------------------

H0 = 1

ZE = 0.0

ZF = 0.365217

NM = 2800

ZQ = 26180

ZR = 138394717

AS = 74827

-------------------

Alignment start position = chr9:27573581

CCCCGGCCCCGCCCGGCCCCGGCCCCGGCCCCGGCCCGGCCCCCGGCCCCGCCCCCGGCCACGCCCCGGCGCCACGGGCCCCGGCCCCGGCCCGGCCCCGGCCCGGCTCCGGCACCGGCACCGGCCCCGGCACGGCCCCGGCCCCGGCCCCGGCCCCGGCCCCCGGCCGCCCCGGCCCCGGCCCGGACGCGCCCCGCCCCGGCCCCGGCCCCGGCCCCGGCCCCGGCCCCGGCCCCGGCCCGGCCCCGGCCCCGGCCCCGGCCCGGGCACGGCCCCGGCCCCGGGCCCCGGCCCCGGCCCGGCCCCGGCCCCGGCCCCGGCCCCGGCCCGGCCCGGCCCAGGCCCCGCGCCCGGCCCGGCCCCGGCCCCGGCTCCGGCCCGGCCCGGCCCGCCCGGCCCCTGCCCCGGCCCCGGGCCCGGCCCGGACCTCGGCCCGGCCCGGCCCGGCCCAGGCCCCGGCCCCGGCCCCGGCCCCGGCCCGGCCCGCCCCGGCCCCGGCCCCGGCCCCGGCCCATGCCCGGCCCCGCCCCGCGCCCGGCCGGCCACGGCCCCGGCTCCCCGCGCCCGGCCCTGGCCCCCCGGCCCCGGCCCGGCCCCGGCCCAGGGCCCGGCACGCCCCGCCCCGGCCCCGCCACGGCCCCGGCCCCGGGCCCCGGCCCGGCCCGGCCCCGGCCCGGCCCCGGCCCCGCGCCCCGCCACGGCCCCGGCCCCGGCCCCGGCCACGGCCCGGCCCGGCCCGGCCCCGCCCCGCCCGGCCCCGCCACGCCCCTGCCCCGGCCCGGCCACGGGCCCGGTCCCGGCCGGCCCCGGCCCCCGGCACCCGGCACGGCCCCGCCCCGCCCCCGGCCCGGCCTCCGGCCGTCCCCGGCCCCGCCCCGGCCCCCGGCCCCGGCCCGGCACGGCCCCGGCCCCGCCCCGGCCCGGCCCCGGCCCCGGCCCGGCACGGCCCCGACCACGGCCCCCCGGCCCGGCACGGCCCCGGCCCCGGCCAGCCCCGGCCGGCCCGGCCCCGGCCCGGCCCGGCCCCGGCCCGGCCCGGCCCGGCCCCGGCCCCGGACCCGCGCCCGGCCCGTCCCCGGCCCGGCCCCGGCCCGGCCCGGCCCCGGCCCGGCCCTGCCCCGGCCCGCCCCGGCCCCGGCCCCGGCCCGGCCCAGGCCCCGGCCCGGCCCGGCCCGGCCCCCGGCCCCGGCCCCGGCCCGGCCCCTGCCCCCGGGCCCGTCCCGGCCCGCCCGGCCCGGCCGGCCCCGGCCCGGCCCGGCCCCGGCCCCGGCCCCTGCCCCGGACCCCGGCCCCGGCCCCGGCCCCCGGCACGGCCCCGCCCCGCCCAGCGCCCGGCCCCGGCCCCGGCCCCGGGCCCGGCCCCTGCCCCGGCCCCTAGGCCCCCTGGCCCCGGCCCCGGCCCCGGCCCCGGCCCGGCCCAGGCCCCGGACCGGCCCCGGCCCCGCCCGGCCCCGGCCCCGGCCCGGCCCGCCCGGCCCCGGCCCCGGCCCCGGCCCCGGCCCCGGCCGGCCCCGGCCCCGGCCCGGCCCCGGCCCGGACCGCACCGCCCGGCCCAGGCCCCGGCCCCTGCCCCGGCCCCGGCCACGGGCCCCGCCCCCGGCCAAGGCCCAGGCCCCGGCCCGGCCCGGACACGGCCCCGGCCCGGCCACCGGGCCCGGCCCGCCCCCGGCCCCGGCCCCGGCCCCGGCCCCGGCCCCGGCCCGGCCCCCGGCCCCGGCCCCCGCCCGGCCCCGGCCGGCCCCCGGCCACGGCCGGCCCGGCCGGCCCCGGCCCCGGCCCCGGCTCCCGGCCCGCCCGGCCCCGGCCCGGCCCCGGCCCGGCCCCGGCCCCCGGGCCCCGGCCATCGGCCACGGCCCCGCCCCGGCCCCGGGCCCGGGCCCCGGCCCGCCCCGGCCCCGGCCCCGGCCCCGGCCCCGCGCTCCCGGCCCCGGCCACGCCCCGACCCGGCACCGGCCGGCCCCGGCCCCGGGCCCCTGGCCCCGGCCCGCACCGGCCCCGGCCCCGGCCCCGGCCCGTCCCCGGCCCCGGCCCCGGCCCCGGCCCCGGCCCCAGCCCCGGCCCCGGCCCCGGCCCGGCCCGGCCCGGCCCCGCGCCCGGCACCGGCCCCGGCACCGGCCACGGCCGGACCCGGCCCCGGCCCCGCCACGGCCCCCGGCACGTGGCCCAGGACCCGGCACCGGCCCGGCCCCGGCCCCGCCCCGGCCCCGGCCCCGGCCCCGGCCCGGCCCCGCCCAGGCCCCGGCCCGGACACCGGCCCGCCCCGGCCCCGTGCACCGCCCCGGCCCGGGCCCGACCGGCCCCGGCCCGGCCCCGGGCCCCGGCCCCGGCCCCGGCCCCGGCCCCGGCCCGCCCCGGCCCGGCCCGGCCCCGGCCCCGCCCGGCACCGGCCCCGCCCGGCCCCGGCCCCGGCCCCGGCCCCCGGCCCCGGCCACGGCCCCGGCCCGGCCCCGGCCCCGGCCCCGGCCCGGCCCCCCGGCCCGGCCCGGCCCCTGGTCCCCGGGCCCCGGCCCTGCCCCGGCCCCGCCTCCGGCCCGCCCCGGCCCCGGCCCGGCCCGGCCCGGCCCCGGCCCCGGCCCCGGCCCGGCCCCGGCCCCGGCCACGCCCCGGCCCCGGCCCCGGACCCGGGCACCGCGGCCCCGGACCCCGGCCCCGGCCCGGCCCCGGCCCCGCCCCGGCCCCGGCCCCGCCCCGCCCTGCCCCGGCCACGCCCCGGCCCCCGGCCCTGGCCCCGGCCCGGACCCGGCCCGGCCCCGGCCCGGCCGGCCCCGCCCCGGCACCGGCCCCGGCCCCGGCCCCGGCCCCGGCCCCGGCCCGGCCCGTCCCCGGCGCCCCGCCCCCGGCCAGCCCCGGGCCGGCCACGGCCCCGGCCCCGGCCCCGGCCCGGCCCCGCCCCGGCCCCGGCCCCGGCCCGGCCCGCCCCCGGGGGACCCCGGCCCCGGCCCGGCCCCCGGCCCCGCCGCCGCTGGCCCCGCCCGGGCCCCGGCCCGGGCCCGGCCCGGCCCCGGCCCCGGCCCCCGGCCGGCCCCGGCCCTGGCACGGCCCGGCCCCGGCCCCGCCCCGGCCCCGCGCCCGGCCCCGGCCGGCCCCCGGCCCGGCCCCGGCCCCGGCCCCGGCCCCGGCCCCGGCCCCGGCCCGGCCCCGGCCCCGGCCCCGGCCCCGGCCCCGGCCCGGCCCCGGCCCCGGCCCCGGCCCCGGCCCCGGCCCCGGCCCCGGCCCCGGCCCCGGCCCCGGCCCGGCCCCGGCCCCGGCCCCGGCCCCTGCCCCGGCCCCGGCCCCGGCCCCGGCCCCGGCCCCGGCCCCGGCCCCCTGCCCGCCCGGCCCCGGCCCGGCCCGGCACCCTCGGCCCGGCCCCGGCCCCGGCCAGGCCCCGGCCCCGGCCCCGGCCCCGGCCCGGCCCCGGCCCCCGGCCCGGCCCGGCCCGGGCCCCGGCCCCGCCCCGGCCCGGCACGCCCCGCCCGGCCCCGTGGCCGGCCCCGGCCCCGGCCCGGCCCCCGGACCCGGGCCCCGCGCCCGGCCCGCCCGGCCCCGGCACCGGCCCCGGCCCGGCCCCGGCCCCGGCCCCGGCCCCGGCCCCGGCCCCGGCCCCGGCCCCGTCCCCGGGCCCGGCCCCGGCCCCCCGCCCCGGCCCCGGCCGGCCCCCTGCCCCGTCCCCGGCCCCGGCCCGGCCCCCCCGGCCCCCGGCCCCGGCCACGGCCCCGGCCCGGCCCGGCCCCGGCCCCGCCGGCCCGGCCCCGGCCCCGGCCCCGCCCGCCCGCGCCCCCGGCCCCGGCCCGGCCCCCGGCCCCTGCCCCGGCCCCGGCTCCCGGCCCCGGCCCCGGACCCCGGCCCCTGCCCCGGCCCGGCCCCGGCCCCGGCCCCGGCCCGGCCCCGGCCCCGGCCCCGGCCCCGGCCCCGCCCCGCCCCGTGCCCCCGGCCCCGCCCCGGCCCGGCCCCGGCCCGCCCCCGGCCCGGCCCCGGCCCTGCCCCGGCCCGGCCCCGGCCCGGCCCGGTCCGGCCCCGGGCCCCGGCCCCGGCCCCGGCCCCGGACCCGGCCCCGGCCCGGCCCCGGCCCCGGCCCCGGCCCGGCCCGGCCCGGCCCCGGCCCCGGCCCGGCCCGGGCCCCGGACCCGCCCCGGCCCGGCCCCGGCCCCGGCCCCGGCCCCGGCCCCGGCCCCGGCCCCGGCCCCGGCCCGGCCCCGGCCCCGGCCCCGCCCGGCCCCGGCCCCGCGCCCCGGCCCCGGCCCGCGACACCGGCCCCGGCCCCTGGCCCCTTGCCCGGCCCCGGCCCCGGCCCGGCCCCGCCCCGTCCCGGCCCCGGCCCCGGCCCCGGCCCGGCCCCGTCCCGGCCCCGGCCCCGGCCCCGGCCCCTGCCCCGGCCCCGGGCCCGGCCCCGGCCCCGGCCCGGACAGGCCCCGGCCCCGTCCCGGCCCGGCCCCGGCCCCGGCCCCGGCCCCGGCCCGGCCCGGCCCGGCCCCGGCCCCGGACCCGCCCCGGCCCCGGCCCCGGCCCCGGCCCCGGCCCGCCCCGGCCCCGGTCCCCCGGCCCCGCCCCGGCCCGCCCGGCCCCGGCCCGGCCGGCCCCGGCCCCGCCCCGGGCCCGGCCCCGGCCCGGCCCGGCCCCGGCCCCGGCCCCGGTCCCCGTCCCTGCCCCGGCCCCGGGCCCCGGCCCCGGCCCCGGCCCCGGCCCCGGCCCCGGCACGGGCACCCGGCCCCGGCCCCGGCCCCGCCCGGACCGGCCCCGGCCCCGGCCCGCCCCGGCCCCGGCCCGGGGCCCCTGCCCCGGCCCGGCCCCGGCCCCGGCCCGGCCCCGGCCCCGGCACCGTCCCGGCCCCGGCCCGGCCCGGCCGGCCCCCCCGGCCCGGCCGGCCCCGGCCCCGGCCCGCCCGGCCCCGGCCCCGGCCCCGGGCCCCGGCCCCGGCCCCGGCCCGCCCCTGCCCCGGCCCCGGCCCGGCCCCGGCCGGCCCGGGGCCCCGGCCCCGGCCACGGCCCGCGCCCCGGCCCGGCCCCGGCCCCGGCCCCGCCCCGGCCCCGGCCCCGGCCCGGCCCCGGCCCCGGGCCCCGGCCCCGGCCCCGGCCCCGGCCCCGGCCCCGGCCCGGCCCCGCCCCGGCCCCGGCCCCGGCCCCCCCCCGGGCCCCCCCGGCCCCGGCCCCGGCCCCGGCCCCGGCCCCGGCCCCGGCCCCGGCCCGGCCCGGCCCCGGGCCCGGCCCGGCCCCGGCCCCGGCCCCGGCCCCGGCCCCCGGGCCCCGGCCCCGGGGGCCCCGGCCCGGCCCCGGCTCCCGGCCCCGGCCCGGCCCTCGTCCCCGCCCCGGCCCGGCCCGTCCCAGGCCCCGGCCCCGGCCCCGGCCCGGCCCCCGGCCCGGGCCCGGCCCCGCCCCGGCCCTGCCCCGGCCCCGGCCCCGGCCCCGGCCCGCCCCGGCCCGGCCCCGGCCCCGGCCCCGGCCCCGGCCCCGGCCCCGCGCCCCGGCCCGGCCCGGCCCCGGCCCCGGCCCCGGCCCTAGCGCGCGACTCTGAGTTCCAGAGCCTGCTAAGGCTGCGTTGTTTCCCTCCTTTTTTCTTTCTGGTTTAATCTTATCAGGCTTTCTTGTTCACCCTCAGCGGAGTACTTTGAGAGCAAGTACGTGGTGAGAGAGGGTGGGGAAAAAAAACAAAAACACACACCTCCTAAACACACACATGCTCTTGCTAGACCCCGCCCCCAAAAGAGAACGGCAACCGGGCAGCAGGGCGGCTGACAACCAAGCGTCATTCTTTTCACGTGGGCGGACCTCTGTCGCTGTTTGACGCACCTCTCTTTTCCTGCGGGACACCGTAGGTACGTTGTCTGTTTTCTATCGTGCGTGAGTTCGTTTTCTCACGAGGCTAGCAAATGGGGCGGGGCAACTTGTCTTTTCTTTGATCTTAGCCCCGCTCTGGAGGACGTTGGCGCAATAGGTGTGCGAACCTTAATAGGGGAGGCTTGCTGGCTCGTGAGGAAATTGAAGGCCGATTTCGTGGTTTGAATTGTTTTGTTTGTGCTTTGTAGCAGTGGGCGCCTCAAACCAAATTGTGGATGAAATTTTGTTTTTTACCGTAAGACCACTGTTAAGTGCATTCAAAACTCACTGCAACCTGGTGGGGACGCTCCGCGCACTGCGGGCGGGAATCCCACTGTCCCCTGCAAAGTCATCGCATATTTTGCCTTCACATGTAAGAATTTATCTCAAAGCATGATTTTCACACTGGGAAGTCATTTTTCTAGTTGCAATAGGGATGGTTGTTTTTTAACTTTTTGAAAAACGTACCATTCTGTTTGATGTGTAAAAAACAAAAAGATTTTTGGAAACCTTGCGTTCTTTTTGTCTGCAGGTGTATAGAGTCCCTTACTACAGATGAGTCGCATTTACACCATCAGATGTGTAAAACACCACAGTTTTTAAACTGTGTGCCTTTTGATCTGCAAAGTGTGAGATGGCACTATTTACAGTGAGTAGCATTACTCTTTTGTCATTCACTTTAAAAATCAGCACCGACACGTTTTAATCATTCACCGAGGAAAGGAAAGGGGGAGGAATTAAATACACAACATGGCTCTCAACGGTACACCTTTCTGCAGAAACAGACCCTTTTCTACTGTTCTATGCTTTGTGAAAGCGGATCATACAAATTGGGGCATTCTTTTTATACCCAAACTAAAACTAGTGGGGGTAGGGGTTCGAACAGCACTTAGGACAAATGACCCTGCTCCCACCGTGAATTCTCTCCAAGTCCAGCTGCGCAAATGCTCCTGTTGTGACCTGAGAACAGTTTTATTAATAGTTGCAAAATGACCTGCTTGCGGCTCTAAATTTTATTCTACCACCATTCACTCACCAGTTTGTAAACCACCGCTCCTCAGACTCTTAATAGGCCAATGAATTTTCTCAAACGAGCACTTATGTAACATTTCTTCTTTTTAGCAAAACCTCCCCCTTTTCTTGTTTGTGTTGATTTACGAAGACCCTCTGATCTCACATGTTGCCCTAATTGCCAATCGTTCTTCCCAAATAAATCACTAATTTAGAGATTCACTCTGTATTTTTATTTGCCTGACAGCTTATCCCAAGTAGCTTAGCATTTACCAAGTTTCTACCACTGAGTTGTACTTCACTTCTACGTGGAATTATATACAAACAATGAATTTATCGAAACAGAGTAGACCCCTGGTGGGGGGCTTGGGGGAAAGAAAAATTGTGGTTATATGGTAAAAGTTTTGCCGTTACGTCTCTACATCTAGAGATTTGTTAAAACATGAGGACTAGCGTAATAAGTGTGTTAGTCCATTCTTACCCTGCTATAAGAAATAACTGAAAACTGGGTAATTATAAAACACGTTAATTGCCAATCAGGTCTTGCAGGCTGTACAAGACCGCATGGCTGATCAGCTTCTGGGCAGGCCACAGGAACTTAAAAAATCCTGATGGAAGGCATCGGGCGACCCCGGACTTTCACATGGCAGGAATGGGGGAAGCGGAAATGGGGAGGTGCTACATACGTTTTTAAAAAACTAGATCTTTGTCCGAACTCACTATATAGTACCAAGAGGGGACTGTACATAAACCAATTAGAAGCCACCCCTAATCACTCACCTCCCACCCGGCCCAACCTCCCAACACGGGGATTACAGTTGAAACATGCGATTTGTGTGGGACAGGATCAAAACCAGTTATTCCAATCTGGCCCCTACCAAATCTAATGTCCTTCATATTGCCAAAATCCTGTGTGCCTTACCAAAGTTCCCCAAAAGTCTAACTCGATCCAGCATTCATTCAAACGTCCAAAGTCCCAAGTCTCACCTGAATGAGACGAAGCTCCGTCCATTCACCTATGAACCTGTAATAATCAAAACAGGTAATGCTTCAAAGATACAAGGGTTTCTAGGCATTGGGCAGATACTGCCATTCCGAAAGGTGAGAAAGAAATCTGCCAAAAGAAGAGGGGCTATCGGGCCCCATTGCAAGTCTGAAAACGCCAGGCCTGTGGCAGTCATTAAATGTTAAAGCTCTGAAATATTCCCCTTTGACTCACACCCAGGGAACATCTGCTGCAATGGTGGGCTCCAAAACCTTGGGCAGAACACCCCTGTGTTTCCAGGGTTTTCAAATCCCCACAGCTGCCTCTAAGGCTCGCATTGATGCTTGCAGCTTTTCCAGGCTCAGCGGTTGCATAAGTTGTGGTGTCTTGGATGGATCTACCATTCTGGGGGTATGGAGGCGGGTGGCTTCTCTTGTCCTAGCTCTGTAGGCAGTGCCCCAGGGGACTCTCTGTGGGGGCTGCAACCCCACCTTTCTTCTCTTGTTCCCAGTAGATGTCTCCCTGAGGATTCCACCCCAGTCACCGCTTGTCTGGACCATCCAGGCTTTTTATACTCCTCTAAAATCTAGGCAGCGCTTCTTAAGCCTCCCCCAACTCTTGCATTATGTGCCGCCGCCGGCTTCACAGCTGATTGGGGCAGCCACCAGGCTTATGCCTGGCACCCTGTGAGCCGCAGCCTGAACTGTATTCTTACTGGTGAAAGTTATCTGAGTTACCAGCTGCAAATCCATGGTGTCTGCCGAACTCAATCTTGCCTCCTCAGAAGAAAGATTTGACCAAGAGGCATAGGCAGAAAAAGAGACTGCGACAAGTTCAGGCAGGAGTAAAGTTTATAAAAAGCTTCTAGAAACAGGAATGAGGGAAAAGTACATTTGGAAGAGGCCCAAGTGGGGCACCTTGGATGTTCAAGTTCCTGTTTGACCTTGAACTGGATCTTATACCACTGGCCTATTTGCCAATCTTGTGTCCCCTTTCCCTCGTCTTCCCTAAGGCGTGCGTGTGCGCATTGCTGTTTTGCCCTGCTTGAACTTGGAAGGTGAGGATGTGCAGGTGTTTACTGGAAAGGTTTGTATACATGCTACTGAGGCTTCTTCCCTTTTCCGGTGGATGCCCCCAAGGTCATACTTCACCATTGCCTCTTAATGTGCATGTTAATAAGCCCACTCTCTCGTTCCTGAGAATCTTATGGAAAGCGCCCAGTAGTTACCAAATTTCAGTTGTTTCTATCTCTTGAGACGTTGCCTCTCCCTGGTGCTGGCCTGCAACCAATACTATTTTATAGAGGCAGTATGACACAATGCCTGCACCATTCATCTGATGGTTGCCTGATCATTCCTGGTGGGTGGTGGGGACTCCTCTCTACCCCCCACTCATGCCGATTAGCTACCTACTGTACAGTACCCCCCATGGGCCCCGTTAGCCGCTGGGATCAGCGGAGCAGAGTCCAAGGCTGTACCGAGGGAGCAGGGGCCCTAGGTCTGGCCCAGGAAATGATCAGTCCTCCTCAGGCCCCTCGGGCCTGTGATGAGAGGGACACCATGTGAGAAGCCTAAAATGCCTATTAGGCCACTTTACACTGTTGTGGGACTGTATAAAACTAGTTCAAACCATTGTGAAGTCAGTTGTGGGCGATTCCTCAGGGATCTAGAACTTAGAAACACCATTTGACCCCCCAGCCATCCATCACTGGGTCTTTATACCCACGAATCTAAATCCTGCTGACTCTAAAGACACATGACACTATGTTTATGTGGCACTGTTCACAATAGCACAGAATTTGGAACAACCCCAAATTGTCCCACAAGTGATAGACTGGATTAAGAAAATGTGGGCCACATATACACCATTGAAATACTATGCAGCCTAAAAATGATGAGTTTCATGTCCTTGTTCGGACATGGATGAATTGGAAATCGCATTTCAGTAAACTATTGCAAGGTCAACAAACCAAAACCACATGTTCTCACTCATAGGTGGAGGAATGAAAATGAGAACAAATGGACACAGGAAGGGGACCATCACACTCTGGGGACTGTTGTGGGGTGGGGGAGGGGGGAGGATAGCATTAGGAGGATTATACTTAATTGCTAAATGCGAGTTAATGGGTGCAGCACCCCAGCTGGCCACATGTATACCTATGCAAACTAACCTGCACATTGTGACATGACTAAACTTTAAAGTTTAATAATTAATAAAACAATTTTAAAAAATAAAAATAAAATAAAATAAATGCCTCTTAGTCTATGAGTCTTTCTCCATTGTTTTGGTTATAGCCCTTGCCTTCCTTTTCGTTCTGCAAATTTATGCAGCCTGCTTGACTCCTCTCCTGAGAACGAGGCTTTTCTTTACTACCACACATTGGCCAGGCTGCAAATACAAAAACTTTTCTGTCTGCTTCCCTTTTTAAAGTGTAAGTTCCAATTTCAGGTCATTCTGTGCTCATGCCTAGAGCACGGCTTTTAGAAGCCGCTAGGTTACTTCTTGAACACTCGCCTTGCTTAGAGCAAATTTCTTCTGCCAGATACCCTAAATCATCATTTTAAGTTAAGACTTTCACCAGATTCCCTAGAAACAGCGAGGAACATGCCAGCTACGCTCTTTGCTAAAGCAAGCAACCTTGACCTTACTCATTTCCCATAAAATTTCTCATTTCCATCTTGAGACCTCCTCAGCCGGACTTCACTGTCTATCTCACTATCAGTATTTTGGTTACAACCACTCAACAAGTTCCCTACGCGATGTTCCAAACTTTCTCCATCTTTCTGTTCTTTCTGAGCCCTCTAAACTGTTTCAAACTTCTGCCTGTTAGCATGTTCCAACGTCACTTCTCCATTTATTTTGTATCCTTTTCTTTTCTTTAGCAAATTGCCCTTCTTCTCAAGTAACAATTTTTGTTATTAGTCCATTCTTGCCTGCTATTAAAGAAATACTAGCTGGGTAATTTACAAAGAAAAAGAGGTTTAATTGACTCATGTCGCAGGCGTATAGGAAGCATGGCAGCATCAGTTCTGGGGATTGCCTCAGAGAACTTACAATCATGGCGGCAGGCAAAGAGGGAGCTGGCACTTCCATAGCCGGGAGTAGGGGACCAGGAGGAGAGAGGGACAGGGGGGGAGGTGTTGTTACACATTTTTTTTTAAACAACCAGATCTCGTGGAGGACTTACTCACTATACAGACCCAAGAAGTAGGTGCTGACCATTAGAAGCCACCCCTGTGATCCAATCACTCCAACCAGGCTCCCTCTCCAACGTTGGGTGGATTGCAAGCTGAACATGAGATTTGGATGGGGATATGGATCAAACCCATATTCAAGATGATCTGGCAATTGGTAAGCGAATCGATTTAATTAATCTTTCCCCTACATAAAAGGTCGCTATGACATGATGACGATTGTTACTTTGCTTGACTGTCGTAATCACTATACACGTGTATATCACCAACATTCTGTGCCCATTTTTAAATAACCTACAATTAAAAAAATTACACTTAATCCTCTCTGACAATCTCTAGGATGTAGAATCTGTCATCCCTTAGATGTCGAATGTTTTTACAGAGGAGATTAAACTGGAACCTCTGAGAGCTTGACTATTTACCACGGCTACATTACTTTGACAAATTAGGCCAGTTTCATGCAAAAAAAAATTCTAGAGCTCACTGAGTCCAGAGGACAACAACCCTGTTACCAAGTTGCATGCTAACTCATCTCAGATAACTCTATGGTGGTGAATATCTAGCAATCCCCCGTTTAGAGTAGACTGAACTTGGGATTTCGTGCCGGGGAGGGTGAGAAACAACTCCAAAAGCAGGTTTCTACTCTCAAAATTTGGCCCCCTACAGACAAATAATTTTCCTTTCTCGAATAGGTTTTTCTGTTTTTTGTGATATCAACCCTGAAACAACTGTTTCCTAAGAGAGAAATAATCAACACAAAGCCTAACCTCTACATGATCAGAGAAAAAACAATATATGGTGAACATGGCTCTGTATGCATGATGCTTCTAGCCAGTGGACTCTGTTATCTCCCCCCACCTCATCCAACAGGTATTTAGTCATGGTCGCAGACATAGTAGCTTGATGTCTTTCTGATTGGAGCAAAACTCAGGGGAGGAGAAACAATAATTATAACATAGTACAGTGGTCCAAAAACTTCTTGCCGAAAGGGAAAAGAGGGAGGACCCAAATTCTCGATTATATTGCAGTCTTCGGGCACCAACGAGAACTGATTATTTAAGTATCTAGTAATGTGATTCCTTAGCAGCATGAAAGATGGCTGGGCAGGGCTTTAACCCTCTCCTTTTCATACTCTGTTAACTGGTTTTCTAACCACATCAAGTAACCTGTAGCATCTAAATGAAAAATAGGTTTTTTCAGAAATTGTCTCAATGTGAGAAAGCTATAGTCCTCCATTTTCAACAGAATGAACCATTAAAAGACTTACTAGAAATTTTTGAACTTCTTCACATTTAATTAATGCAGTATTACCAAAAGGACTCTACAACTAGGAAGCAGACCAGCTATGGTTAAGAGAGGGGCTCTGGAATCAGAGCTGGTTGGAGTATTGTCTTTGCCTTTGATGAGTTATGACATTTTGGAGTACTGTATTTCGCTGTTGCCCTCAGTTCCCAGCAGCTACACAATGGCATGCAGCATAATTCCCCTAAGGATCATTTGTGAGGGTCAGTGTGATCAAGCCAGCTAAGGATTAGAACAGTACATCGAGGAAGTGGTTTCTCATCTAATTTTACTAACTATGCAGAGATAATAAAAGCCATAACAAATGACTTATTAGCCTTTTATCTTTTTAAACTATGTACCTATGTATGATTACAATAACTATTATATCAGAATCCAGAACCTTTGGTGTCAAAAATGGCATTGTTGTCAGGCAGACTTCTCGCTATAGTGTATTTTCAAAAATGCAATATCTGCTGCTTGATCATAGCTAGAGCTAGAGCTTGTGATTAGCTACCATTTATCAGATTACCGAAATTAGATTTAACTTGAGGCAGTGAAAGATCCTTATGTAGGATCATGGAGGAGGAAACCGCCTCCATAAACCAAGTGCATTTAATTTCTTTTTCTTTTTTCTTTTTTGAGACAGAGTTTCACTCTTGTTGTCCCAGGCGGAGTGCAAGGCACGCTCTCAACCTCACCTCCTGGGTTCAAGGATCCTCCTGCCTCAGCCTCCGGGAGTAGCTGGGCTTATTGGCATGCAACCCACCATGCCCCGCTAATTGTATTTTTTCGTAAGAGGGATCTTTGTCTCCATGTTGATCAGGGCTGTCTCAAACTCCAACCTCAGGTTGATTGCCCTCCTTGGCTCCCGAAGTGCGAGCTTACAGGTGAAGCCACCGTGCCGGACCTTAATTTTCTTTTTAAATAATGAGATTTTATGTGGTTGCCTCCATCATTACAGACAGGCGGTATTGCACGTTATGGTACATTATCTTTTGGTTGGCCGGTGAATGAAACCATACACCTACCTGAGGTACTTTCTAGGTGAACTCTTGATGCTCGATATATTAAAGTTACTCGGGTGCCAGTGTGATATTAAATGCCGTTTACAAACACTCTATGGGTTCTGTGCCTGGAGCCAAACTCTACAGTTCTGATGATAGAACAATGTAAACAATGGCCCTTTGCAAGTAGAAATCAGGCCGGGTGTGGTGGCCAAGCTGACAATCCCAGCACCCACTGGGAGGCCGAGGCCTGGTTGGATCATTGATGGTCAGGAGTTCAACCCCCAGCCTGACTAACATTGGTGAAACCCCTATCTCTACTAAAAATACAAAAAAAATTAGCCGATGTGGTGGCACATGCGCATCCCATCTATTGGAAGGCTGAGGCAGGAGACAGGCGAGGAGAGTCCCTTGAACCCGGGGGGGGCGGTTGCAGGAGTGAGGCTATGCCACTGCACCCAGCCGGGCAATGGATTGGGGGGGAAAAGAAAAAAGAAAAAAAAGAGAAATAAGTAGGATCCAATCCTCTTTCCTTTTGGTTTTCCTGGGACTTATGATTCTAAATAATAAGCTAATGAACTTTCAACAACGAGAATCATCATCTTTGCCAGACATTAACCTCCCTATTCCTTCTCTGTTGATTCTCTGCACTTTATAAGCACACAGGTTTTGAGTGCTCTAAGTCCTGGAATACAGAGTTCTGGGGAGAGAGGAAGTTCATAGTTATTGCAGCCTCAGGAAAACAACAGACGAAAGGAGGAACAAGACGTAGAAGATGAGCACGATCTTCTTAAATTTAGAGAAGACCACTAGCTTATAGAGAAGGAAGAGAGGAATTTGAACTAGTACCTTGATAGGTGGCATAATTGATGGAATTTAGATGGAAAAGAAACAGTGCCAATGCTGGTTTTGTACTGACCTTCCATATGCCTGAGTAGGGGGGGGGACTAGTAGCAGTGAGGCCTCAGCACTCCTCACAGCAATGTCCCCTTTTAGACATACTTACCAAATCACTTCAGAATGGTCCCTGAGCAACCGGCCTAGCTTTACCCCCTCTCTTGCAGCTTCAGCATTTATTTTGCATTGTATAGTTGTATTTATTGTAGTTACTGTATTACTCGTGCTTGTAAGTAGTTTCTTTAGTTATTTCAGATGATATGGAATACCTGCTTCTCACCTATGTTCACATGCTTTTGAGACCCGGACTGTAGGTTGACAGTTTTATTTGTTAAAATCCTTATGTTGAGCTCATCTCAGTATGCTTGCAAATGCCCGTAGCTGATTGACCTTCACTCAACTACCCCAGAAATTCTTATCCTGAGTCATGCTAGTTTTAACTTCATGATGTATTTGGAAAAACCCTTACTTAAATATTCTTATTGGTTGTAAATTTAACAAAAACCCCAAACCAAACAAAACAAGAAGCAACCAAAACACAAAGACACACGTCCTACCAAAAGTTACTAAATCATTTCACTCTCATGTTCTTCACCTTTCAGGGGCTTCCCATTTTATATAGTTCAAGGTCCTTGGCATGACTCTCTAGTTTTTCCCAATCTGGCTCCATTGACTTTCAAGTTCTCTCTTACCGCCCCTTTCCTTTGATCTTGCGCCGCATGACTGTTTACTGCTCTGTAAACCTCTCCATATTACTCTCTCTGCCTGAAATGTACTCACCTCTCCATTTGTGTGTGAGCAAACCTTTATAACTGCCGCCATATGTGCCTCCTCCATGAAGTCTTCCTGGATTTTTCCCAGTTTGAATAATTCCATGGCACCTTTAAACCTCCACTAGTAATACAAAAACCAAACTTTTGTATTTCCCATTTACATTTTGGGGGCTGAAATACTGTACTATATATTAATTAGAACTAATGAATTTGAAACTATTAGCGTTCGTTGCAGTGACCACTAATTAGGGTTTTTAAAGGCATGACGTTTTGGAGTCAAAACCAACCTAGTCTTTACTTCGGCTCAATCCACTTTGCATCCTTGGGGGATTAGTTATAAATTTTTGAGCCTCCAATTTCCCCTTTGAAAATGGAAATTGCTGCGGATTTAAATGAGGTTACCATCTCAACGGGTTTACTTGATAACCTGCCCCATGGTAATGCTCAGAGTAAGTTTAGCTACTATTATCACATGGTAGACGTCAATTTGTGTTTGTTATAAATGATTCCCAACCCTAGTTTAAGATGAAGAGATGAAAGGGAAAGGAAAGAGATTTACTCGAGGTTACACCTTTAGTTGGCGGCAGATCTGTAATATAACACTTTCATATGCCAGCAACCCAGGTAAGAGCTTTTTTTTTTTTTAAATCAACATCAGGTGTGTTCTACGTCTTTCTCATCACTAGCTGAAAGCTTCGGAGGGCAGGGAACTTGTCTGATTCACTTTCGCTGCGCTTTTCCTCTAAGCACCGTAATTGCTATGTTTAGGTCTTAATAAATGTGGATTGTACTTTAATACGAACGTTTCCCATGAACAAAGAAAGAAAGTTTCCCATTAGAAAACAAGAATAAATAAACTGTATTTAATCAATGGTAGATTAAAATTTCCATAAGGCCTCTAAATCTGGCACACAAATCAAGTGCTGTTAATAAAGGGAAAAGCTTAATTGAACATTATTGCATTTTTAGGTGACAAACAACAGGCCACTTCATCATCAAGATTCTAGAAAGAGCCGTAGCGTTATTTCCTATCATTGCCCAAGCACAAAAAGAATCTAAGAGATTCAAACACATATTTTAACCTGCAGTGACACCCACGGCATTTAACATCAGTTTAAAATTTCAATACAACAATATATAAGTATTTGCCTTACGGAATAATGGAATTTTGACAAGTTATTACGTGAAGGCTTATTATCCCCTGTGAGAAAAAACTGCGTCAGGTCGTAAGTCTGCTTACAAAAACCTAAGAAGGGATGGATTGTCATGTTTATTGTTTTTCATCTCAATATGTAGAGAGATAGTCACCTTAAATAGCTTGCCGGAAACATGACAGAATGTCGATTGTGTCCTATTGTCAGATTGTGCAAAGTTCACCCTATAAAAAGTAGAGACACAGAGACAGCACAAATAATTTAGTACAGAAACCAACAACTAATGGAGAATGGAGGTTATACTTCAAATTATAGTATTAACTCCCTCAAAATTATTTTCTTATTGGAAATGATTTGTTTGCCTGTCCATATTCCATGTGCCGGATTGGATATAAAAAGGAATAGTGCTCACAACTATTTGCTGATCACCTCTGACGAACTATCAGTTGTTTTTTTACAAATTAGGCTTTAGAAACGAAATGAAGGATGCGCATGCGGAAATGACATAGATCTACTCTCAAGCACTTCGCCATTAATGAACTATACATTATAAATTCAGGAGACACGCACCTTTGTTTTCGGACACAGTTGGCAAAAAAGAAAAGAAAAGAAGTGGTCATTGATAAAGCGCAGATACCCAACACCCTTCTAGTTTTTGCTACACAAAGATCCAATTATCTCTCATCAGTTCTCGAAGTTTTTACAACCTGCCTCTCCTCTTTATTCAGGAATAAATTTTTTTTAAAAAGCAAATTAAACAAAAACGAACAAAAAATTGCAGGTTCAGATAACTCTTAAATGCCTAAATCCTGTCCTTGTGCGAAAAAACACATAAAAAGGCAGTTCATCAAATCAGCTTATCTAGGGTAGCTTTTTATACCATAGTCCAGCTCAGTTTGCCACGTGTTTGTTTTAAAATTAATATCACACACACGCAGCTTTCACTCTCTACGTAGAACTCCCAGAAAATGTTGGAGGTGAGAAATGCAGATGGGGTTATGAAGTAATTTTGTACTCTAAGGACAGATTTTTTTTTTTAATTCACATTACTTCATGTTGAGAAGATCATATTATGTATTAAAGCAGAAACACGCCCCAAAAAGCCACCCTTCCAGTCTCACTGTCACTTTCTGCATACAAAACACCACTAAATTTTCCAAATGAGTAGGCTAATTCTTTCCTTAACAGAGCCAAAAAACCTTGCTTGGAGTTGCTAAACGTAGGTGACAGCACTTTGTTCAAACCCCTCAATTCTTTTTTTTTTTTTCCGAGAAAGATTTCACTCCTCACTCTGTTGCCCAGGAGTTAGAGTGCAGTGGTGTGATCTCGATTATTGCAGCTTGGCCCTCCGGTTCAAGGCTTCTCCACTCAGTCTCCAGATGTAGCTGTGGACTATTAGGCGCTGTCCCCACACGGGCTAATTTTTGATTTTTCGTATGAGATGGGGGTGGTTTCACCACGTTGGCCCCGCTTGTCTTGAACTCCGGCCTCAAGTGATCTGCTCACCTTGGCATCCCAAAGTGCTGAGATTACAGGCATGAGCCACGTGTGGGCCAAAATCCTTCTTATTAAGCATTCAAACCCCCTTATTTCATGTGGCCCAGAACTCTGGGGAAAAAACAGAGGTTGTCTATTGTTTATCATCCTAAATTTTGTTTCTTTGGGCACTGGTCCTTTCTAACTAATGAGGCCACTGAAATACTTTATAGTGCCGGCTTAGAAGACAATATTATGCTCTTACCATTTTTACATGTTTAAGTTGCATTTGGTCAGACAAGTATTGATTACCTAGATATTTAATTATAATAGGATATAAATCCTATTTCTGAATTATTAATCCCAAACATTGTTAAAACATAATTATTTTAAATGCCCACAAGTATAGAAGTTTCTTTAAGCTATCAAATAACAATATTTAAAAATAATCATCATCCACTCCCGGTCCTCTTACACCATCCCACATCTTATTCCAAAACTCATGTACGGCCGGGTGACTGCCTTAACCCAATTGGAGATGACCTGACTCTTGTTTACAGGGTTTCTGCGGACTGGTTAGTATTCAGTGTGTTGAAGAGATGAGATTTTCCCTCACCATGATAAGAACTTCTCACAGAGTTGCTCGATGTAACGTGGTAGTGAGTTTCCTAGCTTGGAAACACCTTTCAAGGGTGTGGCCTATCTGTGCCAAAGAGGCATTCCTGTATTCATGTTCCAGTCCTGAGAGTATGATGGAATGATCCTTTAAAGGTGTAACTCCTTTTCATGCGCTTGGTTCCTGCCATCCAAGAGATTACAAAGTGTGAAACCGCTTTGTAACTGTAGACAAGAACAGAAAGTTACCATTACTCTTCTGATTCTGGCCACCAAGCCGAAGAAAGTCTATGAAAGCGGTCACCGGGAAAACCTTCCTCAGATGATGTAACATTTTTCAATCTGGAAAGGAAATTAAAATAAAGACTTCGACTTTTTTTTTTTTTTATCTTGAGTACTACTTTTCTTTTTTATAGGAAGTACCAAAGATGACAGGAAAATCACTTTGCAAAGCTACTCATTTTTTGATAATTGGGTGTGTTAAATTACGTTTAACTTTAAAAAGCTTCTCTTTGCATTATTTTAATTTCTTCTTCACGGTTCTTTCTTCGTGATAGTGCACTGTAAACTAACTGGATGTGTAAATAGGACGTTAATCTGCTCTTGTACCAAACACAAGTTTGGTCAAGCAAAGAAGCCAACTGTTCAGATTATGTTCATAATATCGGCCAACTGGCAAGCTATGTAACCCAATCAGCTGTTTGTACGCTCACTTCCATTCGTATTCCTTTCCTTTCTGCCCCTCCATTAAATCCTCTCCAACACATTTGTGACAGCATGAGTGCTTGATTGAACCTCTTTTTCTGTCTTCTGGGGGTGGTTGCCTGATCCTCCAATCATTCTTTGCCCAATTAAAACTCTGTTAAATTTGCTTTTGTTTTAAAGTTCTTAAAAAAAATTTAATGGGTACATAATAGGTGTTATATACTTATTAGCTTACTGAAGCTATTTGATGCGCCTACAATATGTAAGAATCACATCAAGGTAATAGATATCCCTCCCATAACATCACCTCAAGCATTCCTTTGCTTCTGTTTGGGTTCCAGACATTCCCAATTCTACTCATGGTTATTTTGACAATGGCACACTAAATTTGTGGCTGTAAATTACCTGTTGTGCTATCAGATACTAGATCTGTATACCTATCTATATTTTGTACTCATTAACCATTTTTATTCTCCCCCACCACCATACCCCCTCTGAGCCTCTGGTAATCCTCAGACTGGGAGAGAAAACTATTTGCAAATTATCATCCTCTGACAAGGGAGTTAATAACCCAGAATAGATAAAGAACTCTAACAAACTCAATAGCGAAAAAAATGTATACGCCATGTAAAAATGGTGCACGCAAAGATTGAAATAGACATTTCAAGAGAAGACGTCAAAAATGGCAAACCGGGTATATAAAAATGGTGCTCAACATCACCGATCATAGAGATAATGCACATCAAAACTACAAGGAAAATATCATTTCACTTAGGATCATGGCATTTGTCAAACGACAGGCAATAAGGCATATTGGCACGAAATGTTCTAACGTTTTTCTTACCGGTGGAAGTGATGAGGCATATCCTTCCTACCTCTTCCTGAATTTTTCCTTCAGTATTTCTTTTAGGTCCTATTCTGTCTCTTGCTCGTTCTCTCTTTTCCTAGGTTCTGCCTCCCCTACGGTCTATTTTGCTCGGGTCTGAAGGGATAGGTGCCAACGTGAAAGACACGCTCTCCACTCCTAGTAAGCCATGGTTCTTCCTCCTTACCCCCACCACCCCAAGCAGACCACAATGTCTGGCACCATAGCTGTCCTAAATACAATTTGTCGCTGGTTTGAAAGTTATTACGTGGTACTTGCACATATGCTGTCTACACTCTAGTAATCAAAACGGTATCAAGTTTAATTTTTATTGAATGAATAGATATTTTATGTTTAAGAAAATCAAAATAATAAAATAAATACAGAGATTACAAATTAGCACATATATAAAATGAATACATATATTAAAATGATACTACTGAGAAAGCTATCCCTAACCCAGTCTCCACCACTCATTACTCTACCCTTATAGAGGATTAAACCACTTCTATTTAGCCAATTTTGGTGCGTACCTGTTATTATCTCTTATAGAAATGCGAAAGAAACGAAAAGATTTGCACTTAGATCACACGTTTTTTTTAGACAAAAGTCTAACTTTTATGCCAATTAGTACTTTTTTCATTTCACCATCTGTCTTCCTTTCCATGTCGCGCCAATAGAGTATGTAGAGAACTTTCCTCTTTTTTTTTTTTTTTGACGGAGTCTCGGCTCTGCGCCAGGCTGTGATGGAGTGCAGTGGCGTTACTCGGCTCACTGCAAGCTCTGCCTTCCCGGGTTCATGCCATTCTCCGGCCTCAAGCCTCCCGCATAGCTGGGACTTATAGCGATCTGCTGGCTAATTTTTTGTGTTGTTTTTAGGGAGCGACGGTGCTTCACCGTGTTAGCCAGGATGGTCTGATTCTCTGACCTGTCGATCCACCCGCCTCGGCCTCCCAAAGGGCTGGGATACAGGGATGAGCACCGGCCGGCAACTTCCTCAATTCTTTCTAAGGGTGCAGAGTATCATGAACATAGTCAGACAACCAGTCCCGTGTGTATGGAGGGATAGAGCTTTTGCTTTTTCAGTGGGTGTTCTATTTACAAACAATTCTGTAATGAAATGACTGCATTACATTATTTCCTAAGTGTGCCGGTGTACCCTAAGATAAATCCCCATTCATAGACTTGCTCGGATAAATTTGCATTTATCGTTTTACATTATATTGTTACCCTCTCACCCTAAAAATTCCCAAAATTTTCTTCCATACGTGTTGCATCCTTTTCATTTTCTCCAGTAATTACAAGGAATTCATGTTCCCACAGATCTCCACCAAATGTTTGTTAATCATTTGGAGTTTGGAATTCTATCGGTGCGAATTGTTTGTAACTTAGGTGCTATTGTTTTGTCTTTCTTAACTGTGGGATTGCAGTGAACAAAAAAAAACTTTTTTTTTTTTTTCAGATGGGAAGCATCAAGATGCTAGATGAGTAGCAGCTAGTGTGTGTACTCTCACAGAGAGAAAACAAAGTGGGCGAGTTAAACATGCCTCTGCAAGTAGATCACCTAAGCAACGATATCAGGACCAATTAAGGGAGTGATCGTGAGACACAGGCGGAGAACAGGGAAAGAGTAAGCTGGGCAGTGTCCCACCCAATAGGGGCATTGGAGACAGGGGAGGAAGCTTCCCCAACATAGAGAACGGGTTGTGAGTGAGTGAGAGCCTGGGGATCCTCACTTTCCACGGGACTGTGAAATCCTGGGAAAACTGGAGAACCCCTCATCCCAATACTTATCCGTGTCCCTGGGAGCCTCAGACGAATAGAGAGTTTGCTGGTGGAGTGTTTGCAGAGGCACAACTCAAGTCACAGGAAAGTCCCCAGGGGCCTTGGGACCTTGAGCAGTATCATGCAGTGCCATAGCCCTGAGAGAAGGCCAAGTTAGGGAAGCCGTTACATGCTTCACTCCCATCATCCGACCAAGGCTTAGCATCGGCTCAGCACAATGGCCTGGCCTCGCCTGAACTCGTGGGCAGGCACATGCTTTGTACCCTGGAAATCAGTTGGACAGTGGGTGAGGGAGACTAACCTACCCCCACTGCCTGTAGGCCAGGCGGGGGTCATGGTGCTCACAGCAGGGGGGATCCCTACTCTCAGAACACGAAGGGGTGAGGACTGAGACAGCTGGGTTATTGGGACTGGCTCAGGCACAGGGTTGTGCCTCCTTGTGCAGGGCCAGCCAGGTAAGGGTATGGTCTAATTTCAGCCGCGCCTCTGCATGAGGGCACCCTGCAGCTCGGCACTAATAATGAAATGCCGGGCCGTGCCGAATACGAGAGGCTCTCCCAAGGCCCGGAATGTATCTTGGTGAGGGGGGTCTATCTCTCCCTACCACCACCGAGCACTACAGAGGACCCTGCACCAAAATACAAACGAGTGTGGACTAGGTAAGTAATGAGCCTATCTGCCAGCTACCACTCTTAAGCATCACCACTGGATTGCACCCCAACGACAACACTCCAAACAACATTTTTTTTCCAATATACAGCCATCTGTGAAACCAACGCAAAAATATAGCCACAAATAAAGATGCTGTACAGAGCTTTGTCCCTTGAACGACTCAGATGAAGCCAACTGACTATACTCAATGCCATATTGTTACTGGAACACCAGCCCCATAGTTAAAGCTTCATTCAAGAACTCTAGGCAAATTCAAAAAAGCTCGGTGTTACCTTACCTCCAGACAAACACACCAAGTCCTCCAGCAAAAGGCTTTAACCAGATTAAATTGACTCAAATGCCATACATAGAATTCCGAATCTGGATGGCAAGGAAGTACATCAAGATCCAGGAGATCATTGAAACCCCAATACAAGGAACTCCAGGAATCGAATAATGATCAACGAGCTTAAAGAGGTGAATGTGTGAAATAGCCATTTAAGAACGAACAAAACTGAACTTCAGCATAGGAAATTGATGGATTAAACTCAAAAATTTCATAATATAGTCAGAGGCATAACAGCCCAGGAACAGACCAAGCTGAGGAAAGATCTCAGAGTTTGAAGACCAGTTCTTGAATTCAACTTGGTTGGACAAAAATAACAGAAAAAAAGAAATTTTAAAAACATGTGAACAAAATAACCTTCCAAGATTTAGAGGTTATGTAAAGAGGACCACGTCTAGGAACCTCACAGTCATTCCTGAGAAAGAGAAGTAAGCAACTTAGAACATACATCTGAGATGCCGAGTCCACTGATAATTTCCCTATCTCACAGAGCGGTGGGCAAAAATTAAGACACCAGAGAAACTCCTGTGAGACACTATACAAGACAAAAAACCATTGCCAAGTCACATAGTTATCAGATTCACCAAGGTCAATGCAAAAGAAAACATTGTTAAGGCAGCTATTGTGAGAAAAGGCATGTCACTACAAAAAAGGAAACCTCATCAGGCCAGCAACCGTTGTTCAGCAGAAACCTTTACAAGCCAGAAGATAAGGGGGGGCCTATCTTTTCAGAATTATTAAAGAAATAAAGTCAACCAGAACTCATTATCCCACCAAACTCGTTCATAAGTGAAGGAGTAAATACACTCCTTCTCAGACAAGCAAGCACCAAGGATGATTCATTACCACCCAGATGATTCTTACAGGAAAATCCGTTAAGTGAATTGCTAAACAGGCAATGAAAGACGAACCGCTACCAATAAAATACCACTTATTACACAATGAAGTTACAAAACAACCAGCTAAACAACACACTGACGGAGCAAAATCTCAATATGTCAATGCTACCCTCATGTAACAGTATTACACTCTACTTTAAAATGCCTATATTAACGTGGTAAGCTGATAAAAATACCACAACGACAAGACACAAATGTCTGCTGTTCTCAGAGAAGATGCATCCACCATGAATGACACTCACAGGCTCAAACAGTGAAAGAATGACGAAAGATCTACCATGCAAAATGAAAACAAAAAAGAGGGGGAGTTGCTACCCTTACATCAGATTAACACAACTTAAACCAACAACAATCAGGAAGGGACAAAGACGGGAATTATAAAATGATAAAAGGTTCAATTGAACAAAAAAAGACTTCATATTTCTAAACCTATGCACCCAACACTGGAGCACTCGCTTCATAAAACAAATATGCGCCTATCAATAAAACGTAGATGGCAACACCCAATATAGTGGGGGCTTTATCACTCCCACTGATAGCATTATACAGATCACTGAGCAGAAAAACTGACGAAGGAATTCTGAATTCATACTGACACTTGACCCCGTTGGACCAATAGACCTCTCTAGGATACTCAAGCCAACATGACAGAACACACATTCTTCTCAAGGCACATGGAACGGATTTCTAGATTGCCCCACATGCTCAGTCATAAAGCAAGTTCACTAAATTACCAAAATTAAAATCCTACAAAGGCCACTCTCAAATCATATGTGAATTAACAATGAATCAATACAAGAGATCTTCTCAAAACTACACAAAGCTGGCACTTAAGCCACATGCTCCGCAATCAAAATAACTGTTGGGGAACAAGAAAACGGCAGAAAGAAACAAACAAAATTATTTGACATTAATGAAAATAGAGACACACCTTCCAAAATCTCTGTGATCTTTTGGGATGTAGCAAACAAGCAGTGTTTAAGAGGGAACGTTTATAGTGCTAAACTGCCTTCATCAAGAGGTTAGAAAGATACTCAACTAAAACAATCTAATATAACACAGCGGAACTAGGGAAAAACAGAGCACCAATCAATTCACAGCTAGCAGAGGAAATACATAACTAAACTCCGATAATGCCTCTCTAAATAAATTGAGATGCACCCGTTCATAAAATGTTAGTGTGATGAAACAAAGAGAGTTGGGTTTATAAGACAACAAGACTAATAGACTGCTAGCTAGAAAAACGAGAAAAGGAAAGGAAAAAAAAACCAGGACAGAAGTTCAACAAAGCACACGACAGAAATGACAAAAAGGACATACGCCCAATCCCCCACCAGGAATGCCTCCAAGCTTTCAGAGACGTTATTGAAACCTTATGTCACACAAAGTAGGAAATCTAGAGGAAGGGATGAGGGCTAATGGCTACAATTCCTGTGAACGTACAAACCCTTCCCAAGATTGAAACAGGTACGGAGCTGAAACCTTCGAACAGACCTTAAACAAGTTCCACACATTGGCATCCGTAATCAAAAAACCTATCCACCCAAATCTAGCCCCCTGGACCAGCTGGATTCACAACCCCCCCTTTTGAATTCTTACCAAGTCATCCTAAAAAGAAGAACTGATCGTAATCTTACTGAAACCACCTCTTCATAATATTTAAGGCAGATGGGCTCCTCCCTAACTATTCACATGCCAGGCTCATCCTGCTATCAAAATCTGCCATGAGACACAATTTGAAATGAAAAAAGATAAACCTCAGGACAATCTCTCTGATTAACATAATACCACGAAAACCTCAACCAAACATATGCGAAACTAAAATCAAGAAGCACATCAAAAAGTTTAATTCCAACACTCATAGTAGGCTCTATTCCTGGGATGCAATGGGCTGGTTCAAACATAAACATCAATATAATGTGTGGATTCACACATATTAACAGAATGCAACAAACAAAAACCATATGATATCTCACTTTGTTCAGAAAAACTTCTTCAATAACATTCAATTATCCTTCATGATTACCAACCCTCGCATGCTCGGATCAACGCGAATACCTAGAATAAATAAGATCCCCCTATGACAAACACCATCCAACATCATACTAAATTTTTGAAATGTGTGGAAAGCCATTCTATTGAGTATGGAACAAGACAAGTCGCCCACTCTCACCACTATTCAAGCTTGCCCTGGAAGCCCTAGTCCGAGCACTAGGCAAGAGAAAGAAACTTATTACACTGGCCACAAAAAAGAACGATAGAAGGCCAAATGGTCTCCCCTCTCTTGCTTGTGATATTGATTTCCGACCAATAGGACACAAAACCCCGTAAAGCACTAGCACCAACGCTCCCTTAGAAACTGATCAAAATACACCTCAGTAAAGTTACAGGATCACAAAATCAATCGTACAAACATCAAGTATGCACTTCTAATACAACCACATACACATTCTTCACGTTTAGGGCTCTCAAATAAAAACCAAATCCAATGCCATTGACACATTCAGCCACAAAACGGAATACATTCTAACCACTTGGAATTCAGTGAAATGTTCTACAGGGGGAGTGAAACTACAACACACTTGCTGAAAGAAACAAGACTGACTGACAACATAAAAACTACCTAACCCTAACCTGCGTACGGATGGAATATCATCACCACCCATTAAATACGGCACCACGTCCAAGCACCTACCACTCCCAATGCACTCCCTTAGCAAAATATCAGTCCTTTTTCACACCAG

**Supplemental data 8. Sequel read covering 1324-repeat allele.** Defined repeat region highlighted in yellow.

Read name = m54120_170622_200608/58720592/27226_52381

Read length = 25,155bp

----------------------

Mapping = Primary @ MAPQ 40

Reference span = chr9:27,571,677-27,588,962 (-) = 17,286bp

Cigar = 7M2I5M1D26M2D4M1D4M1D3M1D5M1I...1D18M1I18M1D21M1D22M2D16M1I17M

Clipping = None

----------------------

Location = chr9:27,573,569

Base = A @ QV 0

----------------------

H0 = 1

ZE = 0.0

ZF = 0.385482

NM = 10390

ZQ = 25155

ZR = 138394717

AS = 14328

-------------------

Alignment start position = chr9:27571677

CCTGGCTCCTCAAGGATCCTCTTGCCTACGCCTACCAAAGCTGGATTAAGGGTAAGTCACACATTCTCAACCTGGTTTAAATACTGTAACATGATGTTTATTTTCTCCCAGCCCAACGAGAAGAAGGATCCTAAACCGTCCACTTGCCACACAAGGAGCTGCCCGGACCCACGTCACGGACAGTGAATACTGTTTACAGGCCAGAAAGTTCACAAACACTTTCGCAAGCGGCACTACACTCAGGGAAGCGAGGAAGGTGAAGTTATCACTATCTGGCCATTTCTCACAGTGCCAAGTTTCTTCAGACATAGAGGTTAGGCGAAAAACTGGTACTCCTGGGAACTATCTAAGGAACGCTTAGAATAGAACCCGGGGGCAGGAATTGGAAAAACAGAAGGCCTCCTTGTTACGTTGTTCTCTGAAACGCAATATTGTCGGAGTGCCAAACATCGCCAAACAAACTTTTTATCCACTTCATAAGGTGCTGTAATTTTTCTGGAGAGGTAAATGCCCTGGCTTATTGAACATATCTGGAATGTGTTAATTCACTCTTCGTTTCTGTTTCGATGTACTATCAGCAGTAGGCCGTTCAACTGATTAATGGTTTTCTCTAAAGTTGCAGCGCCTTGGCAGTGAGGTGTGTGTAACAATCCCGTTGTCAACTGGACACGTATTTCAAGTATTCTGATCCAGGGTTATTAATACCCTATATGATAGTCTTGTTTCTGTGATATTCACATATTATGTTAAAAAGTTTTCCCAAAGTCTGAGACAAGCATATATCTTAACGTATCTTTTTGTCTTTTTGATCCTGTTACCACACAGTAGAACGTAATGCCAGACAGATACGTACCTTGTTTTGAACAACTGGTGCTGCGGCACACTGTTTGAATAGATATTTACAAACTCCACACCAGGCCACTACTCTCCCAGAGCATAACAATCTCGATTGCATCGAAAGGGCCACCCCTCCTGGGAAAGTCGGACCTTCCCTCCTGTTTACTGCCTACATAAGCATGGTGGTGTTCAACGCGGCAGATAGACCCCAAGAGCCACACGGACATGTAATCTGTGCCACTTCTTATGACCACTGATTACCATCAAGTCAAGTGATGCCCAAGTAACACTAGTCACTTACTTTAAGCAAGTCTGTGTCATTCGGAGCTGTGAACAACCAGGTCATGTCCCACAGCACTGGGGAGGCACAGACTTCGCAGCTGCCTCATATGCAAGTATCAACACTCTTCTAGAAGCGGGGCTGACATTGTGCAGGCGTCTCCCACACCCCCTTCATCCCGCCTGATCTCCTCGCCGGCAGGGACCTCTCGGGTTCCTACGCGAACCACGATGGTCCGACAGAAGCCGCGCGCCGCCCCCCTCGGCCTTCCCCCAGGCGAGGCCTCTTCAGTACCCGAGGCTCCCTTTTTCGGAGCCCGCAGCGGGGCTCCCATGCGGGTCCCCGGGAAGGAGACAGCAGGGTCATGAGGGCGGGGGCAAAGCAAGGAAAGACGGGCCAGCCCATCCCTTGTCCATGCGGCGCCGCGCCGCCGCGCCGCCTGGGAAGGGGGGGGCCAGGGGCCGTATGCAGGCAATTCCCGGTCGCTAGAGGCGAAAGCCCGACACCCAGCTTCGGTCAGAGAATCTGGAGGGAAAGTAAAAACTGCGTCGAGTCCTGAGGAGAGCCCCCCGATTATAACAGCGCCTCTCCCGGCAGCAGAACCCCAAACAGCAACCCGCCAGGATGCCGCCTCCTAACTAAACCAACTTCCGCCACCCGCATGCGCCTCCACGCCGCCGCGGACGAGGAAAAACACGCCAACTCACGAGCCCGCCCCGGGCCCGCCACCGGGACCGGCCCCCGGCCCACGGGCCCCGGCCCTGCCACGGACCCGGCCCTCGGACCGGCACAGGCCACCGGCCCCGGCCAGGCCAGGACCGTACGGCCCGGCCCCGGCCCGGCACCGGGCCCCGCCCCGGCCCCGGACCCGGCCCGGGCCACGGCCCCGGCCCCTGCCCCTGCCCGGCCAGGCCCGGCCCGGCCCACGCGCCCACGGGCCAGGCCCCGGCACCGGCCCCGGGCCCCGGCCCGGGCCTCCGGACCGGCAACGGACCACCGGCCTGGCACGCCAGGCCCCGGCAACGGCCACGAGCCCGGCAACGGCCCGGCCCCGGCCCAGGAACGGCCTCAGGCCAGGCAAGGACCGGCACGGCCCCGGACCAGGCCCCGCACCTGCCCCGGCCCGCCCGGACCGGCAGCACCGGGCCACGGCACCGCAAGGCCCCGGCCCGGCCCCGGCCACCGGCCCGGAACCGGACCAGGCCCGGCCCACGGCCCCGGCACCGGCCCCGGCCCGGCCCGGCCCGGCCCAGCCCCGGCCCGGCCCCGGCCCTGGCCCGCGCCCAGCGCACGGCACACGGCCCGGCCAAGGCCCCGGCCACGGCCCAGCCGGCCACGGACAGGGCACGGCCCGTCCCCGGACCAGGACGGCCCCGGCCCGTGCCCAGGCAACTGCCCACGGCCCCGGCAACGGCCACGGCCCCGGCCGGCACGGCCCGGCCAAGGCCCACGGAACGGACCGGCAAAGCGAACGGCCCCTAGCCCCCGGCACAGGCAACGGCCCCGGCCACGGCCAACGGCCCCCGGCCCCGTGCCCCGCGCCCGGCCAGGAACACGCCAAGGCACACGCGACAAGGCAACCTGGACAGGAAAAGGCAAAAGGAAACGGACACAGGCACCAGGACACAGGCCCAGGCGGACCGGCCCCGGCCCCGGAACCGGCCCGGACAGGCACCGCCGAACGCAAAGGCACCACGAGCCACAACGGAACAGGAAAAGCGCACAGGTCTACCAGGCCCCGGAACGGCCAGGCAAAGACCCGGAACAAAGGCCAGAGAACACGAGTCTACCGACCAGGAAAAGCAACGGCCCCGGAAAAGGAAAGGCACCCGGCCCCGGCACCGGCCGGACAAGGACCCGGCAACGGAACCGGCACATGCACAGGAACACCTGGCCCGCGCCAAGGAAAAGGAACCGGCAACGGCCCAGGCCCCGGACAAAGGACAAGGCCCAAGGACCACGGAAAAGGCAAGGACACGGCACCCGGCACCACGGGCCCGGCACAGTCAACGGCACAGGCAAACCGGCACGGCAACACGGGAACCGGAAAAGGCACGGCCACGGACACGAGCCACGGCCCAGGGGCCCCCGGCCACGAGACCCGGCCCCGGCCCCGGCCCCGGCCCAGGCCCCGGCAACGGAACCGGCCGGCCCCGGACCCGGGCCACGGCACCACGGCAACGGCCCCGGCCCCGGACCCCGCGCCCCGGCCACGGCCCCGGCCCGGACACGGGCCCCCGGCCCGGACCCCGGCCCCGGCCCACGGCCCCGGCCCCCGGCCCCCGGCCCCGGTCCCCGGCCCGCCACGGCCCAGGACCGGACCCCGGACACGGCCCGGACCGGCTCCAGGCCACGGCCCGCCCCGGCCCAGGCTCCGGCCCGGCCCGGCTCCAGGGCCCTGTCACGGCCGGCCCCGTGCCCGGCCCCGGCCCCGGCCCCGGCCCCGGCCCCTGGCCCGGCCCGGCCCCGGCCCCGGCCCCGGCCCCGGCCCCCGGCCCCGGCCGGCCCCGGGCCCCGTGCCCCGGGCCCGGCCCGGCCACGGCCCCGGCTACGGCCACGGCCCCGCTGCCCCGTCACCGGCCCCGGCCCCGGCCCCGCCCGGCACCGGCCACAGCGCCACGGCCCCGGCCCCGGCCCCGGCCCCGGCCCCGGGCCCCGGCCCGCACCGGCCCCCGGCCCCCGGAACGCCCGGCCCCGGCAGGAAACGGGCCCCTGCCCCGGCCCCCGGCCCCGGCCCAGGCCCGGGCCCCGGACCCGGCCCCCGCCCCGGCAAAGCAACGGCCCCCGGCTCCAGGCCCCGGCCCCGGCCGGGCCCGGCCCGGCCCGGCCCCGGCCGGACCCGGCCCCGGACCCGGCCCCGGCCCCGGCCCGGCCCCGGCCGGATCCCGGCCCCCGGCCCGGCCCGGCCCCGGACCCCGGGCCCCGGCCCGGCCACTCGGCCCACGGCCCGGCCCCGGCCCCTGCCCGGCCCCTGCCCCTGCACCGGCCCGGCCCCCGGACCCCGGCCACGGCCCGGCACCGGCCCCTGCCCCGGCCCCGGACACGGCCCGGCCCCGGCCCGGCCCCGGCCCCGGGCCCGGCCCCGGCCCGGCCCGGCACCGGCCCACGGCCCCGGGCCCCGGCCCACGCCCCGGCCCCGGCTCCCGACCCGGCCCCGGGCCCCGGCCCCCGGCCCCGGCCCCGGCCCCGGCCCGGCCCCGGCCCCGGCCCCGCGACCCCGTGCCCGGCCCCGGCCCAGGCCCCGGCCCCTGCCCCGGACTCCGGACCAACGGCCCCAGTGCCCCGGCCCCTGGCCCGCGCACCGGCCCCCGGCCCCGGCCCCTCGCCCCCGGCCACTTCCCGGCCCCGGCCAGGCCCGGCCCCGCCCCGGCCCCGGCCGGCCCCGGCCCCTGGCCCCGGCCCGGACCCGGCCCCGCGCCCCGGCCCCGGCCGGCCCCCGGCCCCGGCCACGGCCACCGGCCCCGGCACCGGCTCACCGGCCCCGGCAACGGCCCCGGCCCGGCCCCGGCCACGGCCAGTGCCCCGAGACCCCCGGCCCCGGCCCCAGGCCCCGGCCAAGGCAAAGGCCCCGCCCCCGGCACCGGCCCACGGCCCATGACCAAGGCCAGCGCCCAGGCCCGGCCCAGGCCCCGGCCCCCGTCCCCGGCCAACCGGCCCAGGACAAAGGACAGGAAGGACCAGGCACCGGCCAGGACGGGACCCGGCCCCGGAAAAGGGCCCGGCACCCGGCCCCAGGCCCAGGCCCGGACCCGGACACGGCCCCGGCAGGACCCGGCCCCGCACGGCCCCGGACCCGGCCCCCGGCCCCGGCCCGGCAATACGGCCACAGCGCAACAGGCCAGGGACCAGGCCCCGGCTCACGGACCGGCCCCGGCCCGGCCTACGACAGGCCGGCCTACGGCCACGGCACCGGCCCCGGACCCGGCCCGGCCCCCAGGCCCAGGCCCACGGCCCCCGGCCCCGGCCACGGACACGCACAAAGGCAAATGCGAAACCGCCACGGACAGGCACCGGCCCCCGGCCACGGACACGGACGACACAGTGCACCGGCAAGGCCCCGGTGGGGGGGGCACCGGCCACGGCCCGGCCCCGGCCCAGGCCCAGGCCACACGGAACAGGAACCAGGACAGGGCCAAGGCCCAGCACGCCGCCCAGGCCACGGACACTGCCACGACACGGCAACCGGCCCCGGGCCCCTGCCCGGACCCGGCCCGGGCCACGGCACCGGCCCCGGCCAAGGCCCGGCCCCGTGTCCACGGCCCCGGCCCGGCCCGCGCCCCCCCGGCACTGACCCGTCCCCGGCCACGGGCCAAGACAAGGCACAGGCAACGGACACGGCCCCTGGCTCCCCGGCCCCGGCCCGGCGGCCACGGCCCGGCACAGGACCGGGCCCCAGAAAAGCGCCACGCCAGGCCTCACGGACCCCGGCCCGGCACAGGCACCGGCCCCGGCAACCGGCCCCGGAACCCCGGGACCCGGCAAGGCCCCGGCACCAGGACCCGGCCAAGGACACCGCAGGCCCGGCCCCCCGGCCCCGGACACGGCCCCCGGCCCCGGCCCGGGCCCCCTGCCACAGGAACAGGGCACCCAGGACCCAGGCACGGAAGGCCCAAGGACCACGGCCCGGCCAAGGACCGGCAACGGCCCACGGACCCGGGCCCCCAGGGCAACGGCCGGACCCCGGCCCCGGCCCCCGTGCCCCGGCCCCGGCCCGGCCCCGGACCCGGCCCCGCCCAGGACAAGGCACGGCACCCGGCACCGGAACGGCCCCCGGCCACACGGCCCCGGCCCCGGCCCGCGCCCGGCCACGGCCGGCCCCGGAACCGGCCCCGGCCCCGGGCCCAGGCCCCTGCCACAGGCAACACGCGCCCGGCCCGGCCCAGGCCCAGGCCCCGGCCCGGCCCCGGGCCCGGCCCCTGGCCCATGCCGGACCCGGACAGGCCACGGCAACACGGCACCGGCAAACGGCACGGCCACGCGCCAACCGGGAAACGGCACCCGGCCCTGCACCGGGCACGGCCCCGGCCACGGCCCCGGAAACGGACCCAGCGGGGGACACCGGCCCCTCGCACCGGCCCGCCCCGGCCCCGGCCCCGGCACCGCGCACCCGGCGCCGGCCCCGGACGGCCCCGGCAGGCCCCGGCCCCGCGCCGGCCCCGGCCCCCCCCACCCCGCCCGCCACGCGCCTCCCCCCGCCCCCCCCTGTCCCGGCCCCCCCCACCCACCTGGCCCCGGCCCCGGACCGGCCCGGCCCCGGCCGGCCCCTGCCCGGCCCAGGCAACGGCCACGGCCCCGGCCCGGCAACCGGACCGGACCCGGCACACGGCCACGGCCCCGGCCCCGGACCCGCCCCAGGCCGGCCCCGGCCCCAGGCACAAGGCCCGGGCGGGGGGCAACCGGACACGGCACCAGGCCCCACCACGGACCGCCACGGCCCCCAGGCCACGGCCCCCAGGCCCCTGCCCCGGCCCAGGACCCCGGCCCAGGACACGGCCACACCGTGCACGGCCTTCCCGCGCCAAGGCCCACCGGACCGGCCCCGGTGCCCGGCCCCGGCAACGGCACCGGCCCGGCAGGCGGCCAGGAAAGGACAAGGCCCCTCGGAACCGGCCCCGGCACCGGACCGGCAACGGACACGGCACCGGCCCGGCCACGGCCACCTGCCGGCCACGGACCACGGCACCGGCACCGGACCAGGGCCCCAGGCCCAGGAAACGGACCACACGACCAGGAACAGACGCCACGGCCACCGGACCCGGCCAAGGGACAGGCCCAGGCACCGACAAGCGCAAGGACCAGGCCCCGGACCAGGCCACGGCATCAGCGCAAAGTCACGGACAAGGCCACGGCCCCTGCCACGGCCAAAGGACACCGGCCCACGGCCACGCCACGGCCCCGGCCCTGGCCCCGGCCCCGGCGCCCCGCCCGGCCCCGGACACGGCCCAACGCGCACACAGCGCCGGGACACAGGCCCCGGACCCAGGCCCCCCGGCCGGGCCCCGGCACCGGCCAAGGACCGGGCAGGCCCAGCAAGGAACAGCAACATGACCCAGGACCCGGACCCGCGACCCAGGACCAGGACCAGGCCACGGGCAACGGCCCCGGGCACCATGCCACGGACCACGGCCCCGGAATGGCCACCGGCAAAGGCCACCGGACCCGGCCGGACAGGCCCGGAAAGGCCAAGCCAGGCCCACGGACACAGGCAACCGGCACAGGACACGGCCCAGGCCCAGGCCACGGCCCCGCAACGGACCGACCTGGCCCCGGACACGGCCCGGCAACGGCCCAGGCCACGGCACCGGCAACCACACCCACCCCCCCCCACCCCCTCCACAGGCCCCGGCCCCCCGGCCCCGGCCCCGGGCACGGCCCAGGCAAAGGCAACGGCCCCGGCCCCTGGCCCCGGACACGGCCCAGGCCAACGGCAAGGCCCAGCGCAAAGGACCCGCGCTACGGACCAGTCAATGCCAAAGGCCCACACGGACCCCGGCCCACCGGCCCCTGACCAGGAAAGGCCCGGACAGGACACAGGGACACACGGAAAAGGCAAGGAACGGATACCAGGCCCAGGACCCGCCGCGCAAAGGCACCGGCACCGGCCCCGGCAAAGCAAGGAAACGGCACCCGGCCCCAGGGCCCCGGCCCGGCACAGGACCCGCCCGGCCAACGGACAAGGAACAGGCCAAGGGAACCAGGACCCCGACAGGGCCACGGCCAAAGAAGGCACGGCAAACGGACGGTAAAGGCAAAGGAAACGGCCAAGGCCCCGGAACAGGAACCGGCACCGGCCACGGACAGGACCGGACTAAGGAAAATGCCCGGCCACGGCCCCGGACCCCAGGCAAGGACAAGGCCCGGCCTGGCAAGGCCCCAGGACCCGGACAAGGAACAGGCAACGGACACAGGCCCCGGCACGGAAGGAACCCCGTAACCTGCAACGGCCCCGGCACGCCCCGGCACGGCCAAGGCACCGGACGGAACCGGCCAAGGACCAGGAACAATGGAAAGGCACGGGCACCGGCACATGGCCACGCCACAGGCAAAGGCCCCGGACACCGGCACACGGCCCCAGGCACGGCAAGCACGGACCAGGACCAGGCCACCGGCCAGGCCCCCGGACAGGACTCAGCGAACAGCACAGGGACAGGCAACGGACACGGAAAGCGCCACAGGCACCGGACCGGCACGGCCCCGGCCCCAGGCCACGGCCCCGGCCCGGCACCCGGACCCGGCCAAGGCACCGGCCCCGGCACAGGCACCGGACAAAGGGCAACGGACCGGACAGGAACACGGCCACCGGCCCGGCCCAGGCCGGCCCAGGTACAAGGACCAGGCCAAGGCAAGGAAACCGGACCCGCACCGGCACCCGGAACCGGCAACGGCAACGGACCCCGCCACGGACCCCGGACCAGGCCCCGGGCCCCCGGCCCAGGGACACGGACACGGGACCAGGACACGGCACCGGCCACGTGCCCCGGCACCGGACCCGGGCCAGGCCCCGGCCAGGCACAAGGCCCAGGCCCCGGCCCCGGCCCAGGGCCCAGGCACAGGCACCCGGCCACTCCCGGCCCGGCCACGGCACCCACACACTGGCACCCGGCCCCGGCCCCGGACCCCCGGCCCCGGCCCAGGACCCGGACCCGGCCAAGGACACCAGGACCGGACCGGCCCCGGCACGGCCCCGGGCCCGGCCCCGGCCCCCCGGCCCGGCCAACGGCACCAGGCGGACAAGGACCCGGACGGCCCCCGGCCAGCAACGGACAAGGAACTCGGCCCCGGACCGCGCCCCGGCCCCGGGCCGTCCACGGCCCAAGGCCCCGGCCCGGCCACGGACACGGCACCGGCCCGGCTAAAGGCCCACGGCCAGGACACGGCCCACACGGCACCGCGCAAAAGGCCCCGGACCCGGCCCCGGCACGGACCCGGCACACTGCCCGGCCCCGCCCGGCCCCGGCCCAGGCCCGGCCCCGGCCCCGGCACGGCCACGGCCACATGCCCCGGACCAGGCCCCGGACAAGGGACAAGGCCCGGACCAGTGGCCCCGGAACCCGGCCCCGGACCCGGGCCCCCGGCACCCGGCACGGCCCACGGCCCCGGCCCGGGCCCACGGCAACCGGCCCCGAGAAAAGGCTATCACCGCGCCCACCCCAGGACACGGCACCCGCCACCGACCACGGACCGGACACACAAGGCAACAGCGCCCGGCCCCACGGCCCCCTGGCACCGGCCCAGGCCCCCGGCCCGGCCCCGGCCCGGCCCCGGACCCGCGCACACGCTGCCCGGCCAGGACCCGGCAACGGACAGGCCCACGCGACCAGGAAGGACCCGGCCACGGACCGGCCCGGAACCAGGCCCCGAGAAACGGGAACCGGAATCGGCCCCGGACACAGGACCAGCGCACCACGGACCAGGCACCGGCCACGGCCCCGCGCAAAGGACACGGCAGTGCCCCAGGCCCCCGTGACCCGGGCACCGGGCAAGGACACGGACAGGACAAGGCCCAGGACCGGCACCGGCCAGGAACCCGGCGCGCCAGGCCCCCGGCACCGGCCCGGCCCGGCCCCGGCACCGGGCACGGGCCCAGGACGGCCAGGCAACGGCCCTGGCACCCCGGCCCCGGCCCCTGCCAAGGGACCCGGCCCCGGCCCCGGACCCGGACCAGGCACAGGACAAGGACACGGGACACGGCACCTAGCGCGCGACTACTCGGTCTACAGAGCCTGGCTATAACGGCTGCGGTTGTTTACCCTCCTTGTTTTCTTCCTGGTTAATCTTATCACGGTCTTTCTTGTTCACCCTCCAGCGAGTACTGTGAGGCAAGTAGTGGCGGCGAGAGGTGGGAAAAACAACAACCACACACTTCCTAAACCCACACCCTGCTCTTGCTCGACCCCGCCCCCAAAGCAGACAGCAACGGCAGCAGGGAGGTGACACACCAAGCGTTTCATCTTTTACGTGGGGAACTTGTCGTGTTTGACGCCCTATCTTTCTAGCGGGGACACTTAGGTTAGTCTGTCTGTTTCTTGTGCAGATGCCCGTTTCTCACGAGGCTAGCGAACTGGTGGCTGTGGCGGCAAACTTGTCTGATTCTTTTATTTAAGACCCGGCTTCGGAGGATGCGTTGGAGCAATAGCGTGTCGCGAACCTTAATAGGGGATGCTCTGGATCCGGAAGAAATCGTGAAGACGTTTCGTGGTTTTGCATGGTTTGTTTGTGCTGTCTATCAGTGGGCGCAACACATAACTTGGTGATGAAATTTAGTTTTTACGTAAGAATGTTAAGTGCATTCAAAACTCCACTGCAAACCTGGTAGGGGAAGCCCGGCACTGCGGGCTGGAATTCCCAACGGTCCCTGCAAAGGTCATCGCAATTTGCTTTACATGTACTAATTCCTCTCAAGCTGATTTTCACACTGGGGAATTCATTTTTGCTAGTTGCAATCTGTGGATGTAGTTGTTTTTTTTAACTTTGGAAAAACGTACATTATGTTTGATGTCTGTCAAAAAAACTTACAAAGTTTTTGAAAACTTGTGTTTTTGGTTGCAGGTTGTATAGATTCCCCTTACTACAGCTGAGTCCGTTTCACCATCAGATGTGTAAAAAAACCAAAGGTTTTTAAACTTTGTGCCTTTTGATCTGCAAGTGTGAGTGCACTTATTACAGTGAGTAGCATTGAATCTTTTTCATCACAAAAATCACACAGAACGTTTTAATCATTCACGAGGAAGAAAGTGAGGTAATAAATACACCCATGGCTCTCAACTTATACACTTCTGCGAACAACAGACCCTTTTCCTACTGTTCTTATGCTAGTGAACGTTGATCATCAAATTGGCGTCATTCTTTCTACCCCAACTAAAAGTGGGGGTAGGGGGTAGAAACGACTTCGGCAAATTGACACTGCTCCCACAGGTATATCCCAAGTCCATCTGCTGCCACTGCCCGTTGTGCCTGCGACAGTTTATATAAAATCGTGCTAAAATGACCTGCTGCGCTCTAATTTTATTCTACCACATCACTCACCAGTTGAAACTCACCAGCTCTCCAGATCCTTATAGTGCCAAGAATTTTCCAAAGAGCACTATGTAACATTTCTTTTTTAGCCAAACTCCCTTTTCTTTGTTGTGGGGATATACCGAAGACCATTATCTACTGTATGCCCTTAATGGCCAATTTTTCTTACCAAATAAATCAATTAATTTTAGAATGCATCTCTGTATTTCTATTTTTCGACTGGACGCTTAAACAAGTAGCTCGCATTTCCCAAGTTTCTACACTGCGTTGTACTTTCTACTTAACGTGGAATTAAAAACCACTGAATTTATCGATAACAGAGCTAGACCCTTGGTTTTGGGGCTTGGGGGGAAACGAAAATTGTAGGGTAGGGTACAAGTTGCAGTTAAGTCCCAAACATCTAGCGATTTCCTGCTACAACATGAGGGACTAGCGCTTAATATTGTGTTAGTCCATTTTACACGCTATAAATACAATAATGAATACTTGGTAATTATATAAGAAACGTTTACATGGCTACCGTTCTGCAGGGCTGTCCAAGAAGCAATGGCTGGATCCAGTTCTGGGAAGGCCACAGGGACATTAAACATCATGATGGAAGGAATAGGGAGACCCCAGATTCACATGGCAGGAACTGGGAAGAGCCGAAATGGGAGCGTGCCTACCATCCGTTAAACAACTTAGATCTTGTCCAGAACCACTTATAGTACCAAGGAGGGGATTGTACCAACCCATTAGAAGCAACCATCTCATCAAATAACTCCCACAGGACCACACTCCAAAATGGGGATACAGCTTGAACCTGAGATGGGTGGGGACAGAGACTCCAAACCATGTATCCAACTCGGCCCCTCCTCACATCTAACTGTCTTCTCAATTGCACAATACTGTCGTGCCTTACCAAACAGTTCCCAAAGTCTTAACTCCGATCCAGCATTCATTCAAAAGTCCCAACGTCCCAAGTCTCACCTGAGACGAGCTGTCCCTTCTACCTATCAACCTGTAATAATCAAAACAGGTAATGCTTCAAAGATACAATGGGGGTATAGGCATGGGCAGATACTGCCATTCCAAAAGGGAGAAATCTGCCAAAGAAAGAGGCTATAGTGCCCACTTGCAAGTCTGAAAGCATCCCGCAGTCATTAAATGTTAAAGCTCTGAATATCAATCTCCTTTGACTCAAAACCCCCCAGGGGAACACTGATGCAATGAGTGGGCTACCACAAACATTGGGCAGCACCAACCTGTGGCTTTTCCCGTTCATCTCCCAACAGTGCTCTCATGGGCTAGCATTGAGTTGCTTGCAGCTCTTTCCAGCTGATGTTTGCAAGTGTTGTGGACTACCATTCTTGGGGTTATGGAGGACGGTGTCTCTTGTCATAGCTCTGCTAGGCATTGCCCCAGGGTGACTCTCTGTGGGGGCTGCAACCCACATTCTTCTCCTTGCCTTCCCTAGTAGATGCTTCTCCATGAGGATTCCACCCCAGTAACAGGCTTCTGTCTGGCATCCAGGCTTTTTCATACATCCTCATAAAATCTAGGCCGAGCCTTATTACGCCTCACTCTTGCATATGTGCTGCCCGCAGGCTTCACAGCTTATGGAAGCCACAAGGCTTATGCCTGCACCTGTGAAGCAGCGCCCTGAACTGTATTGTTACTGGATGAAAGTTCCTCTGCGTTACAGCCTGCAAATCAATGTGGGTCTGCAGCAACCCTCAATGCTCTCAGATAGAAAGAACTTTGACCACGAGGGCATAAGGGAGAAAACGAGGACTGCGACAAGTTTCAGAGCAGGAGTAAACGTTTTATTAAAAAAGCTTTAGAACAGGAAATGAAAGGAAAAGTACATTGGGAGAGGCCCAGTGGTCCCTTCGGCGGTCAATGCTGTTTGACCTTGCACTAGGATCTTATACACGGCCTACTTCTGACATCTTGTGACCACTCTACTGCTTCCTATCCCTAAGGCTGAGCTTGCGCCTGACATGGTGCCATGAATCTGCATTGGAGGTGAGCTGTGTGCCGTGTGTTTACTGGAGTTGCTATTACATGTTAACATGAGCGTTCTCTTCCATTTCCGGGCGAATGCCCCCAAAGGTCATACTTACCATTTTCGACTCTTAATGTGCATGTTAAGCCAACTATCTCAGGTTCACTTCAGATCTTTATTGGAAGCGCCCCGTACCAATTTCAGGTGTTTCTATCTATTGAGAAGTGCCTCTCCCTGGTGCTCGGTGCAACCAATTACGATTTTAGAGAGCCAGGATGACAACTGCTGGAACATATTGATGGTGCCTGCAACTTACTGGTGGGTGGTGGGACGTCTCTTCTTCCCCACTCATGCTGATTAGCTACTACTGTTAACAGTACCTGGGCCCTCTTGCGCCGTGCGTATTCAGGGCGAGGCGGTCCCAAGGCTGTACGAGGGAGCAGGGGGCCTCGGAATGGCACAGGAAATGATTCAGTTCCTCATAGGCCTCGGGGCGTTTTGAGAGAGGAACCACCATGAAAAGCCTAAAATGCCTATTAGGGACACACTCTTACCACTGTTGGTGGGACTGTAATAGTCAACATTGTGGAATCAGTGTGGCGCCTTCCTCAGGGACTAGAATAAGAATCCATTTGACCCCGCCTCCATCCATCAATGGGTATATCCAAAAGGACTATAAATCCTGCTGCATAACGACATGCCACACTATGTTTATTGTGGCACTGTTCACAAATAGCAAATACCTTGGACCAACCCAAAAGTCCAACAGTGATAGCCTGGATTAAGAAAAGTTGGCACATCTACACACTGGAATACTATGCAGCCCTAAAAAATGATTAGCTATGTCCTTTGTCAGGGACATTGATGAATTGGAAATCGTACTTCTCAGTAAAATATTGCAAGGTCAAAAAAAACCAAACACCACCTGTTCCTAATCATAGGGGGAATTGAACAATGAGAACACATGGACCAGGAAGGGAAACATTCACACTTGAGGACTGTGTGGGGTGGGGGTAGGGGGAGGGCAGAATTAGGAGATATACCTAATGCTAATGACGCGTTAATGGTGCCGCCACACCACATGGCACAGTGTATACATATGCAACTAACTGCACATTGTGCACCTGTACACTAAAACTTAAAGTATAATAATCATAAAATAAAAATAAAATTAAAATAAAAATGCCTATTAGTACTATGGTCTTTCTCCCCATGTTTTGGTATCAGCCCTTGCCTTCTTTTTAGTTATGGCCCCAAATTATGCAGCTTGCCTTGACTTCCTCTCTGAGAACGGCTTTCTTTACTAACTAACATCGGACCAGGCTGCAAAATTAACAAACTTTATGCTCTGCTTCTCCTTTTGTGTTCAAGTCCATTACTCCAGGTATTTCTGTGCTCATGCATATGGCATAGGCTATTGAAAGCAGATCGGTTACTTCTTGAACCACTTGCTGCTTAGAAAATTTCTTCTGCCAGATACCCTAACTATATTCTTAAGTCTAAGATTTCACAGATCCCTGAACCGAGGAACAATGCACGCTAAGCTCTTTGCTAAAGCATATCAAACCTGAACTTTTACCATTCCAATAAAATTTCTCATTTCAATTGAGACCTCCTCAGCCGGAACTTTCACTGTCTATATCACTATCAGTATTTGGTTATCCAACCACTCAACAAGTTTCCTAGCGGTTCCAAACTTTCTCATCTCTTTAGTCTTTTGAGCCCTCTAAAGTTTCAACTTCTGCCTCGTTAGCAGGTTCCAAAGTCACTTCTACCATTTTCATGTATCTTTCTAGCATGCCTTCTTCTCCAAGCTAACAATTTTCTGTATTCGTCCATTTTGCATTGCTATAAAGAAATACCTGAGACTGGGTAATTTACAAAGAAACGAGTGTTTAATTGACTATGTTCTGAAGGTGTATAGGACGCATGGCAGCTCAGCTTCTGGGGATGCCTCGAGAACTTACAATCATGGGGAAGGCAAAGAGGGAGCTGGCACTTACATAGCGGGAGCAGCGAGGAACGATGAGACGGGAAAGGGGGGGTGTTGTTCTACACTTTTAAACACCCAGATTCTCGTCGAGAAAAACTTACTCTACTATCTCAGTACCACGAAAGGATGGTGTGACCATTAGCAAGCCCAACCACTGTCGCATCCAATCCCATCCACCCAGGCTCCTCTCAAAAGTGGGGATTGCAAACTGAAACATGAGATTTGGAGGGCTATGGATCAAACCATATCAACATATTGATACTGGAAATTTGGTAGAGAATATATTTTAGATAATCTATCACATCATAACAGGTAGCTATGAATGATGGATATGTTATTTGCTTGGCCTGTAGAATCACTTACACGGGTATATTCAAAACCATTATGTTGCCCATTTTACATAATACATAAAAAAAATTACACTTAATCCTCCTGACATCTCTAGGATGTAGATATTGTCATCCCCTAGGATTGAATGTTATACCGAGGCAGATAACTGGGACCTTCTGAGAGCTTGAATTATTTACCAAGGCTACATACTTTGATAACTTAGGCCAGTTTTCATGCAACTCTAGAGATCACTGATCCAGAGGACCAAACACCTGGTACCAAGTGATGATAACTCATCCCTCAGAAACTCATCTGGTGCGTCACTACTAGCCATCAACAGTTCTAGAGTAAGACTGGAACTTCGGATTTAGGCCTGGGAGGGGCTACAACTCCAAAAGAGGTTTTTCCTACTTCATAAAATTGGCCTACAGACACAATAATTTTCCTTTCCGAATAGGTTTTTCTGTTTTTTTTGTGATATCAACCCGGAACACTATGTTTCTAAGAGAGAAATAATCAAACCAAAGGCCTAATTCTACATGTATGAGAAAAAAATATATGGTGACAAGGCTTGTAGCATGCTGCTTTCTAGCCGTGACTTTGTATCTTCCCCCACCTAATCCAACAAGGTATTTAGTCATTGTGTCAGCAGTATCGATGTTGTATGTCTTTCTTAGATTGGAGCAAAAAAATCAGGAGCAGAACACAATAATTATCAAAGTCCAGTGGTCCAAATTACTTCTTGCCTGAAGTGTAAAAGAGGAGACAAAATTCTAGGCATATATGCAGTATTCTGGGACAAGAGAAAATGATACTTACCGCATCTAGTAATGTGTTCATTATTAGCAAAGCATTGAAAGGATGTTGGAGGGTTTAACCTTATCTTTCATACTTGGTAACTGGTTTATTAACCCCCTCAAGTAACTGTCGCATCTTAAATGAACCTAGAGTTTTTCTACAGAATGTCTTCAATGTGAAAGCGTCTCATTTTCAACCGAATGAACCTAAAAGACCTTATATAGGAATTTGAACTCTTCAAAAATAATGCAGTCAGACAAAAAGACTCTTAATACTCGGGAGCAGAAAAGCATATGGTTAAGAGAGTGGGCTCTGGAATCAGAGCTGTGTGAGTCTTGTCTTGCTTGATTAGTTTACATTTGGGCAGTACGCTTCTCTGTGCTCAGTTTCCCCGCTACAAAATGGCATGCCCGACTAATTCTTAGGATCATTGTGAGGGTCATGTGTGATCAAGCAAGCTAAGGATTTAGAACAGTCCATTCGAGAAGTGGTTCTAATTCAATTTACTACCTATGCAGAGATAAATTAAAAGCAATAACAAATGACTATTAGACTTTAACTATGTTACCTATTATGATTCAATAACTTCTATATCCGAATCCAGCATAACCTTCTGCTGTGTCAAAACTGGCAGTTGTTTCCGGAAGGTTTCTCGCTATTAGTGTATTTCAAACATGCAATATCTGTGTGTTCAGAGCTCGAGCTGCGAGGCTGTTGATTAGCTCCCTTTATCCGATTTACAGCAATTAGATTTACCTGTGACTGGCATTGTGAAACGATCTTATGTATGCATGGAGGAGGACTACCGCTATAAACCATAGTGAATTTAATTTCTTTATCTTTTTTTCTTTTTGAGACCGAGTTCACTCTTGTTGTCCAGGTGGAGTTGCATGTGCCCGATTTCAACCTCCCTCTGGGTTCCAGGCTTCTCTGCTCAAGCCTTCTGAGTCGCTGGGATTATAGCATGCACCCCATGCCCGCCAATTTGTATTTTTCGTAGCGATGGGATTTCTTCCCTGTTGATCAGGCTGGTCTCAAAACTCCCACCCTCAGGTGATCTGCCCTCCTTGGCCTCCGCACGTTCTGAGCTTACAGGTGTCGAGCCCCCGTGCCTGGCTTCATCTTCTTTTCTACTCCTGCTGTCTTTTTCTGTGGTTGCCTCACCTTACAGGACTAGGCGATGCACTCTCATGGTACATTATCTCCCCCCTCCATCCCTCCCCCTCCCCCCCTCCCCTCCCCCCCCCCTTGGTTGGACTGTGACTGAACCCACTCCCTGCGGTTACTTCTAGGTGAAATCTTGATTCTTGCGTATATTAAAGTCCTTTGGTTTGCCCGTGGCTCTAAATGTTTACAAAACTCTAGGGGTTCGTGCCTGGAGGCCAACACTCTACCGTTCGATGATAGACAATGTAAAGCATGCCTTGCAAGGCATATCAGGCCCGGGTGTGGTGGATTCACGCCTGCAATCCCAGCACACTGGACGGCGCGGATGGTGGCTCTTTGAGGTCCGGAGTTCAACACCAGCGACTCCACCCTGGTTACACCATCTCTACTAAAAATACAAAAACAAAAAAAACAATTCGCCCGATGTGGTGGCCACATGCCTTAATCCATCTACTTGGCAGGCTGAGGGCAGGATAGTCCCTTGAACCCGGGAGGTTGAGGTTGCAGTGAGCTGAGATTATGCCCCTGCACTCCCGCTTGGGCAATGGAGTGGGGGACAGAAAAAAGAAAAAAAGAGACTCATCAAGGATCCAACCTACTATTTCCTTTTGGTTTTTCTTGGGACTTATGATTTCTATAAATAATAACGCTAAATGGAACTTTCCAACCAGAGAATCACAATCTTTGACCAGACCTTAACCTCTATTCCTTCTCTGTTGCTTTTGCACTTTCAAGCAAACTAGTTTTGAGTGATCTAAGGCCTGGCAATACCAAGTTCTGGGGAGAGGAGTTCCCTCGTTCATTGGCAGCCTCAGAAAACCAGAAGAAAGAGAACAAGAAGTAGGAAATGACGCCGATCTTCTTACACTTTTAGAGAAGCCACTAGCTTATCGAGGAAGAGAGAATTTCGAACTAGTACCTTGATAGGTTGGTCATAATTTGATGGACTTTAGATGGAAAGAACAGTGCCCACATGATGGTTTTGTTACTGGACCTCCATCTGATGAGTAGTGGGGGACTGTAGAATGATGGCCTCAGCACCTCACCAGCAATGGCCCCTTCGAGATACTCCCCAAATCCCCTCAGAATGGTTCGCTGAGCAACCGGCCTAGCTTTACCCCTTCCTTGCAGCTTCAGCATTATTTTGCATGTATAGTTGTATTTACTTGTAGCTACTTGTATAGTGCTTTGTAAGTAAGTTTCTTTAGTTATTTATGATGATATGGGGAAAAAAATGTTCTCAACTCTGTCACATGCTATTTGAGAACTGACGTAGGCTGACCAGTTATTTGTTAAATCTTATGTCTAGCTCCTATCCGATGAATTAGCAAATGCCAACGTAGCTGATTGAAACTTCCCTCAACTACCCAACCCCCCCGCCCCCCCCCCCCCCCCCCCCCCCCCCCCCCCCCCCCCCCCCCCCCCCCCCCCCCCCCCCCCCCCCCGAATTACTTATCCATTCGAAGTTAGGCTAGTTTTATATATTCATGCATGTTATTTGGAAACCTTATAAATATTCCTTATTGGCTTGTAAATTTAAACACGACCAAACAAAACAAAACTAAGAAAGCAACCAAACACAACAAGAAACCAAGTCCTAAAACAAAAAGTTACTAATCATTTCAATCTCATGTTCTTCACCTGCCTAAGGGTTCCATTTTTATATGTCAGGTCCTTGGCACTGTGACTCTCTAGTTTCACCTCTGGCTCCCTCTGATTCTAAGCTCTCTCTTACGCCCTTTCCTTTGATCTTTACAGACGATGACTGTTTACTGCTCTGTAACCTCTCATATAATTTCTCTCTGCCTGCAATGTCCTCCATCTCCATTTTGTGTGAGCAACCTTATTAACGCAGCTATAGCTGCCTCATCCCTGAAGTCTTCCTGATTTTCCCAGTTTGACTATTCCATTGTGCACCTTTAACCTCCACTAGTAATACAAAAAACAAATCTTTGTATTTACATTTTCATTTTGGGGCACATAATCTGGTAAATATTATTAGAAACTAATAATTTGACATAGTAGAGTTGTTTGCAGTGACCACAATAGGTTAATCGGCATGAGTTTTGGGTAAACCAACATAGTCTGATACTTTATGGTTCCAATCACTTTCGCATCCTGGGTGAGTACTTAAACATTTTAGGCTCAATTTCCTCTCTTGAAATGGAATGCTTGAGGATTAAATGAGGTTACCATCATAAAGTGTTTATCTTGATACCTGCCCCATGTTAATTGCCTCGTAAGTTTAGCTACTATTATCACATGTAGACGCTCAATTTGTGTTTTGTTAATCAAATGTATTCCCAAACCCCTACGTCTGTAGCTGAGAGATGAAGGGAAGCGGGACAGAGATTTACTCGAGGTGAAACCTTTAGTCTTGGGGCCGACTCTGAACCTAACACTTTTCATATGCCAGCAACAGGTAAGAGCTTTTTTTTTTTAACATCAAAATAGGGGTGTTACGTCTTCTCATCAACTATGATGACGCTTCGGGGGAGGGGCATTGTCTGATTCACTTCGTCTGATGTTTTCCTCTAAGCACAGTAATTGCCCTATGTTTTAGGTCTTTAAGAATGTGGATGTAATTGTAATTTAAGAACAGTTCCATTAGAACAAAAGAAAGTTCCCATTAGAAACACGAATAAATAAACTGTATTTAATTCAATGTGTAGATAACTTTCACTACGCTCTAAATCTGGCACACTCAAGTGATGTTAATAAAGAAAGCTTAATTTGAACCATATTGCCTTTTTAGTGACAAAACAAAGGCCACTTCATCTCAGATTTCTAGAAAGAGCGTTATTTCATACATTGCACCAAAGCCATAAGAGATTCAAACACATATTTTTACCACCGCAGTTGCCACCCACGGCATTTCAATCAGTTTAAAATTTCCTAAACAAATATAAAGTTATTGCTCTAAAGAAGTAATGAATTTTTTGTTATTTTTCTGACAAGTTATTACGGAAGGATTAATTTCAGTACCCTGTGAGAACAAAACTGAGTCACAGGTAGTAAAGTCTGCTTACAAAAAACACTAAGACGGGATGATTGTCATGTTATTGTTTTTCATCCAAAATGTAGAGAGATAGGTCACCTTAATCGCTTGCTGGTGAATTAAGAATGTCTCTTTTGTCCTATTTAAAGATGTTTTTTTTTTTTTTCTGCAAACGCTTTCAAATAAATTTCTTAGAAAAAGAGACATGCACAATAGATTTAGTAACAGAACAAACAAAACTCGATGATAAATAGGAGGTGTATATTTCAAATTATAGTATTAACTCCTCAAAATTATTTTCTTACTTGGAAATGATTGTTTGCCTGTCCATATTCCATGTGAGGATCTAAAACGCGAAATAGTGCTCAAACATTGCGGATCACCTCTGACGACTATCAGTTGTTTTTTTACCAAATAGTGCTTTAGAAGACTGAAGATTGTGCCCATGGGAAATGACATTAGGATCTAATTCTCAAGCACCGTTCTCATTAATGAACTATACATTATCATTCAGGCGACAAGACTTTGTTTTTGACCCGGGCAAAAAGAAAAGAAAAGTAAGTGGTCCATTGATAAAGAGCAGACTACCCACACTTTAGTTTGTACAGCAAAGATCAATCTATCTCACAGTTCTCGAGTTTTTAAAAACGCCTCTTTATCACGGAATAATTTTTTTTAAAAGTCAAATTAAAAACAACACGAACAAAAAATTCAGTTCGATAACTTTAAATGCCTAAATAATGTATTGTGGACAAAACACCATAAAAAGCTGCATTAAACTTCATGATTAGTATAGGGTAGCTATTACATAGTACACGATCAGCTCTTGCCCAGTGTTGTTAAAATTACTTATCCCACACACAGCAGCTTCATCTTAAGGGAACTCCCAGAAAAATGCTTGGGTGGAGAAATGCAGATTGGGTTATGACGTAATTTTCGTAATCAAGGACAGATTTTTCTTTATTTAATTAAAATTAACTTCATCGTTGGAGCAAGATTCATTACTTATCTACTAAACGCAGAACAAGCCCCAAAGCCATCCCCTTCCACGGTCTCACTGTCACTTTCGCATTCCAAACACACTAAATTTCCAATGAGTAGGTAATCCTTCCTTACCGAGCAAAAAACCTTGTGGAGTTTGAACTTAGGTGACAGCACTTTTTTCAAAATATTCTTTTTTTTTTACGAGAAAGAGTCCTACTCTCTATCTGTTGCAAGGTAGAGTGCAGTGGTGTGATCTCGATATTGCAGCTTGGCCTCCCGGGTTCAAGTTGATTCTCCCCTCAGTCCTCCCGAGTCGCGGGCTACAGGGTGTACCACCAACCCGGGCAATTTTGTATTCTTTAGTAGAGATGGGGTTTCACCCACGTTGGCCACGCTGGTCCTTGGCAACTCATTGGGCCTACAAGTGATCTGCTCACCTTGCATCCCAAATTGCTAGATTACAGGCTGAGCCACTTGTGTGGGCCAAAATAATTCTTATTAAGCATTCAAACACATTATTTCAATGTGGCAGAATCTCCGTGGGTTGCAAAAAAGTAGCGTTGTCTCTTGTTTATACTAAATTTGTTTTTTTGGCACTTGGTCCTTTATAAATGAGGCCCGAAATATGTATAGTGCCGGCTTCGAAGGAACTTCTTAGCCTATTTACCATTTTACATGTGAAGTTGCATTGCAACAAGATGAATACAATAGTATATTTATTGTATAATCCTGTGATATTAATATACTATTTTGAATGAATCCAAACAGTGATAATAAACCATAATTATTTTAAATGCAAAATGTATTAGAAGTCTGCTTGTCGATCAAATGAACAATAAAAAATCTATCATCAACTAATACTCCGTCTACCACCATCCCACATCCTATTCCAACATAATGTAAGTGCGGGGACTGCTTTAACCCAATTGGATGAATGAATTCTGTTACAGGGTTCGGGATGGTAGTAGTCAGTGTGTTGAAGAGATGCGATTTTCCCTCACCCAGATAAGAACTTTCACCGGCTTGCATCGCATGATAACGTGGTTAGATGAGTTCCTAGCTTGCGAACACCCTTAAGGGTGGCCTACTGTGCCAAAGAGGCATCAGGTATTCCATGTTCCGTCCGAGAGTCATGATGGACTGACTCCTTAAAGGTGCTAAATCCTCTTTTCTGGCTTCTGGTGCTTGCATCCACCGAGCCATTTACAAGTGAAACGCATTTGTAAAATGTAGGACACGAACAGCAAGTTACAATTACTCTTCTGATTCGGGCCAACCCATGCAGCACGCAATGTTCTATGCACACGCGGTCAAAGGGAAAAACCGTCTCAGCTGATTAACATTTAAATTGGAAAGGAATAAATCAAGACTTGCGGACTTTTTTTTTTTTATGTGAGTCACTACCTTTTCTCTTTTTCTCAGGACGTACAACCATGACAGGAAAATCCCACTTTTGCAAAGCTACTCATTTTTTGATAATGGGTTGTTAACATTAAGTTTAAACTAAACGCTTCCTCTTTGCTATTAGTTCTGCTTAACGGTTTCCTTCTTCGTGTATAGTGAACTGTAAATACTCGGATTGTGTAAATTAGACTGTAATTCTGCCTTTTGTCACCAAACACCAAGTTTTGGTCTAGAAAGACATCCAATGTCAGATTATTTCAACTAACGCAAATGGCAAGCTGTAACCAATCCCAGCTGTTTGTGTAACCTCACTTCCATTTTTGTATCCTCTTCCCTTTTCTGGCCTATAAATCCCTCCAACCATGTGACAGCACGGTTGCTTTGAGAAACCTCTTCTGTTCTGGGGGTGCCGATCCTTCAATCATTCTTTGCCAATTAAACCTGTTAAATTTGTATTTGTTAAAGTTCTTAAAAATTTAATGGGCTCCATAATAGGTGTATATACTTAAAGATCAGAGCTAATTTGATGCAGGAATACAATATGTATAATCAACTAACGGTAAATTAGATCTCCATCACACTCCACACCCTCCAAGCATTTATTGCTTCTTTGTGTACAGGCATTCCAATTATACCTCTTAGTTCTTTTGAAATGCACAATAATTTTGTTGGCTGTTAATTACCCTGTTGTGCTATCAGTACTAGATTTATTACTCCTAATCTTATATTTTGTATCTTAACCACTTTTTATTCTCCCCACCACCATACCCCTCTGCGCCTCTGGTAATCTCAGAATGGGAGAAATATTTGGCAAACTTATTCATCTGACAGGGATTAATAACCAGAATATATCACCGAACTCAAACAAATCCAATAGGAAAAAAATGTATTAGCCATTTAAAAATGGGCAAAGATTCTGATAGACATTTTTCAAGAGTACGCACGTACAATTGGAAAAAGGTATATAAAAAAGTGTCCACATCACCGATCATGAGAGAAATGCAAATCAACACTACAATGAACCTATCATTTAACTCGAGTGTGATAAGGCATTTGTTCAAAATACAGGCAATAGGAATATGGCAAGAATGTCTAAAGTTTTCTTCAACAGGTGGAAGTGATCGGCCATTCCTTCTACTCTTCTCGAATTTTTCCTCAGTATTATTTAGCTACTATTATGTCTCTGCTGTTTCTCTTTATCATATGTGTTCGCTCTAGGTCATATTTATGCTAGGTCTTGAAGGAGAGAGGTGCAAAGTGGAAAGAACAAGATTCGACCTATGTAAGCCTGGTTTCCCTCCCCTCTACCCCACCACCCAAAGCAGACCACAATGTCTGGCACCTAGATGATCATCAAATACTAATTTGTACGCTGGTTTGAAATTATATGTGTTACTGAACAAATGACTTCTCCTACACATCAGTAATCAAAAAATGTCTCACGTTAATTTTATGAAGGAATACTATATTATGTTAAGAAAATCAACAATATAACATCAAATACTATTAACAAATTAGTACTATCTTCTAAATGAATACATATATTAAAATGAATACACTGAGAATTTCTATCCCTCACCAGTCTTCATCCACTCAATTACTTTACCTTATGGAAACACTTTTTATTCTAGGCCAATTGTGTACTTTATTATCTCTTAATATAACTGGAAGAAAATATAAATATGCATTATATTCAAACCTTCTTTGCTTAGACAAAAAAGTCTTACACTTATGGCAATTACTGTACTTGTTATTCACCATCTGTCTTCCTTCCCATGTCAGGCATAGAGAAACTCCTCCTTTTTTTTTTTTTTGACGGAGTCTCTCTCGTCGCCCAGGTGGATGGATTGAGTGGCGTTATCTCGGCTCCTGGCAAGCTCTGCCTCCCGGTTTCCTGCCATTCTCCGGCCTCAGCCTCCCGGAATAGCTGGGGGGGTGGTAACTGAACAATAGGCGCCCTGCCTGGCTAATTTTTTCTGTATTGTTTTTAGTAGAGACGCGGGTTTCACAGTGTTAGCCGGCTGGTCTTTGATCTCTGACTCGTGATCCACCGCCTCGTCCTCCCAAAGGTGGGTACAGGCATGAGCCCCGTGCCCGGCCACTTCCTCACTTCTTTCTAAGGGTGCAGAGTATGCCTGAAAGTTAGTCATGAAACCAGTCATGGTGATGGTGGATCTATTGAGTTGCTTAGGGTGTTGCTATACAACAAATTTCTGTAATGAATGACTGCATACATATTTCTAGTGGCAGGTGTACCCTAAGATAAAATCCCCATTCAGAGAATGAGCGGATAAATGCATGTATGTTTTAAATAGAGTGTGCCCCTCCCCCAAAAATCCCCATAAGGTTCTTCCATAAGT

**Supplemental data 9. PacBio Sequel consensus sequence by Long Amplicon Analysis (LAA2).** This consensus sequence was supported by 301 subreads and has an estimated 99.91% base accuracy. Defined repeat region highlighted in yellow. Possible G_3_C_2_ interruptions in red.

>Barcode0_Cluster1_Phase1_NumReads301

AATTCTTACATGTAAAGGCAAAATTGCGATGACTTTGCAGGGGACCGTGGGATTCCCGCCCGCAGTGCCGGAGCTGTCCCCTACCAGGGTTTGCAGTGGAGTTTTGAATGCACTTAACAGTGTCTTACGGTAAAAACAAAATTTCATCCACCAATTATTTGTTGAGCGCCCACTGCCTACCAAGCACAAACAAAACCATTCAAAACCACGAAATCGTCTTCACTTTCTCCGGATCCAGCAGCCTCCCCTATTAAGGTTCGCACACGCTATTGCGCCAACGCTCCTCCAGAGCGGGTCTTAAGATAAAAGAACAGGACAAGTTGCCCCGCCCCATTTCGCTAGCCTCGTGAGAAAACGTCATCGCACATAGAAAACAGACAGACGTAACCTACGGTGTCCCGCTAGGAAAGAGAGGTGCGTCAAACAGCGACAAGTTCCGCCCACGTAAAAGATGACGCTTGGTGTGTCAGCCGTCCCTGCTGCCCGGTTGCTTCTCTTTTGGGGGCGGGGTCTAGCAAGAGCAGGTGTGGGTTTAGGAGGTGTGTGTTTTTGTTTTTCCCACCCTCTCTCCCCACTACTTGCTCTCACAGTACTCGCTGAGGGTGAACAAGAAAAGACCTGATAAAGATTAACCAGAAGAAAACAAGGAGGGAAACAACCGCAGCCTGTAGCAAGCTCTGGAACTCAGGAGTCGCGCGCTAGGGGCCGGGGCCGGGGCCGGGGCCGGGGCCGGGGCCGGGGCCGGGGCCGGGGCCGGGGCCGGGGCCGGGGCCGGGGCCGGGGCCGGGGCCGGGGCCGGGGCCGGGGCCGGGGCCGGGGCCGGGGCCGGGGCCGGGGCCGGGGCCGGGGCCGGGGCCGGGGCCGGGGCCGGGGCCGGGGCCGGGGCCGGGGCCGGGGCCGGGGCCGGGGCCGGGGCCGGGGCCGGGGCCGGGGCCGGGGCCGGGGCCGGGGCCGGGGCCGGGGCCGGGGCCGGGGCCGGGGCCGGGGCCGGGGCCGGGGCCGGGGCCGGGGCCGGGGCCGGGGCCGGGGCCGGGGCCGGGGCCGGGGCCGGGGCCGGGGCCGGGGCCGGGGCCGGGGCCGGGGCCGGGGCCGGGGCCGGGGCCGGGGCCGGGCCGGGGCCGGGGCCGGGGCCGGGGCCGGGGCCGGGGCCGGGGCCGGGGCCGGGGCCGGGGCCGGGGCCGGGGCCGGGGCCGGGGCCGGGGCCGGGGCCGGGGCCGGGGCCGGGGCCGGGGCCGGGGCCGGGGCCGGGGCCGGGGCCGGGGCCGGGGCCGGGCCGGGGCCGGGGCCGGGGCCGGGGCCGGGGCCGGGGCCGGGGCCGGGGCCGGGGCCGGGGCCGGGGCCGGGGCCGGGGCCGGGGCCGGGGCCGGGGCCGGGGCCGGGGCCGGGGCCGGGGCCGGGGCCGGGGCCGGGGCCGGGGCCGGGGCCGGGGCCGGGGCCGGGGCCGGGGCCGGGGCCGGGGCCGGGGCCGGGGCCGGGGCCGGGGCCGGGGCCGGGGCCGGGGCCGGGGCCGGGGCCGGGGCCGGGGCCGGGGCCGGGGCCGGGGCCGGGGCCGGGGCCGGGGCCGGGGCCGGGGCCGGGGCCGGGGCCGGGGCCGGGGCCGGGGCCGGGGCCGGGGCCGGGGCCGGGGCCGGGGCCGGGGCCGGGGCCGGGGCCGGGGCCGGGGCCGGGGCCGGGGCCGGGGCCGGGGCCGGGGCCGGGCCGGGGCCGGGGCCGGGGCCGGGGCCGGGGCCGGGGCCGGGGCCGGGGCCGGGGCCGGGGCCGGGGCCGGGGCCGGGGCCGGGGCCGGGGCCGGGGCCGGGGCCGGGGCCGGGGCCGGGGCCGGGGCCGGGGCCGGGGCCGGGGCCGGGGCCGGGGCCGGGGCCGGGGCCGGGGCCGGGGCCGGGGCCGGGGCCGGGGCCGGGGCCGGGGCCGGGGCCGGGGCCGGGGCCGGGGCCGGGCCGGGGCCGGGGCCGGGGCCGGGGCCGGGGCCGGGGCCGGGGCCGGGGCCGGGGCCGGGGCCGGGGCCGGGGCCGGGGCCGGGGCCGGGGCCGGGGCCGGGGCCGGGGCCGGGGCCGGGGCCGGGGCCGGGGCCGGGGCCGGGGCCGGGGCCGGGGCCGGGGCCGGGGCCGGGGCCGGGGCCGGGGCCGGGGCCGGGGCCGGGGCCGGGGCCGGGGCCGGGGCCGGGGCCGGGGCCGGGGCCGGGGCCGGGGCCGGGGCCGGGGCCGGGGCCGGGGCCGGGGCCGGGGCCGGGCCGGGGCCGGGGCCGGGGCCGGGGCCGGGGCCGGGGCCGGGGCCGGGCCGGGGCCGGGGCCGGGGCCGGGGCCGGGGCCGGGGCCGGGGCCGGGGCCGGGGCCGGGGCCGGGGCCGGGGCCGGGGCCGGGGCCGGGGCCGGGGCCGGGGCCGGGGCCGGGGCCGGGGCCGGGGCCGGGGCCGGGGCCGGGGCCGGGGCCGGGGCCGGGGCCGGGGCCGGGGCCGGGGCCGGGGCCGGGGCCGGGGCCGGGGCCGGGGCCGGGGCCGGGGCCGGGGCCGGGGCCGGGGCCGGGGCCGGGGCCGGGGCCGGGGCCGGGGCCGGGGCCGGGCCGGGGCCGGGCCGGGGCCGGGGCCGGGGCCGGGCCGGGGCCGGGGCCGGGGCCGGGGCCGGGGCCGGGGCCGGGGCCGGGGCCGGGGCCGGGGCCGGGGCCGGGGCCGGGGCCGGGGCCGGGGCCGGGGCCGGGGCCGGGGCCGGGCCGGGGCCGGGGCCGGGGCCGGGCCGGGGCCGGGGCCGGGGCCGGGGCCGGGGCCGGGGCCGGGGCCGGGGCCGGGGCCGGGGCCGGGGCCGGGGCCGGGGCCGGGGCCGGGGCCGGGGCCGGGGCCGGGGCCGGGGCCGGGCCGGGGCCGGGGCCGGGGCCGGGGCCGGGCCGGGGCCGGGCCGGGGCCGGGGCCGGGCCGGGGCCGGGGCCGGGGCCGGGGCCGGGGCCGGGGCCGGGGCCGGGGCCGGGGCCGGGGCCGGGGCCGGGGCCGGGGCCGGGGCCGGGGCCGGGGCCGGGGCCGGGGCCGGGGCCGGGGCCGGGGCCGGGGCCGGGGCCGGGCCGGGGCCGGGGCCGGGGCCGGGCCGGGGCCGGGCCGGGGCCGGGGCCGGGCCGGGGCCGGGGCCGGGGCCGGGGCCGGGGCCGGGGCCGGGGCCGGGGCCGGGGCCGGGGCCGGGGCCGGGGCCGGGGCCGGGGCCGGGGCCGGGGCCGGGGCCGGGGCCGGGGCCGGGGCCGGGGCCGGGGCCGGGGCCGGGGCCGGGGCCGGGGCCGGGGCCGGGGCCGGGGCCGGGGCCGGGGCCGGGGCCGGGGCCGGGGCCGGGGCCGGGCCGGGGCCGGGGCCGGGGCCGGGGCCGGGGCCGGGGCCGGGGCCGGGGCCGGGGCCGGGGCCGGGGCCGGGGCCGGGGCCGGGGCCGGGGCCGGGGCCGGGGCCGGGGCCGGGGCCGGGGCCGGGGCCGGGGCCGGGGCCGGGGCCGGGGCCGGGGCCGGGGCCGGGGCCGGGGCCGGGGCCGGGGCCGGGGCCGGGGCCGGGGCCGGGGCCGGGGCCGGGGCCGGGGCCGGGGCCGGGGCCGGGGCCGGGGCCGGGGCCGGGGCCGGGGCCGGGGCCGGGGCCGGGGCCGGGGCCGGGGCCGGGGCCGGGGCCGGGGCCGGGGCCGGGGCCGGGGCCGGGGCCGGGGCCGGGGCCGGGGCCGGGGCCGGGGCCGGGGCCGGGGCCGGGGCCGGGGCCGGGGCCGGGGCCGGGGCCGGGGCCGGGGCCGGGGCCGGGGCCGGGGCCGGGGCCGGGGCCGGGGCCGGGGCCGGGGCCGGGGCCGGGGCCGGGGCCGGGGCCGGGGCCGGGGCCGGGGCCGGGGCCGGGGCCGGGGCCGGGGCCGGGGCCGGGGCCGGGGCCGGGGCCGGGGCCGGGGCCGGGGCCGGGGCCGGGGCCGGGGCCGGGGCCGGGGCCGGGGCCGGGGCCGGGGCCGGGGCCGGGGCCGGGGCCGGGGCCGGGGCCGGGGCCGGGGCCGGGGCCGGGGCCGGGGCCGGGGCCGGGGCCGGGGCCGGGGCCGGGGCCGGGGCCGGGGCCGGGGCCGGGGCCGGGGCCGGGGCCGGGGCCGGGGCCGGGGCCGGGGCCGGGGCCGGGGCCGGGGCCGGGGCCGGGGCCGGGGCCGGGGCCGGGGCCGGGGCCGGGGCCGGGGCCGGGGCCGGGGCCGGGGCCGGGGCCGGGGCCGGGGCCGGGGCCGGGGCCGGGGCCGGGGCCGGGGCCGGGGCCGGGGCCGGGGCCGGGGCCGGGGCCGGGGCCGGGGCCGGGGCCGGGGCCGGGGCCGGGGCCGGGGCCGGGGCCGGGGCCGGGGCCGGGGCCGGGGCCGGGGCCGGGGCCGGGGCCGGGGCCGGGGCCGGGGCCGGGGCCGGGGCCGGGGCCGGGGCCGGGGCCGGGGCCGGGGCCGGGGCCGGGGCCGGGGCCGGGGCCGGGGCCGGGGCCGGGGCCGGGGCCGGGGCCGGGGCCGGGGCCGGGGCCGGGGCCGGGGCCGGGGCCGGGGCCGGGGCCGGGGCCGGGGCCGGGGCCGGGGCCGGGGCCGGGGCCGGGGCCGGGGCCGGGGCCGGGGCCGGGGCCGGGGCCGGGGCCGGGGCCGGGGCCGGGGCCGGGGCCGGGGCCGGGGCCGGGGCCGGGGCCGGGGCCGGGGCCGGGGCCGGGGCCGGGGCCGGGGCCGGGGCCGGGGCCGGGGCCGGGGCCGGGGCCGGGGCCGGGGCCGGGGCCGGGGCCGGGGCCGGGGCCGGGGCCGGGGCCGGGGCCGGGGCCGGGGCCGGGGCCGGGGCCGGGGCCGGGGCCGGGGCCGGGGCCGGGGCCGGGGCCGGGGCCGGGGCCGGGGCCGGGGCCGGGGCCGGGGCCGGGGCCGGGGCCGGGGCCGGGGCCGGGGCCGGGGCCGGGGCCGGGGCCGGGGCCGGGGCCGGGGCCGGGGCCGGGGCCGGGGCCGGGGCCGGGGCCGGGGCCGGGGCCGGGGCCGGGGCCGGGGCCGGGGCCGGGGCCGGGGCCGGGGCCGGGGCCGGGGCCGGGGCCGGGGCCGGGGCCGGGGCCGGGGCCGGGGCCGGGGCCGGGGCCGGGGCCGGGGCCGGGGCCGGGGCCGGGGCCGGGGCCGGGGCCGGGGCCGGGGCCGGGGCCGGGGCCGGGGCCGGGGCCGGGGCCGGGGCCGGGGCCGGGGCCGGGGCCGGGGCCGGGGCCGGGGCCGGGGCCGGGGCCGGGGCCGGGGCCGGGGCCGGGGCCGGGGCCGGGGCCGGGGCCGGGGCCGGGGCCGGGGCCGGGGCCGGGGCCGGGGCCGGGGCCGGGGCCGGGGCCGGGGCCGGGGCCGGGGCCGGGGCCGGGGCCGGGGCCGGGGCCGGGGCCGGGGCCGGGGCCGGGGCCGGGGCCGGGGCCGGGGCCGGGGCCGGGGCCGGGGCCGGGGCCGGGGCCGGGGCCGGGGCCGGGGCCGGGGCCGGGGCCGGGGCCGGGGCCGGGGCCGGGGCCGGGGCCGGGGCCGGGGCCGGGGCCGGGGCCGGGGCCGGGGCCGGGGCCGGGGCCGGGGCCGGGGCCGGGGCCGGGGCCGGGGCCGGGGCCGGGGCCGGGGCCGGGGCCGGGGCCGGGGCCGGGGCCGGGGCCGGGGCCGGGGCCGGGGCCGGGGCCGGGGCCGGGGCCGGGGCCGGGGCCGGGGCCGGGGCCGGGGCCGGGGCCGGGGCCGGGGCCGGGGCCGGGGCCGGGGCCGGGGCCGGGGCCGGGGCCGGGGCCGGGGCCGGGGCCGGGGCCGGGGCCGGGGCCGGGGCCGGGGCCGGGGCCGGGGCCGGGGCCGGGGCCGGGGCCGGGGCCGGGGCCGGGGCCGGGGCCGGGGCCGGGGCCGGGGCCGGGGCCGGGGCCGGGGCCGGGGCCGGGGCCGGGGCCGGGGCCGGGGCCGGGGCCGGGGCCGGGGCCGGGGCCGGGGCCGGGGCCGGGGCCGGGGCCGGGGCCGGGGCCGGGGCCGGGGCCGGGGCCGGGGCCGGGGCCGGGGCCGGGGCCGGGGCCGGGGCCGGGGCCGGGGCCGGGGCCGGGGCCGGGGCCGGGGCCGGGGCCGGGGCCGGGGCCGGGGCCGGGGCCGGGGCCCGGGGGCGGGCCCGGGGCGGGGCTGCGGTTGCGGTGCCTGCGCCCGCGGCGGCGGAGGCGCAGGCGGTGGCGAGTGGGTGAGTGAGGAGGCGGCATCCTGGCGGGTGGCTGTTTGGGGTTCGGCTGCCGGGAAGAGGCGCGGGTAGAAGCGGGGGCTCTCCTCAGAGCTCGACGCATTTTTACTTTCCCTCTCATTTCTCTGACCGAAGCTGGGTGTCGGGCTTTCGCCTCTAGCGACTGGTGGAATTGCCTGCATCCGGGCCCCGGGCTTCCCGGCGGCGGCGGCGGCGGCGGCGGCGCAGGGACAAGGGATGGGGATCTGGCCTCTTCCTTGCTTTCCCGCCCTCAGTACCCGAGCTGTCTCCTTCCCGGGGACCCGCTGGGAGCGCTGCCGCTGCGGGCTCGAGAAAAGGGAGCCTCGGGTACTGAGAGGCCTCGCCTGGGGGAAGGCCGGAGGGTGGGCGGCGCGCGGCTTCTGCGGACCAAGTCGGGGTTCGCTAGGAACCCGAGACGGTCCCTGCCGGCGAGGAGATCATGCGGGATGAGATGGGGGTGTGGAGACGCCTGCACAATTTCAGCCCAAGCTTCTAGAGAGTGGTGATGACTTGCATATGAGGGCAGCAATGCAAGTCGGTGTGCTCCCCATTCTGTGGGACATGACCTGGTTGCTTCACAGCTCCGAGATGACACAGACTTGCTTAAAGGAAGTGACTATTGTGACTTGGGCATCACTTGACTGATGGTAATCAGTTGTCTAAAGAAGTGCACAGATTACATGTCCGTGTGCTCATTGGGTCTATCTGGCCGCGTTGAACACCACCAGGCTTTGTATTCAGAAACAGGAGGGAGGTCCTGCACTTTCCCAGGAGGGGTGGCCCTTTCAGATGCAATCGAGATTGTTAGGCTCTGGGAGAGTAGTTGCCTGGTTGTGGCAGTTGGTAAATTTCTATTCAAACAGTTGCCATGCACCAGTTGTTCACAACAAGGGTACGTAATCTGTCTGGCATTACTTCTACTTTTGTACAAAGGATCAAAAAAAAAAAAAAAGATACTGTTAAGATATGATTTTTCTCAGACTTTGGGAAACTTTTAACATAATCTGTGAATATCACAGAAACAAGACTATCATATAGGGGATATTAATAACCTGGAGTCAGAATACTTGAAATACGGTGTCATTTGACACGGGCATTGTTGTCACCACCTCTGCCAAGGCCTGCCACTTTAGGAAAACCCTGAATCAGTTGGAAACTGCTACATGCTGATAGTACATCTGAAACAAGAACGAGAGTAATTACCACATTCCAGATTGTTCACTAAGCCAGCATTTACCTGCTCCAGGAAAAAATTACAAGCACCTTATGAAGTTGATAAAATATTTTGTTTGGCTATGTTGGCACTCCACAATTTGCTTTCAGAGAAACAAAGTAAACCAAGGAGGACTTCTGTTTTTCAAGTCTGCCCTCGGGTTCTATTCTACGTTAATTAGATAGTTCCCAGGAGGACTAGGTTAGCCTACCTATTGTCTGAGAAACTTGGAACTGTGAGAAATGGCCAGATAGTGATATGAACTTCACCTTCCAGTCTTCCCTGATGTTGAAGATTGAGAAAGTGTTGTGAACTTTCTGGTACTGTAAACAGTTCACTGTCCTTGAAGTGGTCCTGGGCAGCTCCTGTTGTGGAAAGTGGACGGTTTAGGATCCTGCTTCTCTTTGGGCTGGGAGAAAATAAACAGCATGGTTACAAGTATTGAGAGCCAGGTTGGAGAAGGTGGCTTACACCTGTAATGCCAGAGCTTTGGGAGGCGGAGGCAAGAGGATCACTTGAAGCCAGGAGTTCAAGCTCAACCTGGGCAACGTAGACCCTGTCTCTACAAAAAATTAAAAACTTAGCCGGGCGTGGTGATGTGCACCTGTAGTCCTAGCTACTTGGGAGGCTGAGGCAGGAGGGTCATTTGAGCCCAAGAGTTTGAAGTTACCGAGAGCTATGATCCTGCCAGTGCATTCCAGCCTGGATGACAAAACGAGACCCTGTCTCTAAAAAACAAGAAGTGAGGGCTTTATGATTGTAGAATTTTCACTACATAGCAGTGGACCAACCC

**Supplemental data 10. Plasmid backbones used to separate reads.**

>pAG3_barcode_1

CGTGCGTGTGTGTGTGCGTGGGGAGCGCCGCGTGCGGCCCGCGCTGCCCGGCGGCTGTGAGCGCTGCGGGCGCGGCGCGGGGCTTTGTGCGCTCCGCGTGTGCGCGAGGGGAGCGCGGCCGGGGGCGGTGCCCCGCGGTGCGGGGGGGCTGCGAGGGGAACAAAGGCTGCGTGCGGGGTGTGTGCGTGGGGGGGTGAGCAGGGGGTGTGGGCGCGGCGGTCGGGCTGTAACCCCCCCCTGCACCCCCCTCCCCGAGTTGCTGAGCACGGCCCGGCTTCGGGTGCGGGGCTCCGTGCGGGGCGTGGCGCGGGGCTCGCCGTGCCGGGCGGGGGGTGGCGGCAGGTGGGGGTGCCGGGCGGGGCGGGGCCGCCTCGGGCCGGGGAGGGCTCGGGGGAGGGGCGCGGCGGCCCCGGAGCGCCGGCGGCTGTCGAGGCGCGGCGAGCCGCAGCCATTGCCTTTTATGGTAATCGTGCGAGAGGGCGCAGGGACTTCCTTTGTCCCAAATCTGGCGGAGCCGAAATCTGGGAGGCGCCGCCGCACCCCCTCTAGCGGGCGCGGGCGAAGCGGTGCGGCGCCGGCAGGAAGGAAATGGGCGGGGAGGGCCTTCGTGCGTCGCCGCGCCGCCGTCCCCTTCTCCATCTCCAGCCTCGGGGCTGCCGCAGGGGGACGGCTGCCTTCGGGGGGGACGGGGCAGGGCGGGGTTCGGCTTCTGGCGTGTGACCGGCGGGTCTAGACCTCTGCTAACCATGTTCATGCCTTCTTCTCTTTCCTACAGCTCCTGGGCAACGTGCTGGTTGTTGTGCTGTCTCATCATTTTGGCAAAGA

>pAG3_barcode_2

CGCCTGGACCAGACCCCACGCAACGCCCAAAATAATAACCCCCACGAACCATAAACCATTCCCCATGGGGGACCCCGTCCCTAACCCACGGGGCCAGTGGCTATGGCAGGGCCTGCCGCCCCGACGTTGGCTGCGAGCCCTGGGCCTTCACCCGAACTTGGGGGGTGGGGTGGGGAAAAGGAAGAAACGCGGGCGTATTGGCCCCAATGGGGTCTCGGTGGGGTATCGACAGAGTGCCAGCCCTGGGACCGAACCCCGCGTTTATGAACAAACGACCCAACACCCGTGCGTTTTATTCTGTCTTTTTATTGCCGTCATAGCGCGGGTTCCTTCCGGTATTGTCTCCTTCCGTGTTTCAGTTAGCCTCCCCCATCTCCCCTATTCCTTTGCCCTCGGACGAGTGCTGGGGCGTCGGTTTCCACTATCGGCGAGTACTTCTACACAGCCATCGGTCCAGACGGCCGCGCTTCTGCGGGCGATTTGTGTACGCCCGACAGTCCCGGCTCCGGATCGGACGATTGCGTCGCATCGACCCTGCGCCCAAGCTGCATCATCGAAATTGCCGTCAACCAAGCTCTGATAGAGTTGGTCAAGACCAATGCGGAGCATATACGCCCGGAGCCGCGGCGATCCTGCAAGCTCCGGATGCCTCCGCTCGAAGTAGCGCGTCTGCTGCTCCATACAAGCCAACCACGGCCTCCAGAAGAAGATGTTGGCGACCTCGTATTGGGAATCCCCGAACATCGCCTCGCTCCAGTCAATGACCGCTGTTATGCGGCCATTGTCCGTCAGGACATTGTTGGAGCCGAAATCCGCGTGCACGAGGTGCCGGACTTCGGGGCAGTCCTCGGCCCAAAGCATCAGCTCATCGAGAGCCTGCGCGACGGACGCACTGACGGTGTCGTCCATCACAGTTTGCCAGTGATACACATGGGGATCAGCAATCGCGCATATGAAATCACGCCATGTAGTGTATTGACCGATTCCTTGCGGTCCGAATGGGCCGAACCCGCTCGTCTGGCTAAGATCGGCCGCAGCGATCGCATCCATGGCCTCCGCGACCGGCTGCAGAACAGCGGGCAGTTCGGTTTCAGGCAGGTCTTGCAACGTGACACCCTGTGCACGGCGGGAGATGCAATAGGTCAGGCTCTCGCTGAATTCCCCAATGTCAAGCACTTCCGGAATCGGGAGCGCGGCCGATGCAAAGTGCCGATAAACATAACGATCTTTGTAGAAACCATCGGCGCAGCTATTTACCCGCAGGACATATCCACGCCCTCCTACATCGAAGCTGAAAGCACGAGATTCTTCGCCCTCCGAGAGCTGCATCAGGTCGGAGACGCTGTCGAACTTTTCGATCAGAAACTTCTCGACAGACGTCGCGGTGAGTTCAGGCTTTTTCATATCTCATTGCCCGGGATCTGCGGCACGCTGTTGACGCTGTTAAGCGGGTCGCTGCAGGGTCGC

>pc_C9-774R_barcode_1

AGGCTTTTGCAAAAAGCTCCCGGGAGCTTGTATATCCATTTTCGGATCTGATCAGCACGTGTTGACAATTAATCATCGGCATAGTATATCGGCATAGTATAATACGACAAGGTGAGGAACTAAACCATGGCCAAGCCTTTGTCTCAAGAAGAATCCACCCTCATTGAAAGAGCAACGGCTACAATCAACAGCATCCCCATCTCTGAAGACTACAGCGTCGCCAGCGCAGCTCTCTCTAGCGACGGCCGCATCTTCACTGGTGTCAATGTATATCATTTTACTGGGGGACCTTGTGCAGAACTCGTGGTGCTGGGCACTGCTGCTGCTGCGGCAGCTGGCAACCTGACTTGTATCGTCGCGATCGGAAATGAGAACAGGGGCATCTTGAGCCCCTGCGGACGGTGCCGACAGGTGCTTCTCGATCTGCATCCTGGGATCAAAGCCATAGTGAAGGACAGTGATGGACAGCCGACGGCAGTTGGGATTCGTGAATTGCTGCCCTCTGGTTATGTGTGGGAGGGCTAAGCACTTCGTGGCCGAGGAGCAGGACTGACACGTGCTACGAGATTTCGATTCCACCGCCGCCTTCTATGAAAGGTTGGGCTTCGGAATCGTTTTCCGGGACGCCGGCTGGATGATCCTCCAGCGCGGGGATCTCATGCTGGAGTTCTTCGCCCACCCCAACTTGTTTATTGCAGCTTATAATGGTTACAAATAAAGCAATAGCATCACAAATTTCACAAATAAAGCATTTTTTTCA

>pc_C9-774R_barcode_2

TCAGTACAATCTGCTCTGATGCCGCATAGTTAAGCCAGTATCTGCTCCCTGCTTGTGTGTTGGAGGTCGCTGAGTAGTGCGCGAGCAAAATTTAAGCTACAACAAGGCAAGGCTTGACCGACAATTGCATGAAGAATCTGCTTAGGGTTAGGCGTTTTGCGCTGCTTCGCGATGTACGGGCCAGATATACGCGTTGACATTGATTATTGACTAGTTGAGGCCCTTTCGTCTTCACTCGAGTTTACTCCCTATCAGTGATAGAGAACGTATGTCGAGTTTACTCCCTATCAGTGATAGAGAACGATGTCGAGTTTACTCCCTATCAGTGATAGAGAACGTATGTCGAGTTTACTCCCTATCAGTGATAGAGAACGTATGTCGAGTTTACTCCCTATCAGTGATAGAGAACGTATGTCGAGTTTATCCCTATCAGTGATAGAGAACGTATGTCGAGTTTACTCCCTATCAGTGATAGAGAACGTATGTCGAGGTAGGCGTGTACGGTGGGAGGCCTATATAAGCAGAGCTCGTTTAGTGAACCGTCAGATCGCCAAGCTT

>pc_C9-774R_barcode_3

GGTGGGCTCTATGGCTTCTGAGGCGGAAAGAACCAGCTGGGGCTCTAGGGGGTATCCCCACGCGCCCTGTAGCGGCGCATTAAGCGCGGCGGGTGTGGTGGTTACGCGCAGCGTGACCGCTACACTTGCCAGCGCCCTAGCGCCCGCTCCTTTCGCTTTCTTCCCTTCCTTTCTCGCCACGTTCGCCGGCTTTCCCCGTCAAGCTCTAAATCGGGGGCTCCCTTTAGGGTTCCGATTTAGTGCTTTACGGCACCTCGACCCCAAAAAACTTGATTAGGGTGATGGTTCACGTAGTGGGCCATCGCCCTGATAGACGGTTTTTCGCCCTTTGACGTTGGAGTCCACGTTCTTTAATAGTGGACTCTTGTTCCAAACTGGAACAACACTCAACCCTATCTCGGTCTATTCTTTTGATTTATAAGGGATTTTGCCGATTTCGGCCTATTGGTTAAAAAATGAGCTGATTTAACAAAAATTTAACGCGAATTA

>pAAV_barcode

GAAACCATTATTATCATGACATTAACCTATAAAAATAGGCGTATCACGAGGCCCTTTCGTCTCGCGCGTTTCGGTGATGACGGTGAAAACCTCTGACACATGCAGCTCCCGGAGACGGTCACAGCTTGTCTGTAAGCGGATGCCGGGAGCAGACAAGCCCGTCAGGGCGCGTCAGCGGGTGTTGGCGGGTGTCGGGGCTGGCTTAACTATGCGGCATCAGAGCAGATTGTACTGAGAGTGCACCATTCGACGCTCTCCCTTATGCGACTCCTGCATTAGGAAGCAGCCCAGTAGTAGGTTGAGGCCGTTGAGCACCGCCGCCGCAAGGAATGGTGCATGCAAGGAGATGGCGCCCAACAGTCCCCCGGCCACGGGGCCTGCCACCATACCCACGCCGAAACAAGCGCTCATGAGCCCGAAGTGGCGAGCCCGATCTTCCCCATCGGTGATGTCGGCGATATAGGCGCCAGCAACCGCACCTGTGGCGCCGGTGATGCCGGCCACGATGCGTCCGGCGTAGAGGATCTGGCTAGCGATGACCCTGCTGATTGGTTCGCTGACCATTTCCGGGTGCGGGACGGCGTTACCAGAAACTCAGAAGGTTCGTCCAACCAAACCGACTCTGACGGCAGTTTACGAGAGAGATGATAGGGTCTGCTTCAGTAAGCCAGATGCTACACAATTAGGCTTGTACATATTGTCGTTAGAACGCGGCTACAATTAATACATAACCTTATGTATCATACACATACGATTTAGGTGACACTATAGAATACACGGAATTAATTCTAGCTGCGCGCTCGCTCGCTCACTGAGGCCGCCCGGGCAAAGCCCGGGCGTCGGGCGACCTTTGGTCGCCCGGCCTCAGTGAGCGAGCGAGCGCGCAGAGAGGGAGTGGCCAACTCCATCACTAGGGGTTCCTTGTAGTTAATGATTAACCCGCCATGCTACTTATCTACGTAGCCATGCTCT

>pFdelta6_barcode_1

GGCAAAATAGCACCCTCCCGCTCCAGAACAACATACAGCGCTTCACAGCGGCAGCCTAACAGTCAGCCTTACCAGTAAAAAAGAAAACCTATTAAAAAAACACCACTCGACACGGCACCAGCTCAATCAGTCACAGTGTAAAAAAGGGCCAAGTGCAGAGCGAGTATATATAGGACTAAAAAATGACGTAACGGTTAAAGTCCACAAAAAACACCCAGAAAACCGCACGCGAACCTACGCCCAGAAACGAAAGCCAAAAAACCCACAACTTCCTCAAATCGTCACTTCCGTTTTCCCACGTTACGTAACTTCCCATTTTAAGAAAACTACAATTCCCAACACATACAAGTTACTCCGCCCTAAAACCTACGTCACCCGCCCCGTTCCCACGCCCCGCGCCACGTCACAAACTCCACCCCCTCATTATCATATTGGCTTCAATCCAAAATAAGGTATATTATTGATGATTTATTTTGGATTGAAGCCAATATGATAATGAGGGGGTGGAGTTTGTGACGTGGCGCGGGGCGTGGGAACGGGGCGGGTGACGTAGTAGTGTGGCGGAAGTGTGATGTTGCAAGTGTGGCGGAACACATGTAAGCGACGGATGTGGCAAAAGTGACGTTTTTGGTGTGCGCCGGATCCACAGGACGGGTGTGGTCGCCATGATCGCGTAGTCGATAGTGGCTCCAAGTAGCGAAGCGAGCAGGACTGGGCGGCGGCCAAAGCGGTCGGACAGTGCTCCGAGAACGGGTGCGCATAGAAATTGCATCAACGCATATAGCGCTAGCAGCACGCCATAGTGACTGGCGATGCTGTCGGAATGGACGATATCCCGCAAGAGGCCCGGCAGTACCGGCATAACCAAGCCTATGCCTACAGCATCCAGGGTGACGGTGCCGAGGATGACGATGAGCGCATTGTTAGATTTCATACACGGTGCCTGACTGCGTTAGCAATTTAACTGTGATAAACTACCGCATTAAAGCTTATCGATGATAAGCTGTCAAACATGAGA

>pFdelta6_barcode_2

CACCGCGGTGGCGGCCGCTCTAGAACTAGTGGATCCCCCGGGCTGCAGGAATTCGATATCAAGCTTATCGATACCGTCGACAGAAGCACCATGTCCTTGGGTCCGGCCTGCTGAATGCGCAGGCGGTCGGCCATGCCCCAGGCTTCGTTTTGACATCGGCGCAGGTCTTTGTAGTAGTCTTGCATGAGCCTTTCTACCGGCACTTCTTCTTCTCCTTCCTCTTGTCCTGCATCTCTTGCATCTATCGCTGCGGCGGCGGCGGAGTTTGGCCGTAGGTGGCGCCCTCTTCCTCCCATGCGTGTGACCCCGAAGCCCCTCATCGGCTGAAGCAGGGCTAGGTCGGCGACAACGCGCTCGGCTAATATGGCCTGCTGCACCTGCGTGAGGGTAGACTGGAAGTCATCCATGTCCACAAAGCGGTGGTATGCGCCCGTGTTGATGGTGTAAGTGCAGTTGGCCATAACGGACCAGTTAACGGTCTGGTGACCCGGCTGCGAGAGCTCGGTGTACCTGAGACGCGAGTAAGCCCTCGAGTCAAATACGTAGTCGTTGCAAGTCCGCACCAGGTACTGGTATCCCACCAAAAAGTGCGGCGGCGGCTGGCGGTAGAGGGGCCAGCGTAGGGTGGCCGGGGCTCCGGGGGCGAGATCTTCCAACATAAGGCGATGATATCCGTAGATGTACCTGGACATCCAGGTGATGCCGGCGGCGGTGGTGGAGGCGCGCGGAAAGTCGCGGACGCGGTTCCAGATGTTGCGCAGCGGCAAAAAGTGCTCCATGGTCGGGACGCTCTGGCCGGTCAGGCGCGCGCAATCGTTGACGCTCTACCGTGCAAAAGGAGAGCCTGTAAGCGGGCACTCTTCCGTGGTCTGGTGGATAAATTCGCAAGGGTATCATGGCGGACGACCGGGGTTCGAGCCCCGTATCCGGCCGTCCGCCGTGATCCATGCGGTTACCGCCCGCGTGTCGAACCCAGGTGTGCGACGTCAGACAACGGGGGAGTGCTCCTTTTGGCTTCCTTCCAGGCGCGGCGGCTGCTGCGCTAGCTTTTTTGGCCACTGGCCGCGCGCAGCGTAAGCGGTTAGGCTGGAAAGCGAAAGCATTAAGTGGCTCGCTCCCTGTAGCCGGAGGGTTATTTTCCAAGGGTTGAGTCGCGGGACCCCCGGTTCGAGTCTCGGACCGAGACTGGGGGCGTACACTGGATGGCCTTTGCCTGGAACCCGCACTCAAAAACATGCTACCTCTTTGAGCCCTTTGGCTTTTCTGACCAGCGACTCAAGCAGGTTTACCAGTTTGAGTACGAGTCACTCCTGCGCCGTAGCGCCATTGCTTCTTCCCCCGACCGCTGTATAACGCTGGAAAAGTCCACCCAAAGCGTACAGGGGCCCAACTCGGCCGCCTGTGGACTATTCTGCTGCATGTTTCTCCACGCCTTTGCCAACTGGCCCCAAACTCCCATGGATCACAACCCCACCATGAACCTTATTACCGGGGTACCCAACTCCATGCTCAACAGTCCCCAGGTACAGCCCACCCTGCGTCGCAACCAGGAACAGCTCTACAGCTTCCTGGAGCGCCACTCGCCCTACTTCCGCAGCCACAGTGCGCAGATTAGGAGCGCCACTTCTTTTTGTCACTTGAAAAACATGTAAAAATAATGTACTAGAGACACTTTCAATAAAGGCAAATGCTTTTATTTGTACACTCTCGGGTGATTATTTACCCCCACCCTTGCCGTCTGCGCCGTTTAAAAATCAAAGGGGTTCTGCCGCGCATCGCTATGCGCCACTGGCAGGGACACGTTGCGATACTGGTGTTTAGTGCTCCACTTAAACTCAGGCACAACCATCCGCGGCAGCTCGGTGAAGTTTTCACTCCACAGGCTGCGCACCATCACCAACGCGTTTAGCAGGTCGGGCGCCGATATCTTGAAGTCGCAGTTGGGGCCTCCGCCCTGCGCGCGCGAGTTGCGATACACAGGGTTGCAGCACTGGAACACTATCAGCGCCGGGTGGTGCACGCTGGCCAGCACGCTCTTGTCGGAGATCAGATCCGCGTCCAGGTCCTCCGCGTTGCTCAGGGCGAACGGAGTCAACTTTGGTAGCTGCCTTCCCAAAAAGGGCGCGTGCCCAGGCTTTGAGTTGCACTCGCACCGTAGTGGCATCAAAAGGTGACCGTGCCCGGTCTGGGCGTTAGGATACAGCGCCTGCATAAAAGCCTTGATCTGCTTAAAAGCCACCTGAGCCTTTGCGCCTTCAGAGAAGAACATGCCGCAAGACTTGCCGGAAAACTGATTGGCCGGACAGGCCGCGTCGTGCACGCAGCACCTTGCGTCGGTGTTGGAGATCTGCACCACATTTCGGCCCCACCGGTTCTTCACGATCTTGGCCTTGCTAGACTGCTCCTTCAGCGCGCGCTGCCCGTTTTCGCTCGTCACATCCATTTCAATCACGTGCTCCTTATTTATCATAATGCTTCCGTGTAGACACTTAAGCTCGCCTTCGATCTCAGCGCAGCGGTGCAGCCACAACGCGCAGCCCGTGGGCTCGTGATGCTTGTAGGTCACCTCTGCAAACGACTGCAGGTACGCCTGCAGGAATCGCCCCATCATCGTCACAAAGGTCTTGTTGCTGGTGAAGGTCAGCTGCAACCCGCGGTGCTCCTCGTTCAGCCAGGTCTTGCATACGGCCGCCAGAGCTTCCACTTGGTCAGGCAGTAGTTTGAAGTTCGCCTTTAGATCGTTATCCACGTGGTACTTGTCCATCAGCGCGCGCGCAGCCTCCATGCCCTTCTCCCACGCAGACACGATCGGCACACTCAGCGGGTTCATCACCGTAATTTCACTTTCCGCTTCGCTGGGCTCTTCCTCTTCCTCTTGCGTCCGCATACCACGCGCCACTGGGTCGTCTTCATTCAGCCGCCGCACTGTGCGCTTACCTCCTTTGCCATGCTTGATTAGCACCGGTGGGTTGCTGAAACCCACCATTTGTAGCGCCACATCTTCTCTTTCTTCCTCGCTGTCCACGATTACCTCTGGTGATGGCGGGCGCTCGGGCTTGGGAGAAGGGCGCTTCTTTTTCTTCTTGGGCGCAATGGCCAAATCCGCCGCCGAGGTCGATGGCCGCGGGCTGGGTGTGCGCGGCACCAGCGCGTCTTGTGATGAGTCTTCCTCGTCCTCGGACTCGATACGCCGCCTCATCCGCTTTTTTGGGGGCGCCCGGGGAGGCGGCGGCGACGGGGACGGGGACGACACGTCCTCCATGGTTGGGGGACGTCGCGCCGCACCGCGTCCGCGCTCGGGGGTGGTTTCGCGCTGCTCCTCTTCCCGACTGGCCATTTCCTTCTCCTATAGGCAGAAAAAGATCATGGAGTCAGTCGAGAAGAAGGACAGCCTAACCGCCCCCTCTGAGTTCGCCACCACCGCCTCCACCGATGCCGCCAACGCGCCTACCACCTTCCCCGTCGAGGCACCCCCGCTTGAGGAGGAGGAAGTGATTATCGAGCAGGACCCAGGTTTTGTAAGCGAAGACGACGAGGACCGCTCAGTACCAACAGAGGATAAAAAGCAAGACCAGGACAACGCAGAGGCAAACGAGGAACAAGTCGGGCGGGGGGACGAAAGGCATGGCGACTACCTAGATGTGGGAGACGACGTGCTGTTGAAGCATCTGCAGCGCCAGTGCGCCATTATCTGCGACGCGTTGCAAGAGCGCAGCGATGTGCCCCTCGCCATAGCGGATGTCAGCCTTGCCTACGAACGCCACCTATTCTCACCGCGCGTACCCCCCAAACGCCAAGAAAACGGCACATGCGAGCCCAACCCGCGCCTCAACTTCTACCCCGTATTTGCCGTGCCAGAGGTGCTTGCCACCTATCACATCTTTTTCCAAAACTGCAAGATACCCCTATCCTGCCGTGCCAACCGCAGCCGAGCGGACAAGCAGCTGGCCTTGCGGCAGGGCGCTGTCATACCTGATATCGCCTCGCTCAACGAAGTGCCAAAAATCTTTGAGGGTCTTGGACGCGACGAGAAGCGCGCGGCAAACGCTCTGCAACAGGAAAACAGCGAAAATGAAAGTCACTCTGGAGTGTTGGTGGAACTCGAGGGTGACAACGCGCGCCTAGCCGTACTAAAACGCAGCATCGAGGTCACCCACTTTGCCTACCCGGCACTTAACCTACCCCCCAAGGTCATGAGCACAGTCATGAGTGAGCTGATCGTGCGCCGTGCGCAGCCCCTGGAGAGGGATGCAAATTTGCAAGAACAAACAGAGGAGGGCCTACCCGCAGTTGGCGACGAGCAGCTAGCGCGCTGGCTTCAAACGCGCGAGCCTGCCGACTTGGAGGAGCGACGCAAACTAATGATGGCCGCAGTGCTCGTTACCGTGGAGCTTGAGTGCATGCAGCGGTTCTTTGCTGACCCGGAGATGCAGCGCAAGCTAGAGGAAACATTGCACTACACCTTTCGACAGGGCTACGTACGCCAGGCCTGCAAGATCTCCAACGTGGAGCTCTGCAACCTGGTCTCCTACCTTGGAATTTTGCACGAAAACCGCCTTGGGCAAAACGTGCTTCATTCCACGCTCAAGGGCGAGGCGCGCCGCGACTACGTCCGCGACTGCGTTTACTTATTTCTATGCTACACCTGGCAGACGGCCATGGGCGTTTGGCAGCAGTGCTTGGAGGAGTGCAACCTCAAGGAGCTGCAGAAACTGCTAAAGCAAAACTTGAAGGACCTATGGACGGCCTTCAACGAGCGCTCCGTGGCCGCGCACCTGGCGGACATCATTTTCCCCGAACGCCTGCTTAAAACCCTGCAACAGGGTCTGCCAGACTTCACCAGTCAAAGCATGTTGCAGAACTTTAGGAACTTTATCCTAGAGCGCTCAGGAATCTTGCCCGCCACCTGCTGTGCACTTCCTAGCGACTTTGTGCCCATTAAGTACCGCGAATGCCCTCCGCCGCTTTGGGGCCACTGCTACCTTCTGCAGCTAGCCAACTACCTTGCCTACCACTCTGACATAATGGAAGACGTGAGCGGTGACGGTCTACTGGAGTGTCACTGTCGCTGCAACCTATGCACCCCGCACCGCTCCCTGGTTTGCAATTCGCAGCTGCTTAACGAAAGTCAAATTATCGGTACCTTTGAGCTGCAGGGTCCCTCGCCTGACGAAAAGTCCGCGGCTCCGGGGTTGAAACTCACTCCGGGGCTGTGGACGTCGGCTTACCTTCGCAAATTTGTACCTGAGGACTACCACGCCCACGAGATTAGGTTCTACGAAGACCAATCCCGCCCGCCAAATGCGGAGCTTACCGCCTGCGTCATTACCCAGGGCCACATTCTTGGCCAATTGCAAGCCATCAACAAAGCCCGCCAAGAGTTTCTGCTACGAAAGGGACGGGGGGTTTACTTGGACCCCCAGTCCGGCGAGGAGCTCAACCCAATCCCCCCGCCGCCGCAGCCCTATCAGCAGCAGCCGCGGGCCCTTGCTTCCCAGGATGGCACCCAAAAAGAAGCTGCAGCTGCCGCCGCCACCCACGGACGAGGAGGAATACTGGGACAGTCAGGCAGAGGAGGTTTTGGACGAGGAGGAGGAGGACATGATGGAAGACTGGGAGAGCCTAGACGAGGAAGCTTCCGAGGTCGAAGAGGTGTCAGACGAAACACCGTCACCCTCGGTCGCATTCCCCTCGCCGGCGCCCCAGAAATCGGCAACCGGTTCCAGCATGGCTACAACCTCCGCTCCTCAGGCGCCGCCGGCACTGCCCGTTCGCCGACCCAACCGTAGATGGGACACCACTGGAACCAGGGCCGGTAAGTCCAAGCAGCCGCCGCCGTTAGCCCAAGAGCAACAACAGCGCCAAGGCTACCGCTCATGGCGCGGGCACAAGAACGCCATAGTTGCTTGCTTGCAAGACTGTGGGGGCAACATCTCCTTCGCCCGCCGCTTTCTTCTCTACCATCACGGCGTGGCCTTCCCCCGTAACATCCTGCATTACTACCGTCATCTCTACAGCCCATACTGCACCGGCGGCAGCGGCAGCGGCAGCAACAGCAGCGGCCACACAGAAGCAAAGGCGACCGGATAGCAAGACTCTGACAAAGCCCAAGAAATCCACAGCGGCGGCAGCAGCAGGAGGAGGAGCGCTGCGTCTGGCGCCCAACGAACCCGTATCGACCCGCGAGCTTAGAAACAGGATTTTTCCCACTCTGTATGCTATATTTCAACAGAGCAGGGGCCAAGAACAAGAGCTGAAAATAAAAAACAGGTCTCTGCGATCCCTCACCCGCAGCTGCCTGTATCACAAAAGCGAAGATCAGCTTCGGCGCACGCTGGAAGACGCGGAGGCTCTCTTCAGTAAATACTGCGCGCTGACTCTTAAGGACTAGTTTCGCGCCCTTTCTCAAATTTAAGCGCGAAAACTACGTCATCTCCAGCGGCCACACCCGGCGCCAGCACCTGTCGTCAGCGCCATTATGAGCAAGGAAATTCCCACGCCCTACATGTGGAGTTACCAGCCACAAATGGGACTTGCGGCTGGAGCTGCCCAAGACTACTCAACCCGAATAAACTACATGAGCGCGGGACCCCACATGATATCCCGGGTCAACGGAATCCGCGCCCACCGAAACCGAATTCTCTTGGAACAGGCGGCTATTACCACCACACCTCGTAATAACCTTAATCCCCGTAGTTGGCCCGCTGCCCTGGTGTACCAGGAAAGTCCCGCTCCCACCACTGTGGTACTTCCCAGAGACGCCCAGGCCGAAGTTCAGATGACTAACTCAGGGGCGCAGCTTGCGGGCGGCTTTCGTCACAGGGTGCGGTCGCCCGGGCAGGGTATAACTCACCTGACAATCAGAGGGCGAGGTATTCAGCTCAACGACGAGTCGGTGAGCTCCTCGCTTGGTCTCCGTCCGGACGGGACATTTCAGATCGGCGGCGCCGGCCGTCCTTCATTCACGCCTCGTCAGGCAATCCTAACTCTGCAGACCTCGTCCTCTGAGCCGCGCTCTGGAGGCATTGGAACTCTGCAATTTATTGAGGAGTTTGTGCCATCGGTCTACTTTAACCCCTTCTCGGGACCTCCCGGCCACTATCCGGATCAATTTATTCCTAACTTTGACGCGGTAAAGGACTCGGCGGACGGCTACGACTGAATGTTAAGTGGAGAGGCAGAGCAACTGCGCCTGAAACACCTGGTCCACTGTCGCCGCCACAAGTGCTTTGCCCGCGACTCCGGTGAGTTTTGCTACTTTGAATTGCCCGAGGATCATATCGAGGGCCCGGCGCACGGCGTCCGGCTTACCGCCCAGGGAGAGCTTGCCCGTAGCCTGATTCGGGAGTTTACCCAGCGCCCCCTGCTAGTTGAGCGGGACAGGGGACCCTGTGTTCTCACTGTGATTTGCAACTGTCCTAACCTTGGATTACATCAAGATCCTCTAGTTAATTAACTAGAGTACCCGGGGATCTTATTCCCTTTAACTAATAAAAAAAAATAATAAAGCATCACTTACTTAAAATCAGTTAGCAAATTTCTGTCCAGTTTATTCAGCAGCACCTCCTTGCCCTCCTCCCAGCTCTGGTATTGCAGCTTCCTCCTGGCTGCAAACTTTCTCCACAATCTAAATGGAATGTCAGTTTCCTCCTGTTCCTGTCCATCCGCACCCACTATCTTCATGTTGTTGCAGATGAAGCGCGCAAGACCGTCTGAAGATACCTTCAACCCCGTGTATCCATATGACACGGAAACCGGTCCTCCAACTGTGCCTTTTCTTACTCCTCCCTTTGTATCCCCCAATGGGTTTCAAGAGAGTCCCCCTGGGGTACTCTCTTTGCGCCTATCCGAACCTCTAGTTACCTCCAATGGCATGCTTGCGCTCAAAATGGGCAACGGCCTCTCTCTGGACGAGGCCGGCAACCTTACCTCCCAAAATGTAACCACTGTGAGCCCACCTCTCAAAAAAACCAAGTCAAACATAAACCTGGAAATATCTGCACCCCTCACAGTTACCTCAGAAGCCCTAACTGTGGCTGCCGCCGCACCTCTAATGGTCGCGGGCAACACACTCACCATGCAATCACAGGCCCCGCTAACCGTGCACGACTCCAAACTTAGCATTGCCACCCAAGGACCCCTCACAGTGTCAGAAGGAAAGCTAGCCCTGCAAACATCAGGCCCCCTCACCACCACCGATAGCAGTACCCTTACTATCACTGCCTCACCCCCTCTAACTACTGCCACTGGTAGCTTGGGCATTGACTTGAAAGAGCCCATTTATACACAAAATGGAAAACTAGGACTAAAGTACGGGGCTCCTTTGCATTAACAGACGACCTAAACACTTTGACCGTAGCAACTGGTCCAGGTGTGACTATTAATAATACTTCCTTGCAAACTAAAGTTACTGGAGCCTTGGGTTTTGATTCACAAGGCAATATGCAACTTAATGTAGCAGGAGGACTAAGGATTGATTCTCAAAACAGACGCCTTATACTTGATGTTAGTTATCCGTTTGATGCTCAAAACCAACTAAATCTAAGACTAGGACAGGGCCCTCTTTTTATAAACTCAGCCCACAACTTGGATATTAACTACAACAAAGGCCTTTACTTGTTTACAGCTTCAAACAATTCCAAAAAGCTTGAGGTTAACCTAAGCACTGCCAAGGGGTTGATGTTTGACGCTACAGCCATAGCCATTAATGCAGGAGATGGGCTTGAATTTGGTTCACCTAATGCACCAAACACAAATCCCCTCAAAACAAAAATTGGCCATGGCCTAGAATTTGATTCAAACAAGGCTATGGTTCCTAAACTAGGAACTGGCCTTAGTTTTGACAGCACAGGTGCCATTACAGTAGGAAACAAAAATAATGATAAGCTAACTTTGTGGACCACACCAGCTCCATCTCCTAACTGTAGACTAAATGCAGAGAAAGATGCTAAACTCACTTTGGTCTTAACAAAATGTGGCAGTCAAATACTTGCTACAGTTTCAGTTTTGGCTGTTAAAGGCAGTTTGGCTCCAATATCTGGAACAGTTCAAAGTGCTCATCTTATTATAAGATTTGACGAAAATGGAGTGCTACTAAACAATTCCTTCCTGGACCCAGAATATTGGAACTTTAGAAATGGAGATCTTACTGAAGGCACAGCCTATACAAACGCTGTTGGATTTATGCCTAACCTATCAGCTTATCCAAAATCTCACGGTAAAACTGCCAAAAGTAACATTGTCAGTCAAGTTTACTTAAACGGAGACAAAACTAAACCTGTAACACTAACCATTACACTAAACGGTACACAGGAAACAGGAGACACAACTCCAAGTGCATACTCTATGTCATTTTCATGGGACTGGTCTGGCCACAACTACATTAATGAAATATTTGCCACATCCTCTTACACTTTTTCATACATTGCCCAAGAATAAAGAATCGTTTGTGTTATGTTTCAACGTGTTTATTTTTCAATTGCAGAAAATTTCAAGTCATTTTTCATTCAGTAGTATAGCCCCACCACCACATAGCTTATACAGATCACCGTACCTTAATCAAACTCACAGAACCCTAGTATTCAACCTGCCACCTCCCTCCCAACACACAGAGTACACAGTCCTTTCTCCCCGGCTGGCCTTAAAAAGCATCATATCATGGGTAACAGACATATTCTTAGGTGTTATATTCCACACGGTTTCCTGTCGAGCCAAACGCTCATCAGTGATATTAATAAACTCCCCGGGCAGCTCACTTAAGTTCATGTCGCTGTCCAGCTGCTGAGCCACAGGCTGCTGTCCAACTTGCGGTTGCTTAACGGGCGGCGAAGGAGAAGTCCACGCCTACATGGGGGTAGAGTCATAATCGTGCATCAGGATAGGGCGGTGGTGCTGCAGCAGCGCGCGAATAAACTGCTGCCGCCGCCGCTCCGTCCTGCAGGAATACAACATGGCAGTGGTCTCCTCAGCGATGATTCGCACCGCCCGCAGCATAAGGCGCCTTGTCCTCCGGGCACAGCAGCGCACCCTGATCTCACTTAAATCAGCACAGTAACTGCAGCACAGCACCACAATATTGTTCAAAATCCCACAGTGCAAGGCGCTGTATCCAAAGCTCATGGCGGGGACCACAGAACCCACGTGGCCATCATACCACAAGCGCAGGTAGATTAAGTGGCGACCCCTCATAAACACGCTGGACATAAACATTACCTCTTTTGGCATGTTGTAATTCACCACCTCCCGGTACCATATAAACCTCTGATTAAACATGGCGCCATCCACCACCATCCTAAACCAGCTGGCCAAAACCTGCCCGCCGGCTATACACTGCAGGGAACCGGGACTGGAACAATGACAGTGGAGAGCCCAGGACTCGTAACCATGGATCATCATGCTCGTCATGATATCAATGTTGGCACAACACAGGCACACGTGCATACACTTCCTCAGGATTACAAGCTCCTCCCGCGTTAGAACCATATCCCAGGGAACAACCCATTCCTGAATCAGCGTAAATCCCACACTGCAGGGAAGACCTCGCACGTAACTCACGTTGTGCATTGTCAAAGTGTTACATTCGGGCAGCAGCGGATGATCCTCCAGTATGGTAGCGCGGGTTTCTGTCTCAAAAGGAGGTAGACGATCCCTACTGTACGGAGTGCGCCGAGACAACCGAGATCGTGTTGGTCGTAGTGTCATGCCAAATGGAACGCCGGACGTAGTCATATTTCCTGAAGCAAAACCAGGTGCGGGCGTGACAAACAGATCTGCGTCTCCGGTCTCGCCGCTTAGATCGCTCTGTGTAGTAGTTGTAGTATATCCACTCTCTCAAAGCATCCAGGCGCCCCCTGGCTTCGGGTTCTATGTAAACTCCTTCATGCGCCGCTGCCCTGATAACATCCACCACCGCAGAATAAGCCACACCCAGCCAACCTACACATTCGTTCTGCGAGTCACACACGGGAGGAGCGGGAAGAGCTGGAAGAACCATGTTTTTTTTTTTATTCCAAAAGATTATCCAAAACCTCAAAATGAAGATCTATTAAGTGAACGCGCTCCCCTCCGGTGGCGTGGTCAAACTCTACAGCCAAAGAACAGATAATGGCATTTGTAAGATGTTGCACAATGGCTTCCAAAAGGCAAACGGCCCTCACGTCCAAGTGGACGTAAAGGCTAAACCCTTCAGGGTGAATCTCCTCTATAAACATTCCAGCACCTTCAACCATGCCCAAATAATTCTCATCTCGCCACCTTCTCAATATATCTCTAAGCAAATCCCGAATATTAAGTCCGGCCATTGTAAAAATCTGCTCCAGAGCGCCCTCCACCTTCAGCCTCAAGCAGCGAATCATGATTGCAAAAATTCAGGTTCCTCACAGACCTGTATAAGATTCAAAAGCGGAACATTAACAAAAATACCGCGATCCCGTAGGTCCCTTCGCAGGGCCAGCTGAACATAATCGTGCAGGTCTGCACGGACCAGCGCGGCCACTTCCCCGCCAGGAACCTTGACAAAAGAACCCACACTGATTATGACACGCATACTCGGAGCTATGCTAACCAGCGTAGCCCCGATGTAAGCTTTGTTGCATGGGCGGCGATATAAAATGCAAGGTGCTGCTCAAAAAATCAGGCAAAGCCTCGCGCAAAAAAGAAAGCACATCGTAGTCATGCTCATGCAGATAAAGGCAGGTAAGCTCCGGAACCACCACAGAAAAAGACACCATTTTTCTCTCAAACATGTCTGCGGGTTTCTGCATAAACACAAAATAAAATAACAAAAAAACATTTAAACATTAGAAGCCTGTCTTACAACAGGAAAAACAACCCTTATAAGCATAAGACGGACTACGGCCATGCCGGCGTGACCGTAAAAAAACTGGTCACCGTGATTAAAAAGCACCACCGACAGCTCCTCGGTCATGTCCGGAGTCATAATGTAAGACTCGGTAAACACATCAGGTTGATTCATCGGTCAGTGCTAAAAAGCGACCGAAATAGCCCGGGGGAATACATACCCGCAGGCG

**Supplemental data 11. Sequences adjacent to the *C9orf72* repeat region used to identify on-target reads from the PacBio No-Amp Targeted Sequencing approach.**

>c9orf72_chr9:27573414-27573528

GCCGCCTCCTCACTCACCCACTCGCCACCGCCTGCGCCTCCGCCGCCGCGGGCGCAGGCACCGCAACCGCAGCCCCGCCCCGGGCCCGCCCCCGGGCCCGCCCCGACCACGCCCC

>c9orf72_chr9:27573547-27573661

TAGCGCGCGACTCCTGAGTTCCAGAGCTTGCTACAGGCTGCGGTTGTTTCCCTCCTTGTTTTCTTCTGGTTAATCTTTATCAGGTCTTTTCTTGTTCACCCTCAGCGAGTACTGT
